# Distinct Contributions of TNF Receptor 1 and 2 to TNF-Induced Glomerular Inflammation in Mice

**DOI:** 10.1371/journal.pone.0068167

**Published:** 2013-07-15

**Authors:** Anela Taubitz, Martin Schwarz, Nuru Eltrich, Maja T. Lindenmeyer, Volker Vielhauer

**Affiliations:** 1 Nephrologisches Zentrum, Medizinische Klinik und Poliklinik IV, Klinikum der Universität München, Ludwig-Maximilians-University Munich, Munich, Germany; 2 Institute of Physiology, University of Zurich, Zurich, Switzerland; Emory University, United States of America

## Abstract

TNF is an important mediator of glomerulonephritis. The two TNF-receptors TNFR1 and TNFR2 contribute differently to glomerular inflammation in vivo, but specific mechanisms of TNFR-mediated inflammatory responses in glomeruli are unknown. We investigated their expression and function in murine kidneys, isolated glomeruli ex vivo, and glomerular cells in vitro. In normal kidney TNFR1 and TNFR2 were preferentially expressed in glomeruli. Expression of both TNFRs and TNF-induced upregulation of TNFR2 mRNA was confirmed in murine glomerular endothelial and mesangial cell lines. In vivo, TNF exposure rapidly induced glomerular accumulation of leukocytes. To examine TNFR-specific inflammatory responses in intrinsic glomerular cells but not infiltrating leukocytes we performed microarray gene expression profiling on intact glomeruli isolated from wildtype and *Tnfr*-deficient mice following exposure to soluble TNF ex vivo. Most TNF-induced effects were exclusively mediated by TNFR1, including induced glomerular expression of adhesion molecules, chemokines, complement factors and pro-apoptotic molecules. However, TNFR2 contributed to TNFR1-dependent mRNA expression of inflammatory mediators in glomeruli when exposed to low TNF concentrations. Chemokine secretion was absent in TNF-stimulated *Tnfr1*-deficient glomeruli, but also significantly decreased in glomeruli lacking TNFR2. In vivo, TNF-induced glomerular leukocyte infiltration was abrogated in *Tnfr1*-deficient mice, whereas *Tnfr2*-deficiency decreased mononuclear phagocytes infiltrates, but not neutrophils. These data demonstrate that activation of intrinsic glomerular cells by soluble TNF requires TNFR1, whereas TNFR2 is not essential, but augments TNFR1-dependent effects. Previously described TNFR2-dependent glomerular inflammation may therefore require TNFR2 activation by membrane-bound, but not soluble TNF.

## Introduction

Cytokines produced by intrinsic renal cells and infiltrating leukocytes are central mediators of inflammatory kidney diseases. Tumor necrosis factor-α (TNF) is involved in glomerular inflammation and scarring [1], [2]. Renal expression of TNF is up-regulated in experimental animals and patients with glomerulonephritis (GN) [3], [4], [5], [6]. TNF is produced by macrophages, T cells, neutrophils and endothelium. Furthermore, intrinsic renal cells, including mesangial cells, podocytes, and tubular epithelial cells produce TNF upon stimulation [4], [7], [8], [9], [10], [11].

The functional role of TNF in GN has been demonstrated in animal models. Systemic administration of TNF induced glomerular damage in rabbits [12] and exacerbated glomerular injury in rats with nephrotoxic serum nephritis (NTN), a model of immune complex-mediated GN [13]. TNF-deficient mice subjected to NTN demonstrated reduced proteinuria, glomerular crescent formation, infiltration of leukocytes, and expression of vascular adhesion molecules [14]. In bone marrow chimeric mice it was shown that intrinsic renal cells are the major source of TNF contributing to renal injury in the NTN model [15], [16]. TNF blockade also attenuated glomerular lesions and crescent formation in rats developing anti-glomerular basement membrane antibody-induced crescentic GN [17], [18], [19].

Biological activities of TNF are mediated through two functionally distinct TNF receptors (TNFR), TNFR1 (CD120a) and TNFR2 (CD120b). An increased glomerular expression of TNFR1 and TNFR2 has been suggested by animal and human biopsy studies of GN [6], [20]. We have previously identified distinct proinflammatory roles of the two TNFRs in the development of glomerular injury in murine NTN. *Tnfr1*-deficiency delayed the onset of GN, whereas deficiency of intrinsic renal cell-expressed TNFR2 abrogated glomerular injury following immune complex deposition [20]. Underlying mechanisms of these differential TNFR-mediated inflammatory responses in the glomerulus are not known.

With TNF receptor-targeted interventions becoming a potential therapeutic strategy to reduce glomerular inflammation, mechanisms of TNFR-specific inflammatory responses in glomeruli remain to be elucidated. Here, we characterize TNFR1- and TNFR2-dependent glomerular inflammatory pathways in murine kidneys in vivo, in isolated glomeruli ex vivo, and in murine mesangial cells in vitro. Microarray gene expression profiling on intact glomeruli isolated from wildtype and *Tnfr*-deficient mice demonstrated that soluble TNF mediates most inflammatory effects in glomeruli via TNFR1-dependent mRNA induction of proinflammatory mediators, including adhesion molecules and chemokines. TNFR2 contributed to TNFR1-dependent effects after stimulation with low TNF concentrations. In addition, we identified a posttranscriptional role of TNFR2 in enhancing chemokine secretion. Consistent with these in vitro results, we demonstrated a predominant role of TNFR1 in mediating soluble TNF-induced glomerular leukocyte accumulation in vivo, with a selective contribution of TNFR2 to the accumulation of glomerular mononuclear phagocytes. Together, these data suggest that activation of intrinsic glomerular cells by soluble TNF requires TNFR1, whereas TNFR2 augments TNFR1-dependent effects. In contrast, no exclusively TNFR2-dependent inflammatory pathways were identified in soluble TNF-stimulated glomeruli. These data suggest that mice deficient for renal cell-expressed TNFR2 are protected from GN in vivo [20] as a result from blocked TNFR2 activation by membrane-bound TNF, but not soluble TNF in intrinsic glomerular cells.

## Materials and Methods

### Animals

Mouse strains with targeted deletion of TNFR1 (*Tnfr1*−/−, B6.129-*Tnfrsf1a^tm1Mak^*) and 2 (*Tnfr2*−/−, B6.129-*Tnfrsf1b^tm1Mwm^*) were originally purchased from the Jackson Laboratory (Bar Harbor, ME, USA) and bred in our Central Animal Facility (University Hospital Innenstadt, Ludwig-Maximilians-University Munich) under specific pathogen-free conditions. *Tnfr1* and *2* double-deficient mice (*Tnfr1,2*−/−) and C57BL/6J wildtype controls were generated by cross-breeding of single *Tnfr*-deficient mice. Renal leukocyte infiltration was induced in 6 to 8 week old male mice by intraperitoneal injection of 5 µg recombinant murine TNF (Invitrogen, Karlsruhe, Germany). Perfused kidneys were harvested eight hours after TNF injection. Untreated male mice were used for paramagnetic isolation of glomeruli subsequently analyzed in ex vivo experiments. All experimental procedures involving mice were approved by the Ethical Committee of the Regierung von Oberbayern (AZ 55.2-1-54-2532.3-8-12).

### Paramagnetic Isolation of Glomeruli

Isolation of glomeruli from mouse kidneys was essentially performed as described by Takemoto and coworkers [21]. Briefly, M-450 tosylactivated, 4.5 µm diameter Dynabeads (Invitrogen, Karlsruhe, Germany) were inactivated according to the manufacturer`s instructions. Mice were perfused with 8×10^7^ paramagnetic Dynabeads diluted in 40 ml of phosphate-buffered saline (PBS; Pan Biotech, Aidenbach, Germany) through the heart with a constant pressure of 40 mmHg using a custom-made, pressure-controlled syringe. Kidneys were digested with 1 mg/ml collagenase A (Roche Diagnostics, Mannheim, Germany) in 1 ml Hanks` balanced salt solution (HBSS; Pan Biotech) at 37°C for 30 minutes. The digested tissue was passed through a 100 µm cell strainer (BD Biosciences, Heidelberg, Germany). The cell suspension containing glomeruli with trapped paramagnetic Dynabeads was placed into a magnetic particle concentrator (Invitrogen) for 7 minutes. The first supernatant was pipetted into a separate tube and stored on ice. The remaining tissue was resuspended in 10 ml PBS, and placed again into the magnetic particle concentrator for 5 minutes. The supernatant was removed and remaining paramagnetically labelled glomeruli were resuspended in 4 ml PBS and passed through a 100 µm cell strainer. This suspension was placed again in the magnetic particle separator for 5 minutes, the supernatant discarded, and the remaining tissue was washed three times with 10 ml PBS. Finally, purified glomeruli were resuspended in 1.5 ml of PBS. All procedures were performed on ice except the collagenase digestion at 37°C. Samples of all obtained fractions were viewed microscopically. The final paramagnetically labelled fraction contained highly purified glomeruli with a yield of approximately 10,000 glomeruli per mouse kidney and a purity >97% (intact glomeruli versus tubular fragments or single cells). As previously described, isolated glomeruli were lacking the Bowman`s capsule [21]. The first supernatant was free of glomeruli, but contained tubular fragments, single tubular cells and polymorphic interstitial cells.

### Cell Culture

A murine glomerular endothelial cell line was originally derived from tsA58 immorto mice [22] and generously provided by Dr. Nese Akis (Division of Microbiology, Uludag University, Bursa, Turkey). Cells were grown in DMEM medium with GlutaMax (Invitrogen) supplemented with 10% fetal calf serum (FCS; Biochrom, Berlin, Germany), 100 U/ml penicillin and 100 µg/ml streptomycin (PAA Laboratories, Pasching, Austria). The endothelial cells were characterized by positive immunostaining for CD31, von Willebrand factor, and cytokeratin 18. A murine mesangial cell line was maintained in DMEM supplemented with 2.5% FCS and penicillin-streptomycin as described [23]. Reaching 80% confluency cells were serum starved for 24 hours and subsequently stimulated with murine TNF (Invitrogen) or IFN-γ (PeproTech, Rocky Hill, NJ, USA) in DMEM for 5 hours (endothelial cells) or 24 hours (mesangial cells).

Primary murine mesangial cells (pMC) were prepared from C57BL/6J wildtype and *Tnfr*-deficient mice following paramagnetic isolation of glomeruli as previously described [24]. After five passages, pMC were characterized by positive immunostaining for α-smooth muscle actin and desmin, and by negative staining for CD31, von Willebrand factor and cytokeratin 18. Being 80% confluent pMC from passages 7–8 were serum starved for 24 hours and subsequently stimulated with indicated concentrations of murine TNF in RPMI 1640 for 12 hours.

### Glomerular Culture and Stimulation

After isolation 5,000 intact glomeruli were resuspended in 3 ml RPMI 1640 supplemented with 15% FCS, 15 mM HEPES buffer (Invitrogen), 0.66 U/ml insulin and penicillin-streptomycin, and were incubated in 6 well plates at 37°C for 24 hours. After serum starving for further 24 hours glomeruli were stimulated with indicated concentrations of murine TNF in RPMI 1640 for 12 or 24 hours.

### Microarray Studies

For microarray experiments 5,000 glomeruli isolated from male C57BL/6J wildtype and *Tnfr*-deficient mice were serum starved and subsequently stimulated with 50 ng/ml TNF. After 12 hours total RNA was prepared using RNeasy Mini Kit (Qiagen, Hilden, Germany). Integrity of each RNA sample was tested by denaturing agarose gel electrophoresis. Three independent preparations of 2 µg total RNA per genotype was used for biotin-labeled complementary RNA probe synthesis and hybridization of Affymetrix Mouse Genome 430 2.0 arrays according to the Affymetrix Expression Analysis Technical Manual. Triplicate arrays per genotype were scanned and analyzed using the Affymetrix GeneChip Operating Software (GCOS1.0). The complete data set was deposited into the Gene Expression Omnibus (GEO) database (http://www.ncbi.nlm.nih.gov/geo/; submission no. GSE43928).

For microarray analysis robust multichip average (RMA) [25] was performed. All twelve Affymetrix Microarray CEL files were normalized together using RMA Express, version 1.0 beta 2 (http://rmaexpress.bmbolstad.com/). For probe set annotation the default Mouse 430_2.cdf was used. Following normalized RMA, significance analysis of microarrays (SAM) was applied using the Microsoft Excel plugin of SAM, version 1.21 [26] to analyze differentially expressed transcripts in *Tnfr1,2*−/−, *Tnfr1*−/− and *Tnfr2*−/− glomeruli compared to wildtype. A q-value <5% was used to identify probe sets that detected transcipts with different expression levels in the analyzed groups. If the same gene was identified by multiple probe sets, fold-changes to wildtype were calculated from the averaged signal intensities of the respective probe sets. Genes with an at least 1.5-fold difference in mRNA abundance were considered as differentially expressed between groups.

### Ranking of Differentially Expressed Genes by Gene Ontology

The Database for Annotation, Visualization and Integrated Discovery (DAVID; accessible at http://david.abcc.ncifcrf.gov) [27], [28] was used to group functionally related genes. DAVID gene functional classification was applied to rank the overall importance of functional gene groups that reached an enrichment score of ≥1.3. To identify over-represented biological categories (gene ontology classes) DAVID functional annotation chart was applied using the functional annotation categories of the Gene Ontology Consortium for cellular components, biological processes, and molecular function [27], [29]. Based on the Expression Analysis Systematic Explorer (EASE) [30], annotation terms with EASE scores of p ≤0.05, fold enrichment ≥1.5, and at least 4 genes per group were considered significant for over-representation in the given gene list [27]. For all analysis, redundant probe sets for a given gene were removed before analysis to prevent statistical biasing.

### RNA Isolation and qRT-PCR

Total RNA from cells and isolated tissue was prepared using the RNeasy Mini Kit (Quiagen). Complementary DNA was generated from 500 ng of total RNA by reverse transcription (RT) using random priming and MMLV reverse transcriptase (Invitrogen).

TaqMan real-time PCR was performed on an AB Prism 7000 analyser (Applied Biosystems, Weiterstadt, Germany) using heat-activated TaqDNA polymerase (Amplitaq Gold, Applied Biosystems). For murine nephrin (NM_019459), VCAM-1 (NM_011693), P-selectin (NM_011347), and GAPDH (NM_008084) the following oligonucleotide primers (300 nmol/L) and hydrolysis probes (100 nmol/L) were used (all from Applied Biosystems): murine nephrin, sense primer 5′-ACCCTCCAGTTAACTTGTCTTTGG-3′, antisense primer 5′-ATGCAGCGG AGCCTTTGA-3′, fluorescence labelled probe (FAM) 5′-TCCAGCCTCTCTCC-3′; murine VCAM-1, sense primer 5′-AACCCAAACAGAGGCAGAGTGTAC-3′, antisense primer 5′-GACCCAGATGGTGGTTTCCTT-3′, fluorescence labelled probe (FAM) 5′-TGTCAACGTTGCCCC-3′, murine P-selectin, sense primer 5′-ATGAA CCCTGTTTTAAACGAAAGC-3′, antisense primer 5′-CTTGGTTGCTGCAGGAC ATG-3′, fluorescence labelled probe (FAM) 5′-ACACAGCCTCCTGCC-3′, and murine Gapdh, sense primer 5′-CATGGCCTTCCGTGTTCCTA-3′, antisense primer 5′-ATGCCTGCTTCACCACCTTCT-3′, fluorescence labelled probe (VIC) 5′-CCCAA TGTGTCCGTCGTGGATCTGA-3′: For murine FXYD2 domain-containing ion transport regulator 2 (Fxyd2; NM_007503), TNFR1 (NM_011609), TNFR2 (NM_011610), CCL2/MCP-1 (NM_011333), CCL3/MIP-1α (NM_011337), CCL5/RANTES (NM_013653), ICAM-1 (NM_010493), E-selectin (NM_011693), and 18S rRNA commercially available pre-developed TaqMan reagents (Applied Biosystems) were used. For some target genes amplification and PCR product detection was performed using SYBR Green PCR Master Mix reagents. The SYBR Green forward (f) and reverse (r) oligonucleotide primer sequences were as follows: murine Rab6B, 5′- GGTTGCCTGGTAGGTGTTGT-3′ (f), 5′- GCTGCGAAAATTC AAGTTGG-3′ (r); and 18S rRNA, 5′-GCAATTATTCCCCATGAACG-3′ (f), 5′-AGG GCCTCACTAAACCATCC-3′ (r). The expression of candidate genes was normalized to reference genes GAPDH or 18S rRNA. The mRNA expression was analyzed by standard curve quantification. All measurements were performed in duplicates. Controls consisting of bidistilled H_2_O and RT^−^ controls were negative in all runs.

### Immunohistochemistry

Protein expression of renal TNFR1 and TNFR2 was detected on 4 µm cryostat sections of OCT-embedded tissue stained with polyclonal goat anti-mouse TNFR1 and polyclonal goat anti-mouse TNFR2 antibodies (both from R&D Systems, Wiesbaden, Germany). Specificity of the antisera was shown by negative staining of tissue sections from *Tnfr1*- and *Tnfr2*-deficient mice, respectively.

### Detection of TNF Receptor Surface Expression by Flow Cytometry

To detect TNFR1 surface expression glomerular endothelial or mesangial cells were incubated in fluorescence-activated cell sorting (FACS) buffer (PBS, 0.2% bovine serum albumin, 0.1% NaN_2_) with a monoclonal hamster anti-mouse TNFR1 antibody (clone 55R-286; BD Biosciences), followed by a biotin-labeled mouse anti-hamster IgG cocktail (BD Biosciences). Cells were then stained with phycoerythrin (PE)-conjugated streptavidin (BD Biosciences). Hamster IgG1 was used as the appropriate isotype control. TNFR2 expression was analyzed with a PE-conjugated monoclonal hamster anti-mouse TNFR2 antibody (clone TR75-89; BD Bioscience). PE-conjugated hamster IgG1 was used as isotype control.

### Chemokine ELISA

Chemokine levels in glomerular culture supernatants were determined using commercially available ELISA kits for CCL2/MCP-1, CCL5/RANTES and CXCL10/IP-10 (R&D Systems) following the manufacturer’s protocols.

### Quantification of Glomerular Leukocyte Subsets by Four-color Flow Cytometry

Single-cell suspensions from glomerular tissue preparations of individual kidneys were prepared as previously described [31]. Cells were incubated in FACS buffer on ice with combinations of fluorochrome-labeled antibodies to murine leukocyte surface markers including PE-conjugated anti-CD45 (clone 30-F11), FITC-conjugated Ly-6G (clone 1A8), FITC-conjugated CD3ε (clone 145-2C11), allophycocyanin (APC)-conjugated CD4 (clone RM4-5), PE-Cy5-conjugated CD8α (clone 53-6.7) (all from BD Biosciences), and APC-conjugated F4/80 (clone CL:A3-1; from AbD serotec, Oxford, UK). Stained cells were analysed with a FACS Calibur™ flow cytometer and Cellquest™ software (BD Biosciences). The number of positively stained cells was expressed as percentage of total glomerular cells.

### Statistical Analysis

Numerical results of each experimental group were expressed as mean±SD or SE as indicated, and were compared using two-sided non-parametric Mann-Whitney *U* test. When more than 2 experimental groups were compared, Kruskal-Wallis test was carried out, followed by pairwise Mann-Whitney U tests. Significance was assigned to P<0.05.

## Results

### Expression of TNFR1 and TNFR2 in Mouse Kidney and Intrinsic Glomerular Cells

Glomeruli were isolated from 6 to 8 week old wildtype mice after perfusion with paramagnetic Dynabeads (Figure S1A). The first supernatant obtained during the washing procedure consisted of a tubulointerstitial tissue fraction that was free of glomeruli (Figure S1B). To assure the glomerular and tubulointerstitial origin of the two tissue fractions mRNA expression of glomerular and tubular marker genes was determined examining nephrin and FXYD2, the γ-subunit of the tubular Na,K-ATPase, respectively (Figure S1C, S1D).

**Figure 1:**
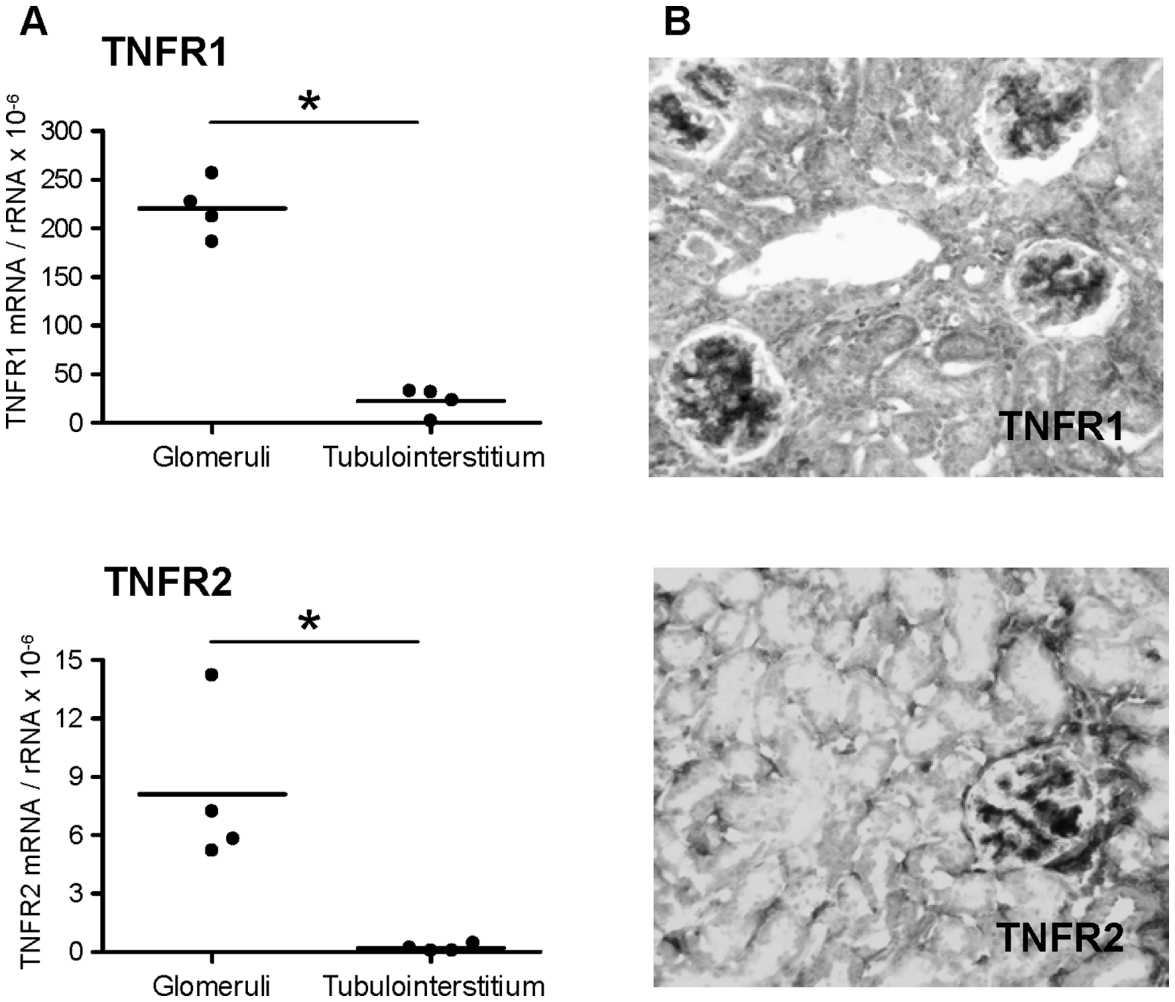
Expression of TNFR1 and TNFR2 in mouse kidney. **A:** Compartment-specific expression of TNFR1 and TNFR2 mRNA in glomeruli and tubulointerstitial tissue isolated from normal mouse kidney as analyzed by quantitative real-time PCR. Values were normalized to rRNA expression used as reference gene. ∗ p<0.05. **B:** Immunohistochemistry on frozen sections for TNFR1 and TNFR2 protein demonstrates a prominent glomerular expression of both receptors (black color product) in normal mouse kidney. Original magnification x200.

Compartment-specific quantitative PCR revealed a constitutive expression of TNFR1 and TNFR2 in normal mouse glomeruli, with substantially lower transcript levels in tubulointerstitial tissue (Figure 1A). Consistently, immunohistochemistry demonstrated a prominent protein expression of TNFR1 and TNFR2 in glomeruli, but only weak expression in the tubulointerstitium of normal mice (Figure 1B).

**Figure 2:**
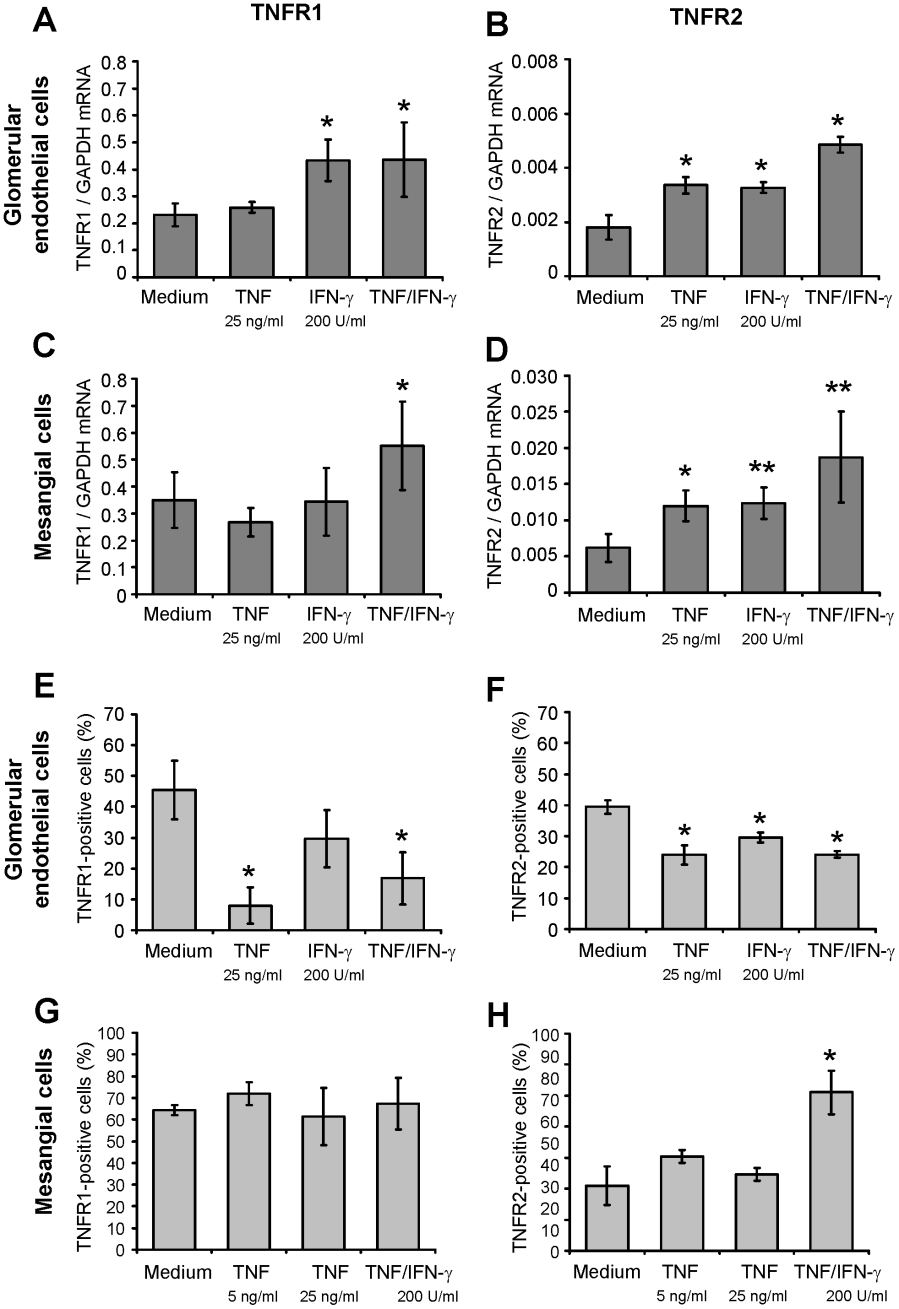
Expression of TNFR1 and TNFR2 in glomerular endothelial and mesangial cells in vitro. **A:** Expression of TNFR1 mRNA in unstimulated, TNF-stimulated and IFN-γ-stimulated glomerular endothelial cells. Cells were stimulated with indicated concentrations of TNF and/or IFN-γ for 5 hours. **B:** Expression of TNFR2 mRNA in unstimulated, TNF-stimulated and IFN-γ-stimulated glomerular endothelial cells. Cells were stimulated as in A. **C:** Expression of TNFR1 mRNA in unstimulated, TNF-stimulated and IFN-γ-stimulated mesangial cells. Cells were stimulated with indicated concentrations of TNF and/or IFN-γ for 24 hours. **D:** Expression of TNFR2 mRNA in unstimulated, TNF-stimulated and IFN-γ-stimulated glomerular endothelial cells. Cells were stimulated as in C. mRNA expression levels were analyzed by quantitative real-time PCR. Values were normalized to GAPDH expression used as reference gene. ∗ p<0.05, ∗∗ <0.01 (**A-D**). **E:** TNFR1 surface expression in unstimulated, TNF-stimulated and IFN-γ-stimulated glomerular endothelial cells in vitro analyzed by flow cytometry. Cells were stimulated with indicated concentrations of TNF and/or IFN-γ for 5 hours. **F:** TNFR2 surface expression in unstimulated, TNF-stimulated and IFN-γ-stimulated glomerular endothelial cells. Cells were stimulated as in E. **G:** Flow cytometric analysis of TNFR1 surface expression in unstimulated, TNF-stimulated and IFN-γ-stimulated mesangial cells in vitro. Cells were treated with indicated concentrations of TNF and IFN-γ for 24 hours. **H:** TNFR2 surface expression in unstimulated, TNF-stimulated and IFN-γ-stimulated mesangial cells. Cells were stimulated as in G. The percentage of TNFR-positive cells was determined in comparison to the respective isotype controls. ∗ p<0.05 versus medium control (**E-H**). Data are mean and SD of 3 to 6 independent experiments per group.

In vitro, glomerular endothelial (Figure 2A, 2B) and mesangial cells (Figure 2C, 2D) constitutively expressed mRNA of both TNFRs, with a substantially lower abundance of TNFR2 mRNA. After stimulation of both cell types with TNF and IFN-γ, TNFR1 mRNA was only upregulated after challenge with IFN-γ or TNF in combination with IFN-γ (Figure 2A, 2C). In contrast, TNFR2 expression was readibly inducable after incubation with TNF, IFN-γ or combined stimulation (Figure 2B, 2D). As TNF receptors are shed from the cell surface following ligand binding and activation we next analyzed surface expression of TNFR1 and TNFR2. In glomerular endothelial cells cytokine stimulation decreased surface expression of both TNFRs (Figure 2E, 2F). In contrast, TNFR1 and TNFR2 were not shed from the surface of mesangial cells upon stimulation, and surface expression of TNFR2 increased after combined stimulation with TNF and IFN-γ (Figure 2G, 2H), suggesting a robust mesangial TNFR signaling capacity in inflammatory conditions.

Together, these data demonstrate a constitutive glomerular expression of both TNFRs in normal mouse kidneys, which can be induced by proinflammatory stimuli, with TNFR2 being more readily upregulated than TNFR1.

### TNF-induced Expression of Glomerular Adhesion Molecules and Chemokines Correlates with Glomerular Leukocyte Infiltration in vivo

**Figure 3:**
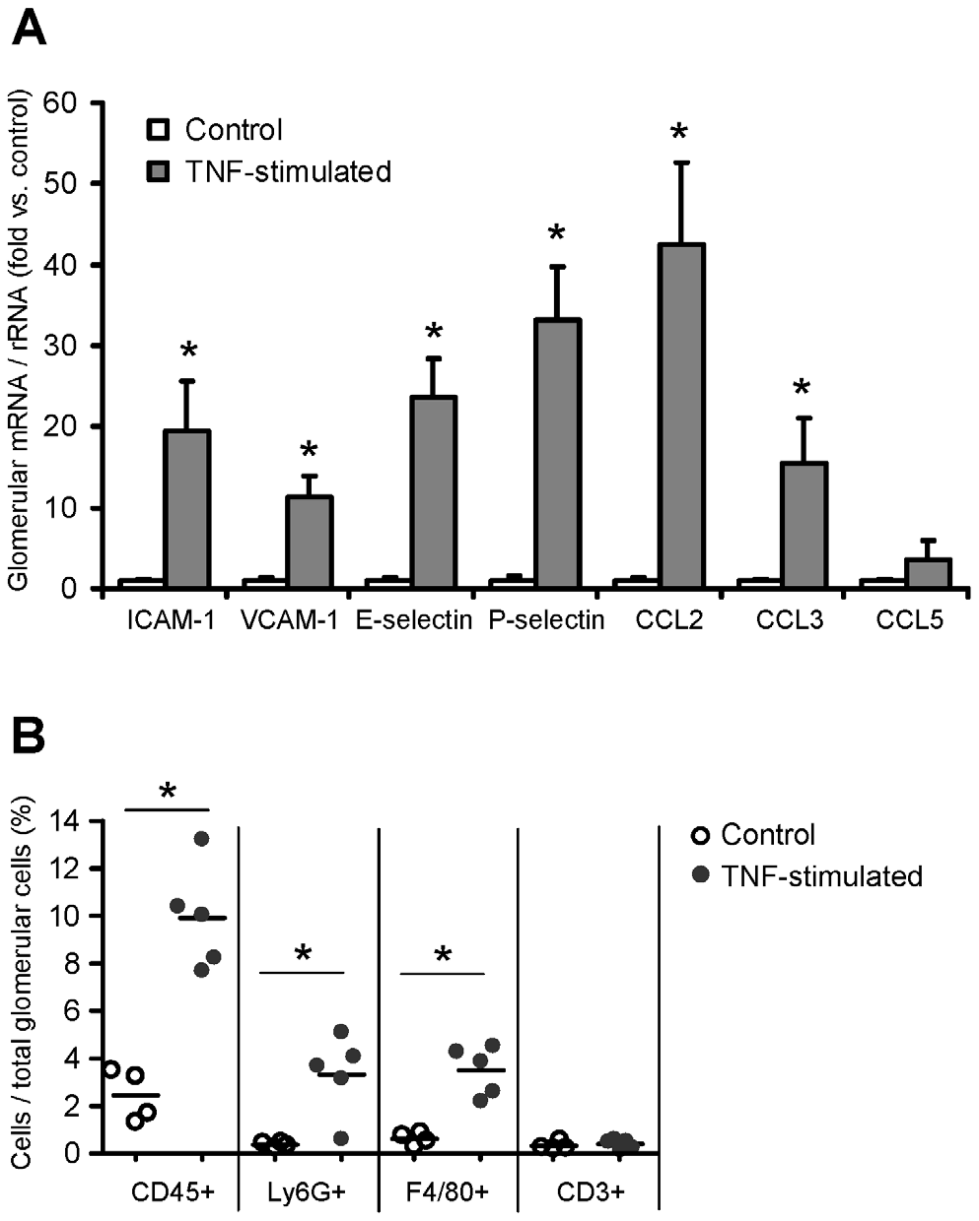
TNF-induced expression of glomerular adhesion molecules and chemokines correlates with leukocyte infiltration in vivo. **A:** Glomerular expression of adhesion molecules and proinflammatory chemokines in glomeruli isolated from control mice (white bars) and from mice 8 hours after intraperitoneal injection of 5 µg TNF (grey bars). mRNA levels were determined by quantitative real-time PCR, and values were normalized to 18S rRNA as reference gene. Results are expressed as fold change compared to control glomeruli. Data shown are means and SE of n = 3 to 6 per group; ∗ p<0.05 versus control. **B:** Glomerular leukocyte infiltration 8 hours after intraperitoneal injection of TNF (5 µg) was analysed by compartment-specific flow cytometry. Leukocyte subpopulations were identified as follows: leukocytes: CD45^+^, neutrophils: CD45^+^ Ly6G^+^ F4/80^−^, F4/80 positive mononuclear phagocytes: CD45^+^ F4/80^+^ Ly6G^−^, and T cells: CD45^+^ CD3ε^+^. Leukocyte numbers are expressed as percentage of all glomerular cells. ∗ p<0.05 versus control.

8 hours after intraperitoneal injection of 5 µg TNF, glomerular mRNA expression of adhesion molecules and chemokines substantially increased, with the highest induction (42.5-fold) seen for the proinflammatory chemokine CCL2/MCP-1 (Figure 3A). This was associated with glomerular influx of CD45^+^ leukocytes, mainly Ly6C^+^ neutrophils and F4/80^+^ mononuclear phagocytes, as revealed by compartment-specific flow cytometry (Figure 3B). These data demonstrate that TNF-induced expression of glomerular adhesion molecules and chemokines results in a rapid glomerular influx of leukocytes, which may substantially contribute to the glomerular production of inflammatory mediators. Thus, analysis of TNF-exposed glomeruli in vivo would not allow the characterization of local TNFR-mediated inflammatory responses in intrinsic glomerular cells as opposed to infiltrating leukocytes.

### Analysis of TNFR1- and TNFR2-specific Inflammatory Responses in TNF-stimulated Glomeruli ex vivo by Microarray Expression Profiling

To examine TNFR-specific inflammatory responses in intrinsic glomerular cells but not infiltrating leukocytes we stimulated intact glomeruli isolated from wildtype and *Tnfr*-deficient mice with soluble TNF ex vivo. Global gene expression levels were determined on Affymetrix Mouse Genome 430 2.0 gene chips. Representative genes differentially expressed in TNF-stimulated *Tnfr1,2*−/−, *Tnfr1*−/− and *Tnfr2*−/− glomeruli compared to wildtype are listed in Table S1. A comprehensive summary of all differentially expressed gene sets and genes in *Tnfr1,2*−/−, *Tnfr1*−/−, and *Tnfr2*−/− glomeruli compared to wildtype is shown in Table S2, S3, S4).

**Figure 4:**
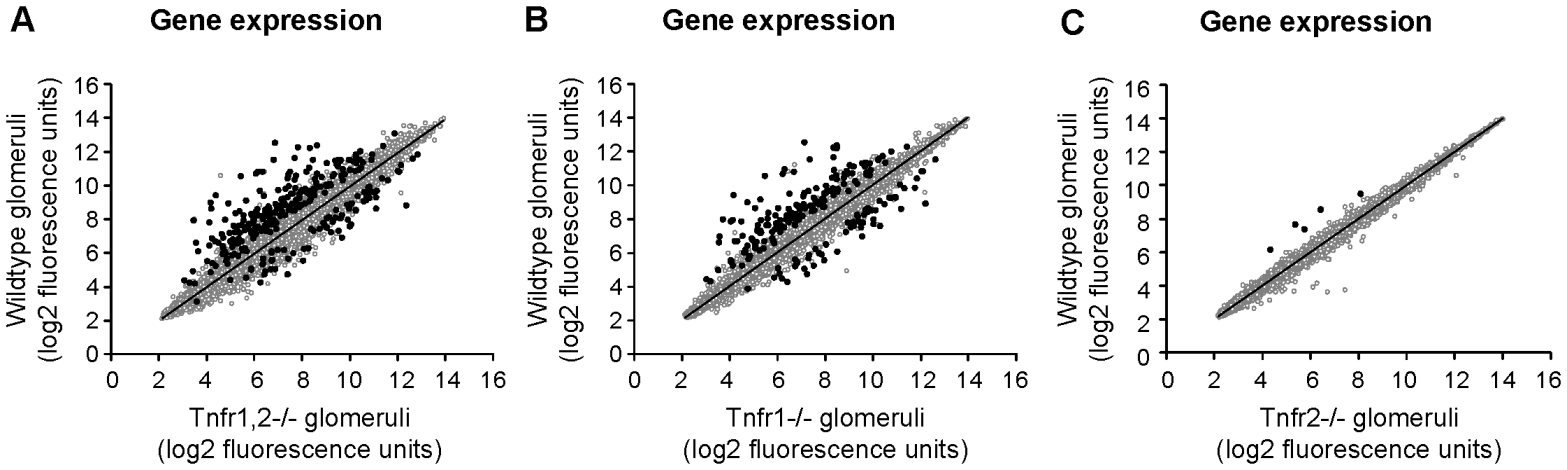
Differentially expressed genes in *Tnfr*-deficient glomeruli compared to wildtype after TNF stimulation. Expression profiling by microarrays was performed on isolated glomeruli from wildtype, *Tnfr1,2*−/−, *Tnfr1*−/− and *Tnfr2*−/− mice after stimulation with 50 ng/ml TNF for 12 hours ex vivo. **A:** Significance analysis using SAM detected differential regulation of 290 unique genes in *Tnfr1,2*−/− glomeruli with at least 1.5-fold difference compared to wildtype (black data points). 219 genes were down-regulated (upper left), and 71 genes were up-regulated (lower right). **B:** In *Tnfr1*−/− mice 219 differentially regulated genes were identified, with lower expression of 159 genes and increased expression of 60 genes. **C:** In *Tnfr2*-deficient glomeruli only 5 genes were differentially expressed, all of them with decreased expression compared to wildtype. In all three genotypes, expression of the majority of genes with fluorescence signals above background level did not significantly differ from wildtype (grey data points). Data represent the mean log2 value of fluorescence signals from three independent experiments per genotype.

**Figure 5:**
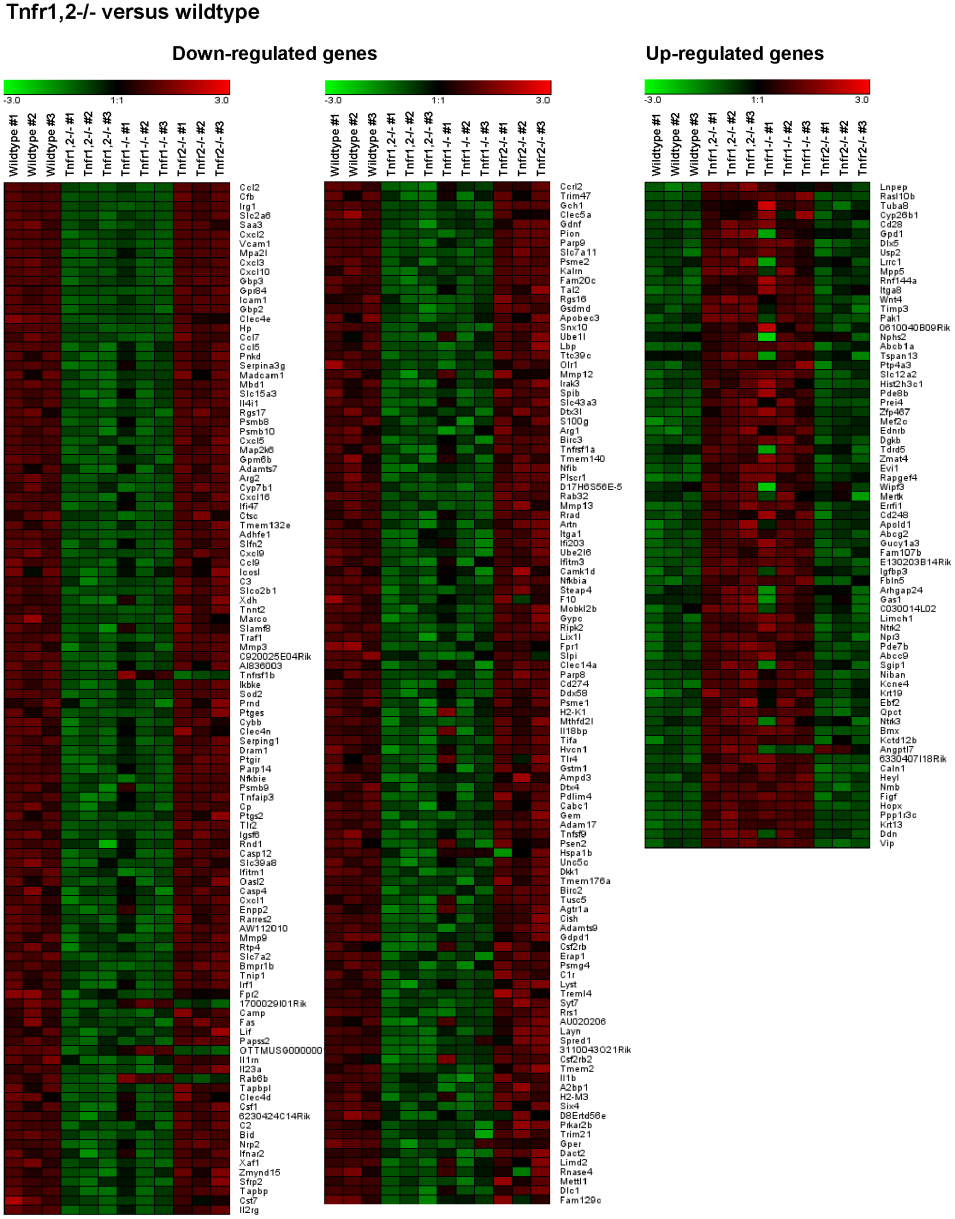
Gene expression profiles in wildtype and *Tnfr1,2-*deficient glomeruli after TNF stimulation ex vivo. Expression profiling by microarrays was performed on isolated glomeruli from wildtype and *Tnfr1,2*−/− mice 12 after stimulation with 50 ng/ml TNF for 12 hours ex vivo. A negativ “fold change” (black to green) indicates decreased expression and a postive “fold change” (black to red) indicates increased expression in a glomerular sample compared with the average of all analyzed samples. The 290 differentially regulated genes identified in *Tnfr1,2*−/− glomeruli are listed according to fold-change values versus wildtype, starting with the most down-regulated gene (CCL2; -50.0-fold). Note that almost all genes found significantly regulated in *Tnfr1,2*−/− glomeruli had a similar trend of expression change in *Tnfr1*−/− glomeruli, although not reaching statistical significance in *Tnfr1*−/− glomeruli for several genes. Table S2 lists all differentially expressed genes with respective probe set IDs, gene bank accession numbers, gene names and fold-changes according to fold-change values compared to wildtype.

**Figure 6:**
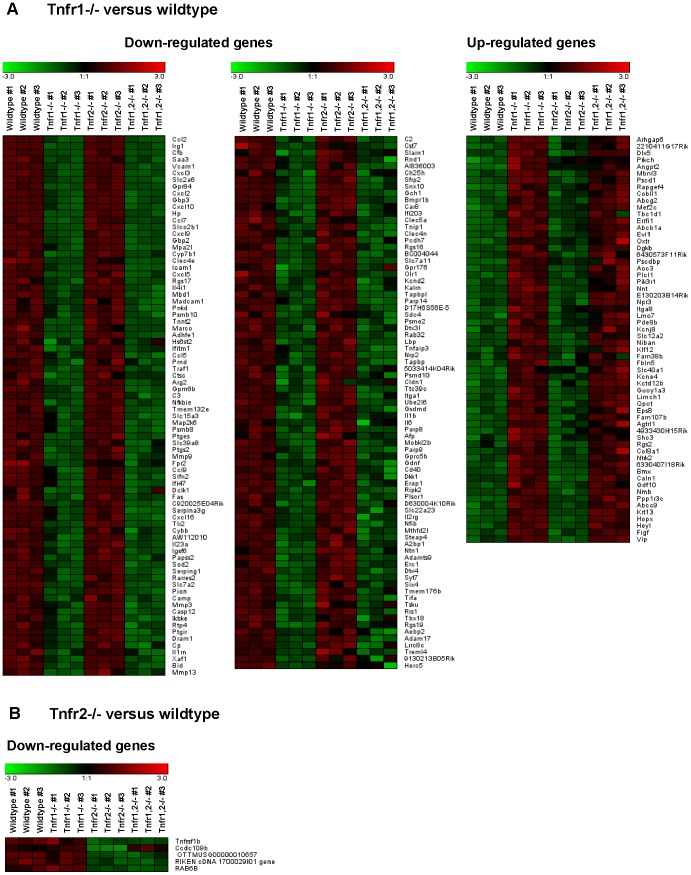
Gene expression profiles in wildtype, *Tnfr1*- and *Tnfr2-*deficient glomeruli after TNF stimulation ex vivo. Expression profiling by microarrays was performed on isolated glomeruli from wildtype, *Tnfr1*−/− and *Tnfr2*−/− mice 12 after stimulation with 50 ng/ml TNF for 12 hours ex vivo. A negativ “fold change” (black to green) indicates decreased expression and a postive “fold change” (black to red) indicates increased expression in a glomerular sample compared with the average of all analyzed samples. **A**: Compared to wildtype 219 significantly regulated genes were found in *Tnfr1*−/− glomeruli. The number of genes identified in *Tnfr1*−/− glomeruli was lower than in *Tnfr1,2*−/− glomeruli, but all genes differentially expressed in *Tnfr1*−/− glomeruli were regulated similarly in *Tnfr1,2*−/− glomeruli, although for some genes pair-wise comparison between *Tnfr1,2*−/− to wildtype did not reach significance or was below the pre-defined 1.5-fold cut-off (see also Table S3). **B**: 5 differentially expressed genes in *Tnfr2*−/− glomeruli are listed according to fold-change values compared to wildtype. These genes are not differentially expressed in *Tnfr1*−/− glomeruli, but 4 of the 5 genes are similarly regulated in *Tnfr1,2−/−* mice. Tables S3 (*Tnfr1*−/−) and S4 (*Tnfr2*−/−) list all differentially expressed genes with respective probe set IDs, gene bank accession numbers, gene names and fold-changes according to fold-change values compared to wildtype.

In *Tnfr1,2*−/− glomeruli, i.e. glomeruli without TNF-signaling capacity, we detected 290 differentially expressed genes compared to wildtype glomeruli following TNF stimulaton, with 219 genes being down-regulated, and 71 genes with up-reglulated expression (Figure 4A, 5, Table S2). Among these, 168 genes were regulated similarly (i.e. up- or down-regulated) as in *Tnfr1*−/− glomeruli (Figure 4B, 6A, Table S3), without altered expression in *Tnfr2*−/− glomeruli. Expression of 5 genes was decreased in *Tnfr1,2*−/− as well as *Tnfr2*−/− glomeruli (Figure 4C, 6B, Table S4), but not in *Tnfr1*−/−. The remaining 117 genes were differentially expressed in *Tnfr1,2*−/− glomeruli only. However, as shown in Figure 5 all of these genes had a trend towards similar regulation in *Tnfr1*−/− glomeruli, with mean fold changes of expression levels mostly >1.5, although not reaching statistical significance. In contrast, these genes were not differentially expressed in *Tnfr2*−/− glomeruli compared to wildtype (Figure 5). Functional groups of genes expressed in a TNFR1,2-dependent way included cell adhesion proteins, proinflammatory chemokines, cytokines and cytokine receptors, innate immune effectors, antigen presentation proteins, components of the NF-κB signaling cascade, matrix metalloproteinases (MMPs), transport proteins, and signalling molecules (see Table S1), indicating a robust proinflammatory stimulation of wildtype glomeruli by TNF.

Comparing TNF-stimulated wildtype and *Tnfr1*−/− glomeruli, we identified 219 unique genes being differentially expressed between the two genotypes (Figure 4B, 6A, Table S3). Of these, 159 genes were down-regulated in *Tnfr1*−/− glomeruli, and 60 were up-regulated. None of these genes was differentially expressed in *Tnfr2*−/− glomeruli following TNF-stimulation. Representative genes differentially expressed in *Tnfr1*−/− glomeruli are shown in Table S1. The proinflammatory chemokine CCL2/MCP-1 was the most prominently down-regulated gene (-42.9-fold) in *Tnfr1*−/− glomeruli. Of note, the magnitude of this differential expression was similar to our in vivo result showing a 42.5-fold induction of glomerular CCL2/MCP-1 mRNA after intraperitoneal TNF injection in wildtype mice (Figure 3A). TNFR1-dependently expressed genes included several cell adhesion molecules, proinflammatory chemokines of the CXC and CC subfamilies, and prototypic proinflammatory cytokines like pro-interleukin (IL)-1β and IL-6, all of which were down-regulated in *Tnfr1*−/− glomeruli. These data suggest a prominent role for TNFR1 in mediating glomerular leukocyte accumulation in TNF-stimulated glomeruli. Additionally, effectors and receptors of the innate immune system, including complement components, the lipopolysaccharide binding protein, toll-like receptor (TLR) 2, and proteolytic enzymes like MMP 3, 9, and 13 were down-regulated in *Tnfr1*-deficient glomeruli. Many TNFR1-induced proinflammatory responses are mediated through NF-κB signaling pathways, and expression of various components and regulators of the NF-κB signaling cascade was reduced in *Tnfr1*−/− glomeruli. Besides proinflammatory genes, several mediators of apoptosis (including Fas and caspase 12) were expressed TNFR1-dependently.

In contrast to *Tnfr1*−/−, only 5 genes were found to be differentially expressed in *Tnfr2*−/− glomeruli, all of which were down-regulated compared to wildtype (Figure 4C, Figure 6B, see also Table S1, Table S4). These 5 genes were not differentially expressed in *Tnfr*1−/− glomeruli, but expression of 4 of the 5 genes was also reduced in *Tnfr1,2*−/− glomeruli (Table S1). Among the 4 TNFR2-dependently expressed genes (excluding TNFR2) we identified a 2.6-fold reduced expression of the small GTPase Rab6B in *Tnfr2*−/− glomeruli (Table S1, Table S4).

In composite, the array data suggest a prominent role of TNFR1, in contrast to TNFR2, in mediating proinflammatory effects in glomeruli exposed to soluble TNF.

### Enriched Gene Groups and Gene Ontology Classes within TNFR-dependently Expressed Genes

To further characterize functional roles of TNFR-dependently expressed genes we analysed *Tnfr1,2*−/− and *Tnfr1*−/− regulated gene sets using DAVID and the EASE bioinformatic algorithms. Due to the few genes identified in *Tnfr2*−/− glomeruli, this analysis was not performed on the *Tnfr2*−/− specific gene set. The enriched functional gene groups differentially expressed in *Tnfr1,2*−/− glomeruli illustrate a robust TNF-mediated proinflammatory response in intrinsic glomerular cells (Table S5). Chemokines and cytokines constituted the most enriched group (enrichment score 8.86), with all members being downregulated in *Tnfr1,2*−/− glomeruli compared to wildtype. Applying the functional annotation chart feature of DAVID we identified 136 significantly enriched (i.e. overrepresented) gene ontology categories associated with the differentially regulated genes in *Tnfr1,2*−/− glomeruli. Many of the 10 most enriched gene ontology categories in *Tnfr1,2*−/− glomeruli were immune-related, including chemokine activity and chemokine receptor binding (Table 1).

**Table 1:** Overrepresented gene ontology terms within differentially expressed genes in TNF-stimulated *Tnfr1,2*−/− gomeruli 1.

GO term ID	GO term name	Fold enrichment	*P*-value	Gene count	% of regulated genes
GO:0008009	chemokine activity	19.7	1.29E-10	11.0	3.57
GO:0042379	chemokine receptor binding	19.2	1.70E-10	11.0	3.57
GO:0001664	G-protein-coupled receptor binding	12.0	2.26E-08	11.0	3.57
GO:0002474	antigen processing and presentation of peptideantigen via MHC class I	11.0	5.36E-03	4.0	1.30
GO:0000270	peptidoglycan metabolic process	9.5	8.31E-03	4.0	1.30
GO:0006958	complement activation, classical pathway	9.1	9.17E-03	4.0	1.30
GO:0042108	positive regulation of cytokine biosynthetic process	8.8	5.48E-04	6.0	1.95
GO:0008217	regulation of blood pressure	8.6	6.08E-04	6.0	1.95
GO:0050727	regulation of inflammatory response	8.6	1.10E-02	4.0	1.30
GO:0031347	regulation of defense response	8.6	1.10E-02	4.0	1.30

1 The 10 most overrepresented gene ontology (GO) terms are listed according to their enrichment scores. Terms with an EASE score of p ≤0.05, fold enrichment ≥1.5, and at least 4 genes per group were considered significant for overrepresentation.

Enriched gene groups in *Tnfr1*−/− glomeruli were similar to *Tnfr1,2*−/− (Table S6). Again, chemokines and cytokines were the group with the most significant differential expression (enrichment score 7.61). Compared to *Tnfr1,2*−/− glomeruli DAVID analysis identified fewer, but substantially overlapping enriched biological terms in *Tnfr1*−/− glomeruli, with 77 overrepresented categories being associated with the differentially regulated genes. Among the 10 most enriched gene ontology categories in *Tnfr1*−/− glomeruli (Table 2) chemokine activity, chemokine receptor binding, G-protein-coupled receptor binding, antigen processing and presentation, and regulation of blood pressure were identical to those found in *Tnfr1,2*−/− glomeruli. Additional highly enriched gene onotology categories were again immune-related, such as acute phase response, positive regulation of the I-κB kinase/NF-κB cascade, acute inflammatory response, and chemotaxis (Table 2).

**Table 2:** Overrepresented gene ontology terms within differentially expressed genes in TNF-stimulated *Tnfr1*−/− glomeruli 1.

GO term ID	GO term name	Fold enrichment	*P*-value	Gene count	% of regulated genes
GO:0008009	chemokine activity	22.8	3.54E-10	10	4.26
GO:0042379	chemokine receptor binding	22.3	4.53E-10	10	4.26
GO:0002474	antigen processing and presentation of peptideantigen via MHC class I	14.1	2.69E-03	4	1.70
GO:0001664	G-protein-coupled receptor binding	13.9	3.62E-08	10	4.26
GO:0006953	acute-phase response	12.5	3.79E-03	4	1.70
GO:0008217	regulation of blood pressure	9.2	2.05E-03	5	2.13
GO:0043123	positive regulation of I-κB kinase/NF-κB cascade	8.5	1.15E-02	4	1.70
GO:0002526	acute inflammatory response	8.3	4.97E-05	8	3.40
GO:0006935	chemotaxis	8.2	8.50E-07	11	4.68
GO:0042330	taxis	8.2	8.50E-07	11	4.68

1 The 10 most overrepresented gene ontology (GO) terms are listed according to their enrichment scores. Terms with an EASE score of p ≤0.05, fold enrichment ≥1.5, and at least 4 genes per group were considered significant for overrepresentation.

Taken together, these results suggest a predominant role of TNFR1 in mediating many aspects of soluble TNF-induced inflammation in the glomerulus. In the presence of TNFR1, TNFR2 may additionally contribute to TNF signaling in cooperation with TNFR1, as DAVID analysis identified more enriched gene ontology terms in *Tnfr1,2*−/− glomeruli compared to *Tnfr1*−/− glomeruli.

### Quantitative RT-PCR Analysis of Differentially Regulated Genes in TNF-stimulated Glomeruli Isolated from Wildtype and *Tnfr*-deficient Mice

**Figure 7:**
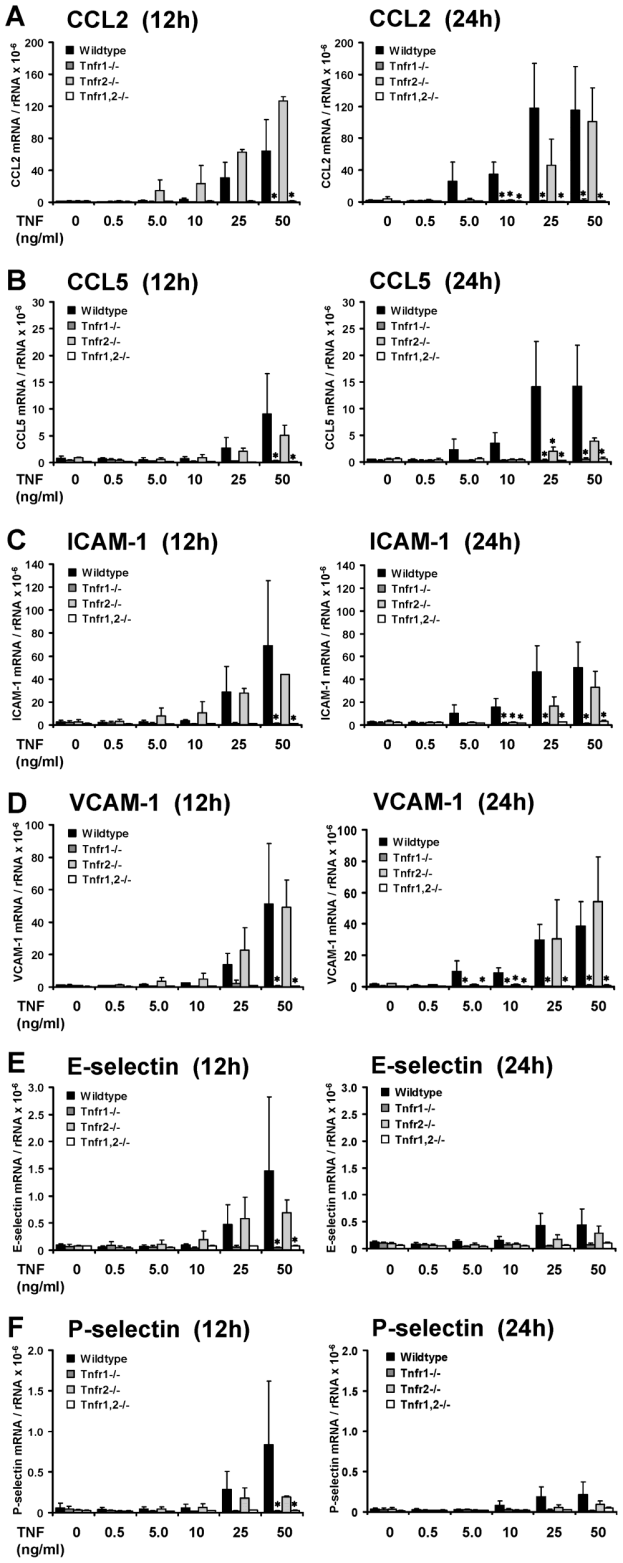
Validation of the array expression pattern by quantitative RT-PCR. Expression of proinflammatory chemokines and adhesion molecules in glomeruli isolated from control mice (black bars), *Tnfr1*−/− (dark grey bars), *Tnfr2*−/− (bright grey bars), and *Tnfr1,2*−/− mice (white bars) was anlayzed after challenge with indicated concentrations of TNF in vitro for 12 or 24 hours. The mRNA levels of CCL2/MCP-1 (**A**), CCL5/RANTES (**B**), ICAM-1 (**C**), VCAM-1 (**D**), E-selectin (**E**), and P-selectin (**F**) were determined by quantitative real-time PCR, and values were normalized to 18S rRNA as reference gene. Data shown are means and SE of n = 3 to 4 per group; ∗ p<0.05 versus wildtype.

Microarray expression data were validated by qRT-PCR for chemokines, adhesion molecules and Rab6B. CCL2/MCP-1 expression was induced in a dose-dependent fashion in TNF-stimulated wildtype glomeruli, and this induction was completely abrogated in *Tnfr1*−/− and *Tnfr1,2*−/− glomeruli at 12 and 24 hours (Figure 7A). In contrast, CCL2 expression was not significantly altered in *Tnfr2*−/− glomeruli at 12 hours compared to wildtype. However, at 24 hours CCL2 mRNA levels were significantly reduced after exposure of *Tnfr2*−/− glomeruli to lower TNF concentrations, i.e 10 ng/ml, with a similar trend for 25 ng/ml. TNF stimulation with 50 ng/ml resulted in a comparable induction of CCL2 mRNAs in *Tnfr2*−/− and wildtype glomeruli (Figure 7A). Similar results were found for CCL5/RANTES (Figure 7B) and the adhesion molecules ICAM-1 (Figure 7C) and VCAM-1 (Figure 7D). We also analyzed the expression pattern of E-selectin (Figure 7E) and P-selectin (Figure 7F) in TNF-stimulated glomeruli of all four genotypes. As shown before in vivo (Figure 3A), we detected an induced expression of both mRNA species in wildtype glomeruli in a dose dependent fashion at 12 hours, but not 24 hours. No induction occurred in *Tnfr1*−/− and *Tnfr1,2*−/− glomeruli. This differential expression may have been missed in the less sensitive microarray studies due to relative low expression levels. In summary, qRT-PCR confirmed the microarray data demonstrating a TNFR1-dependent expression of chemokines and adhesion molecules. Importantly, in the presence of TNFR1, TNFR2 additionally contributed to a prolonged expression of inflammatory mediators when glomeruli were exposed to low TNF concentrations.

**Figure 8:**
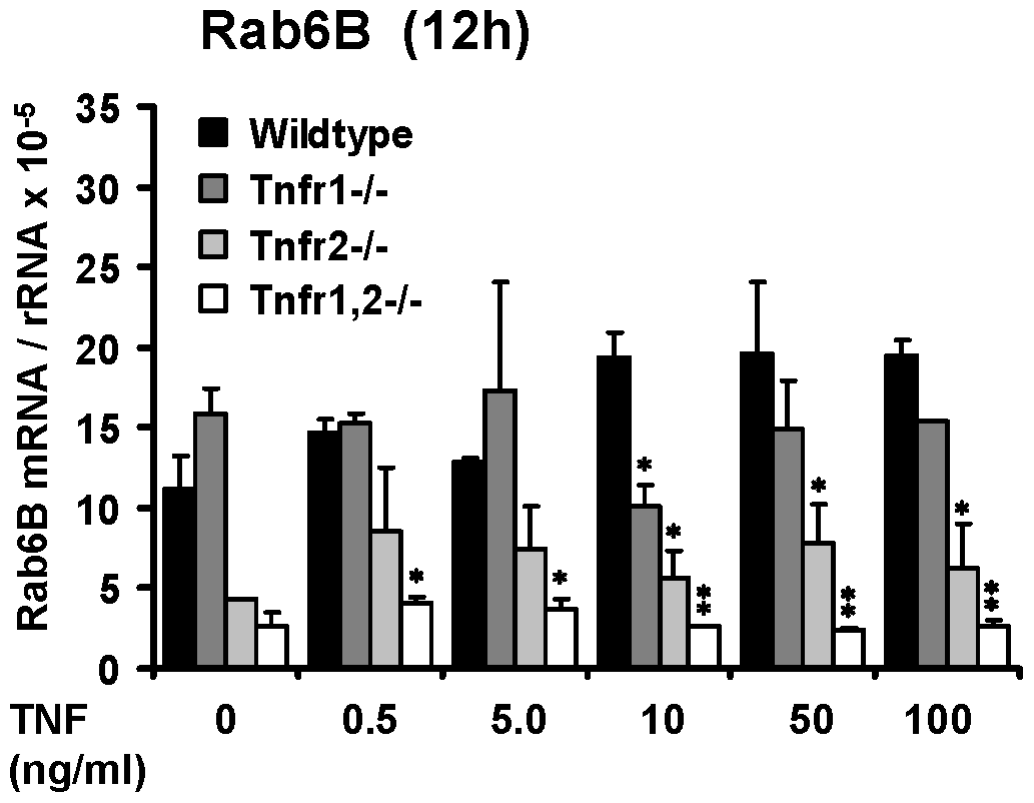
Glomerular expression of the small GTPase Rab6B. Rab6B expression was analyzed in glomeruli isolated from control mice (black bars), *Tnfr1*−/− (dark grey bars), *Tnfr2*−/− (bright grey bars), and *Tnfr1,2*−/− mice (white bars) after challenge with indicated concentrations of TNF in vitro for 12 or 24 hours. The mRNA levels of Rab6B were determined by quantitative real-time PCR, and values were normalized to 18S rRNA as reference gene. Data shown are means and SE of n = 3 per group; ∗ p<0.05, ∗∗ p<0.01 versus wildtype.

In contrast to the TNFR1-dependent induction of chemokines and adhesion molecules in TNF-stimulated glomeruli, expression of Rab6B was not substantially altered by TNF exposure in all four genotypes (Figure 8). However, in unstimulated *Tnfr2*−/− and *Tnfr1,2*−/−, but not *Tnfr1*−/− glomeruli we detected constitutively low expression levels of Rab6B. Compared to wildtype, glomerular expression of Rab6B remained significantly reduced after TNF stimulation of glomeruli lacking TNFR2 (Figure 8).

### TNF-induced Expression of Chemokines and Adhesion Molecules in Wildtype and *Tnfr*-deficient Primary Mesangial Cells

**Figure 9:**
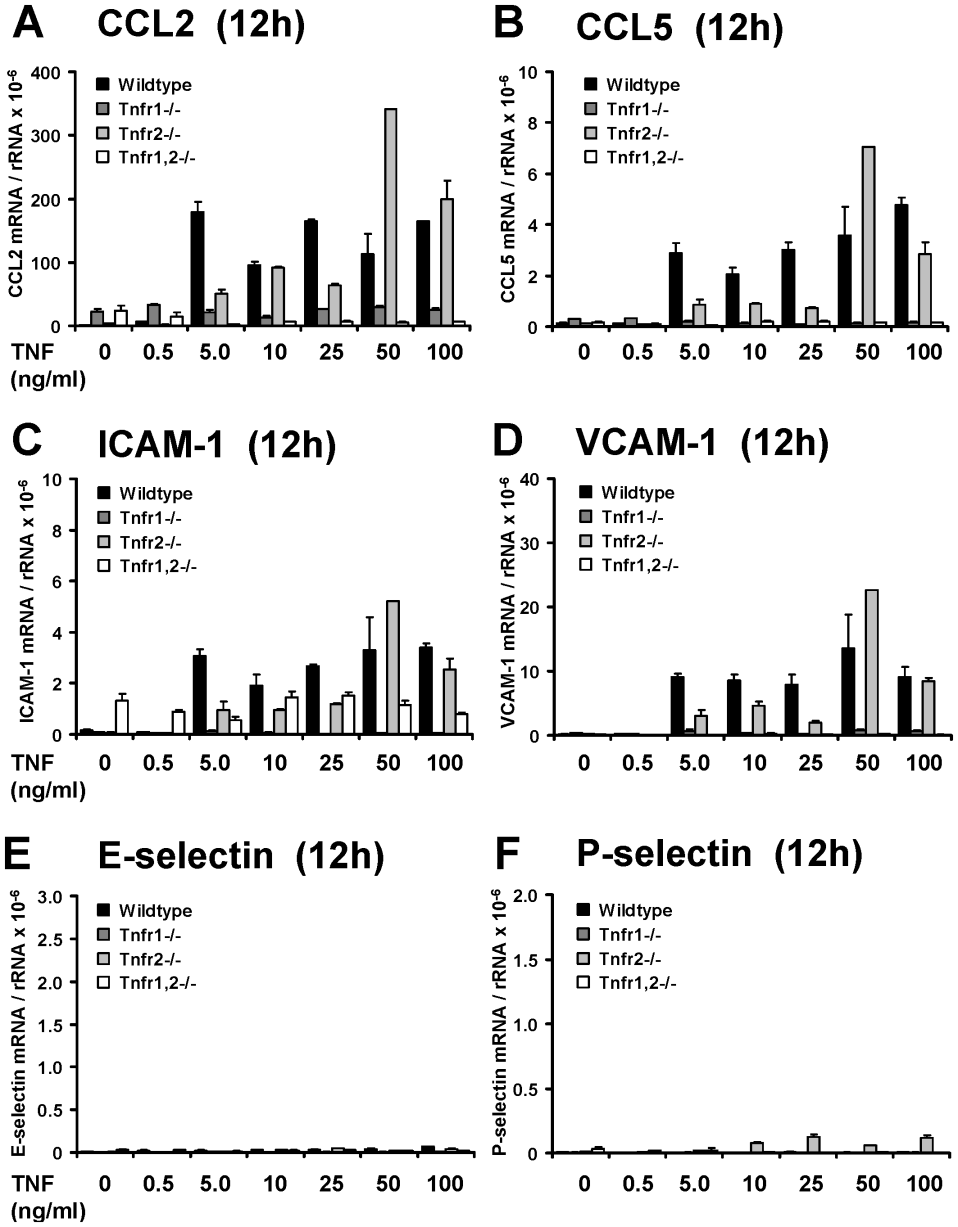
Expression of proinflammatory chemokines and adhesion molecules in TNF-stimulated primary mesangial cells (pMCs). pMCs were isolated from control mice (black bars), *Tnfr1*−/− (dark grey bars), *Tnfr2*−/− (bright grey bars), and *Tnfr1,2*−/− mice (white bars) after stimulation with indicated concentrations of TNF in vitro for 12 hours. The mRNA levels of CCL2/MCP-1 (**A**), CCL5/RANTES (**B**), ICAM-1 (**C**), VCAM-1 (**D**), E-selectin (**E**), and P-selectin (**F**) were determined by quantitative real-time PCR, and values were normalized to 18S rRNA as reference gene. Data shown are means and SE of two independently performed experiments.

Resident mesangial cells may substantially contribute to TNF-induced inflammatory responses in glomeruli. We therefore analyzed TNF-stimulated primary mesangial cells (pMCs) isolated from wildtype and *Tnfr*-deficient glomeruli. Responses of pMCs to TNF challenge were similar to isolated intact glomeruli. *Tnfr1*- or *Tnfr1,2*-deficiency, but not *Tnfr2-*deficiency prevented TNF-induced expression of chemokines and adhesion molecules (Figure 9A-D). We could not detect a significant expression of E- and P-selectin in unstimulated or TNF-stimulated pMCs (Figure 9E, 9F), although expression of both selectins was induced in TNF-treated intact glomeruli in vivo and in vitro (Figure 3A, Figure 7E, 7F). These data indicate that mesangial cells significantly contribute to TNF-induced expression of inflammatory mediators in the glomerulus, although some TNF effects like up-regulation of selectins are apparently not mediated by mesangial cells but other glomerular cell types.

### Reduced Secretion of Chemokines in TNF-stimulated Glomeruli from *Tnfr1−/−, Tnfr2−/−* and *Tnfr1,2−/−* Mice

**Figure 10:**
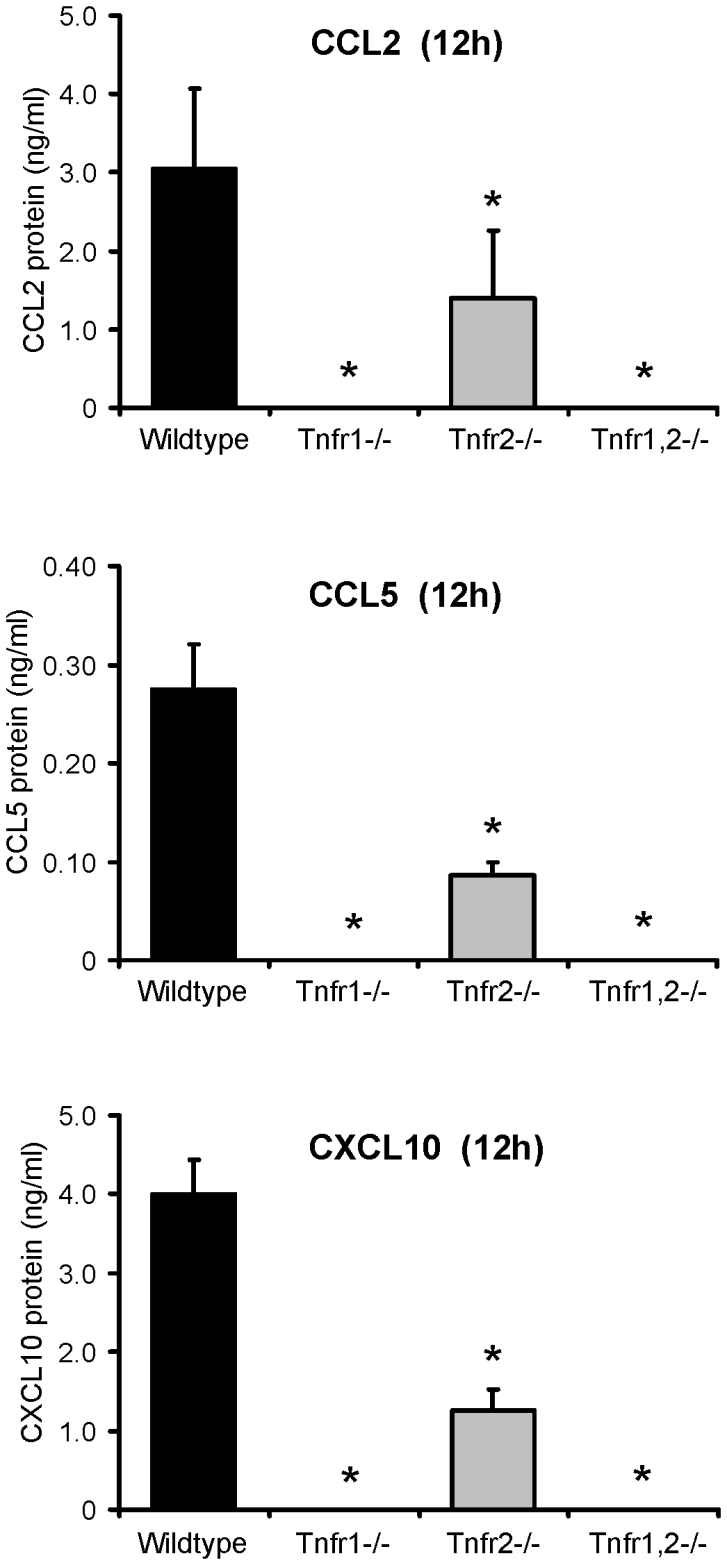
Secretion of proinflammatory chemokines by TNF-stimulated glomeruli ex vivo. Concentrations of CCL2/MCP-1, CCL5/RANTES, and CXCL10/IP-10 were measured by ELISA in culture supernatants of wildtype, *Tnfr1*−/−, *Tnfr2*−/−, and *Tnfr1,2*−/− glomeruli 12 hours after stimulation with 50 ng/ml of TNF. Data shown are means and SD of n = 3 per group; ∗ p<0.05 versus wildtype.

Consistent with the microarray and qRT-PCR data, TNF-induced secretion of CCL2/MCP-1, CCL5/RANTES, and CXCL10/IP-10 in wildtype glomeruli was completely abrogated in *Tnfr1*−/− and *Tnfr1,2*−/− glomeruli (Figure 10). Interestingly, TNF-induced chemokine secretion also significantly decreased in *Tnfr2*−/− glomeruli compared to wildtype (Figure 10), although mRNA expression of chemokines was not significantly different after stimulation with TNF under the same conditions (Figure 7, Table S1). Apparently, TNF-induced secretion of these chemokines is additionally dependent on TNFR2-mediated posttranscriptional modifications.

### Functional Roles of TNFR1 and TNFR2 in TNF-mediated Glomerular Inflammation in vivo

**Figure 11:**
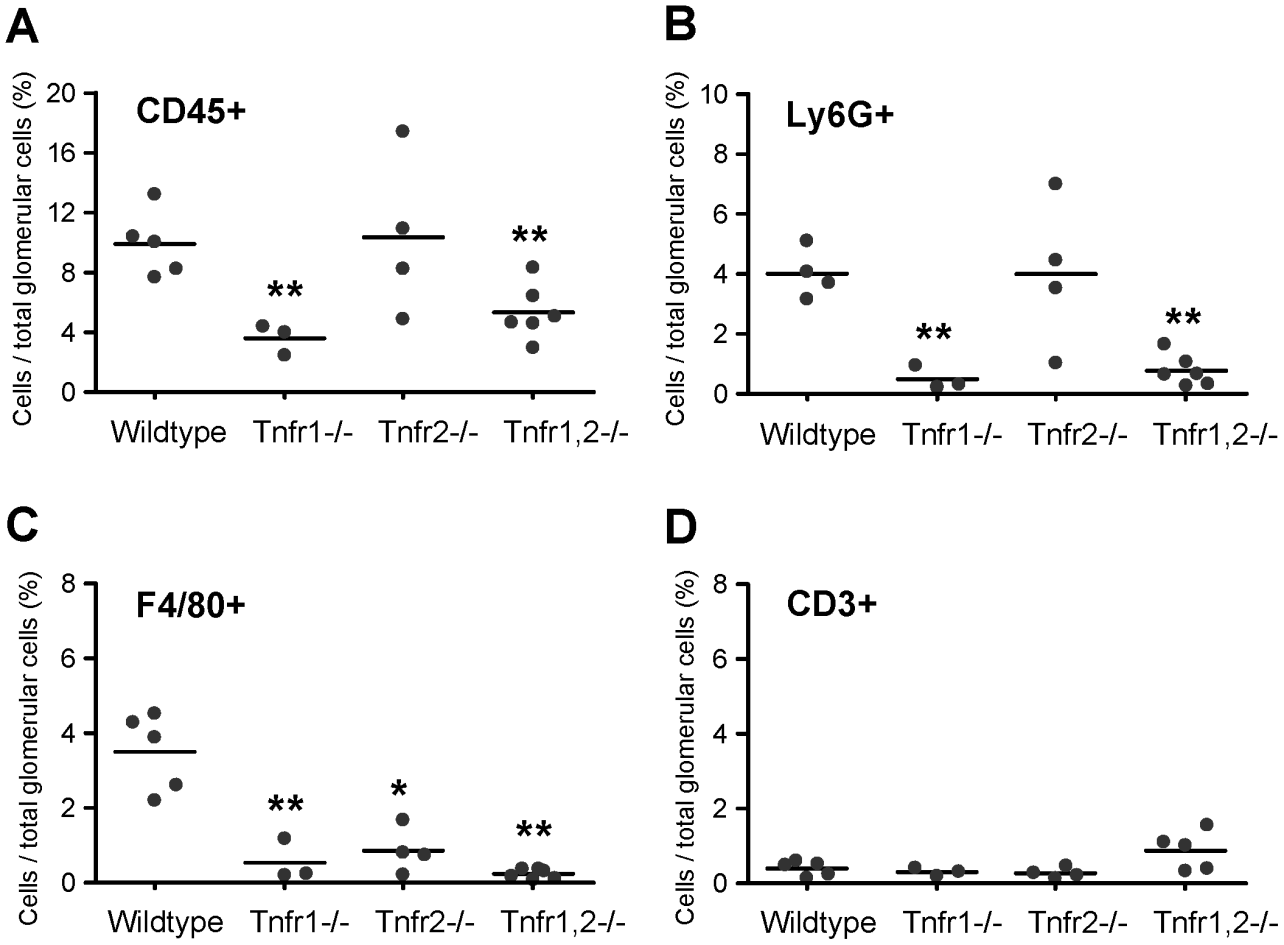
Contribution of TNFR1 and TNFR2 signaling to TNF-mediated infiltration of glomerular leukocytes. Compartment-specific flow cytometry was performed on glomerular tissue prepared from wildtype, *Tnfr1*−/−, *Tnfr2*−/−, and *Tnfr1,2*−/− mice 8 hours after intraperitoneal injection of 5 µg TNF. Leukocyte subpopulations were identified as follows: **A:** CD45^+^ leukocytes, **B:** CD45^+^ Ly6G^+^ F4/80^−^ neutrophils, **C:** CD45^+^ F4/80^+^ Ly6G^−^ mononuclear phagocytes, and **D:** CD45^+^ CD3ε^+^ T cells. Leukocyte numbers are expressed as percentage of all glomerular cells. ∗ p<0.05, ∗∗ p<0.01 versus wildtype.

Does the predominantly TNFR1-mediated inflammatory response in TNF-stimulated glomeruli in vitro translate into a functional role of TNFR1 in glomerular inflammation in vivo? To address this question we analyzed glomerular leukocyte infiltration in wildtype and *Tnfr*-deficient mice 8 hours after intraperitoneal TNF injection by compartment-specific flow cytometry. TNF-induced infiltrates of CD45^+^ leukocytes and Ly6G^+^ granulocytes were significantly reduced in *Tnfr1*−/− and *Tnfr1,2*−/− mice, but not in *Tnfr2*−/− mice (Figure 11A, 11B). Glomerular accumulation of F4/80^+^ mononuclear phagocytes decreased in *Tnfr1*−/−, *Tnfr1,2*−/− as well as *Tnfr2*−/− mice, indicating a non-redundant functional role of both TNFRs for glomerular phagocyte infiltration in TNF-challenged glomeruli (Figure 11C). Numbers of glomerular CD3^+^ T cells were not significantly different in all four genotypes (Figure 11D), consistent with absent T cell infiltrates (Figure 3B) within the eight hour time frame investigated. Together, these experiments reveal a predominant role of TNFR1 in the recruitment of glomerular leukocytes after exposure to soluble TNF in vivo, consistent with the identified in vitro effects of TNFR1 in TNF-challenged glomeruli. In contrast to neutrophils, TNFR2 contributed to the glomerular infiltration of F4/80^+^ leukocytes. This demonstrates an additional non-redundant role of TNFR2 specifically in the glomerular recruitment of mononuclear phagocytes, possibly due to its contribution to prolonged expression of inflammatory mediators when glomeruli are exposed to low TNF concentrations.

## Discussion

The functional role of TNF in glomerular inflammation has been shown by a variety of experimental studies [1], [2]. More recent data suggested distinct roles of the two TNF receptors in mediating glomerular inflammation, especially in the setting of immune complex GN [20] or collapsing glomerulopathies [32]. *Tnfr1*-deficiency delayed the onset of GN, but absent TNFR2 signaling in intrinsic renal cells protected from nephritis [20]. These studies demonstrate that resident glomerular cells differently respond to TNFR1 and TNFR2 activation during local, e.g. immune complex-mediated injury. To better understand underlying TNFR-specific inflammatory pathways in parenchymal glomerular cells that would explain the observed in vivo phenotypes in *Tnfr*-deficient mice, we analysed isolated intact glomeruli stimulated with soluble TNF ex vivo. This approach excluded additional changes of glomerular gene expression by leukocytes, which, as we show, readily accumulated in glomeruli following TNF exposure in vivo.

In comparison of wildtype and *Tnfr1,2*-deficient glomeruli without TNF signaling capacity, the results of our microarray experiments demonstrate that TNF induced expression of many innate immune effectors in intrinsic glomerular cells. All of these have been shown to be essential for glomerular leukocyte recruitment and activation, including adhesion molecules, chemokines and cytokines [33], [34], [35], [36], components of the complement system [37], [38], and metalloproteinases [39], [40]. Many inflammatory functions of TNF are induced via activation of the NF-κB signaling cascade. TNF treatment induced expression of several NF-κB transcription family members in wildtype glomeruli, as compared to *Tnfr1,2*−/− glomeruli, creating pathogenic feed-forward NF-κB loops in TNF-stimulated glomeruli.

When we compared TNF-induced gene expression between wildtype and *Tnfr1*-deficient glomeruli, differentially expressed genes and functional groups were similar to *Tnfr1,2*−/− glomeruli, indicating a predominant role of TNFR1 signaling in TNF-mediated glomerular effects. However, overall less genes were significantly regulated in *Tnfr1*−/− than *Tnfr1,2*−/− glomeruli, which suggests a contribution of TNFR2 to the inflammatory response in glomeruli when TNFR1 is present. This TNFR1-dependent synergistic effect of TNFR2 was also suggested by the qRT-PCR studies that examined expression profiles of selected chemokines and adhesion molecules in glomeruli challanged with increasing TNF concentrations up to 24 hours. In these experiments, low TNF concentrations did not induce mRNAs of these molecules in both *Tnfr1*−/− and *Tnfr2*−/− glomeruli as they did in wildtype glomeruli. In contrast, when glomeruli were stimulated with higher concentrations up to 50 ng/ml, expression of adhesion molecules and chemokines was induced in *Tnfr2*−/−, but not *Tnfr1*−/− glomeruli. Thus, in the presence of TNFR1, activation of TNFR2 may enhance proinflammatory TNF signaling when cells are stimulated with low concentrations of TNF for a prolonged time. This may result from the formation of receptor heterocomplexes [41] or a process referred to as ligand passing in which TNFR2-bound TNF increases the local TNF concentration in the vicinity of TNFR1 [42].

Surprisingly, our microarray analysis identified only 4 genes that were differentially regulated in *Tnfr2*−/− glomeruli compared to wildtype. These genes were not differentially expressed in *Tnfr1*-deficient glomeruli. The only well-characterized gene within this group was Rab6B, a small intracellular GTPase. Subsequent qRT-PCR analysis revealed that Rab6B expression was constitutively suppressed in *Tnfr2*−/− and *Tnfr1,2*−/−, but not *Tnfr1*−/− glomeruli, and was not induced after TNF stimulation. The reason for the TNFR2-dependent expression of Rab6B is not known, nor have functions of renal cell-expressed Rab6B been described. Proteins of the Rab family are important intracellular regulators of vesicular traffic in the secretory and endocytotic pathways [43], [44]. To this end, deficient Rab6B expression in *Tnfr2*−/− glomeruli may compromise the cellular capacity to secrete TNF-induced inflammatory mediators and contribute to reduced chemokine secretion, which we observed in TNF-stimulated *Tnfr2*−/− glomeruli, despite comparable mRNA expression levels with wildtype. Importantly, the latter finding identified a novel posttranscriptional role of glomerular TNFR2 which contributes to local chemokine production.

The lack of exclusively TNFR2-dependently expressed genes in our expression analysis was unexpected. Our data suggest that proinflammatory effects in parenchymal glomerular cells stimulated with soluble TNF are almost exclusively mediated via TNFR1 signaling, with only minor contributions of TNFR2 when TNFR1 is present. Our microarray data were confirmed by qRT-PCR analysis of TNF-stimulated glomeruli and mesangial cells, and importantly correlate in vivo with completely abrogated glomerular leukocyte infiltration in *Tnfr1-*, but not *Tnfr2*-deficient mice after intraperitoneal challenge with soluble TNF. In contrast, Bruggeman et al. recently reported a TNFR2-mediated NF-κB-dependent inflammatory response in immortalized podocytes stimulated with soluble TNF in vitro, without requirement for TNFR1 [32]. Although our data on isolated intact glomeruli do not rule out a contribution of podocyte-expressed TNFR2 to the proinflammatory TNFR2-dependent glomerular effects revealed in this study, we could not identify a TNFR1-independent function of TNFR2 in intact glomeruli ex vivo or TNF-stimulated mice in vivo. TNFR activation, however, may be different in podocytes located within intact glomeruli and immortalized podocytes cultures in vitro. Moreover, as all intrinsic glomerular cells (glomerular endothelial and mesangial cells as shown in this study, and podocytes [32]) can express TNFR1 and TNFR2, their relative contributions to TNF-induced glomerular injury remains to be elucidated analysing mice with cell type-specific deletion of these receptors.

Absence of the TNFR1-mediated proinflammatory effects identified in this study may well explain the delayed onset of GN in *Tnfr1*-deficient mice in vivo described previously [20]. As stimulation of intact glomeruli and mesangial cells with soluble TNF did not reveal any exclusively TNFR2-mediated inflammatory effects, potential mechanisms leading to protection from GN in *Tnfr2*-deficient mice [20] may involve activation of renal cell-expressed TNFR2 by membrane-bound TNF. This hypothesis is supported by findings that glomerular as opposed to systemic TNF mediates renal injury in GN, either expressed by activated adjacent glomerular cells [15], [16] or infiltrating macrophages [45]. Moreover, in contrast to soluble TNF which efficiently activates TNFR1 in vitro [46], membrane-bound TNF has been suggested to preferentially activate TNFR2 [47]. The high affinity of soluble TNF for TNFR1 apparently results from a marked stability of ligand receptor complexes, as opposed to transient interactions of soluble TNF with TNFR2 [46]. On the other hand, membrane-bound TNF was superior to soluble TNF in mediating TNFR2-dependent T cell activation, thymocyte proliferation, and granulocyte/macrophage colony-stimulating factor production [47]. Moreover, recent evidence indicates that TNFR2 activation induces degradation of TNF receptor-associated factor 2 (TRAF2), a key mediator of signal transduction of both TNFR1 and TNFR2 which is required for transcriptional activation of target genes [48]. When TNFR1 and TNFR2 were activated simultaneously, TNFR2-induced TRAF2 degradation and the subsequent decrease in NF-*κ*B activation resulted in an enhancement of cytotoxicity triggered by TNFR1 [49], [50]. Importantly, it was shown that TNFR2 is also able to trigger a cell death process that is independent of TNFR1 activation after upregulation of membrane-bound TNF and TNFR2, potentially involving necroptosis [50], [51]. In the context of GN these data suggest that glomerular activation of TNFR2 by membrane-bound TNF may trigger local cell injury which is essential for the induction of nephritis, as shown in the NTN model in vivo [20].

In summary, our glomerular expression analysis and in vivo studies identified TNFR1 as the major receptor mediating proinflammatory effects of soluble TNF in intrinsic glomerular cells. In the presence of TNFR1, TNFR2 contributes to this inflammatory response when glomeruli are stimulated with low TNF concentrations and promotes chemokine secretion. The potential contribution of membrane-bound TNF to TNFR2-dependent glomerular inflammation remains to be elucidated.

## Supporting Information

Figure S1 **Isolation of glomerular and tubulointerstitial tissue fractions from mouse kidneys. A:** Appearance of mouse glomeruli isolated after paramagnetic bead perfusion. **B:** The tubulointerstitial tissue fraction obtained during the first wash of the glomerular isolation procedure contained tubular fragments, tubular cells and polymorphic interstitial cells. Original magnification×100 (**A–B**). **C:** mRNA expression of the glomerular marker gene nephrin and **D:** FXYD2, the γ-subunit of the tubular Na,K-ATPase, in glomerular and tubulointerstitial tissue from wildtype mice as analyzed by quantitative real-time PCR. Values were normalized to rRNA expression used as reference gene. ∗ p<0.05.
(TIF)

Table S1 Representative genes differentially expressed in TNF-stimulated *Tnfr1,2*−/−, *Tnfr1*−/− and *Tnfr2*−/− glomeruli compared to wildtype as identified by microarray profiling.
(PDF)

Table S2 Differentially expressed genes in TNF-stimulated *Tnfr1,2*−/− glomeruli compared to wildtype as identified by microarray profiling.
(PDF)

Table S3 Differentially expressed genes in TNF-stimulated *Tnfr1−/−* glomeruli compared to wildtype as identified by microarray profiling.
(PDF)

Table S4 Differentially expressed genes in TNF-stimulated *Tnfr2*−/− glomeruli compared to wildtype as identified by microarray profiling.
(PDF)

Table S5 Enriched functional groups of differentially expressed genes in TNF-stimulated *Tnfr1,2*−/− glomeruli compared to wildtype as identified by DAVID.
(PDF)

Table S6 Enriched functional groups of differentially expressed genes in TNF-stimulated *Tnfr1*−/− glomeruli compared to wildtype as identified by DAVID.
(PDF)
