# Supplementary figures and images for: Distinct Contributions of TNF Receptor 1 and 2 to TNF-Induced Glomerular Inflammation in Mice
Source: PLoS One. 2013 Jul 15;8(7):e68167. doi: 10.1371/journal.pone.0068167 (PMC3711912; doi:10.1371/journal.pone.0068167)

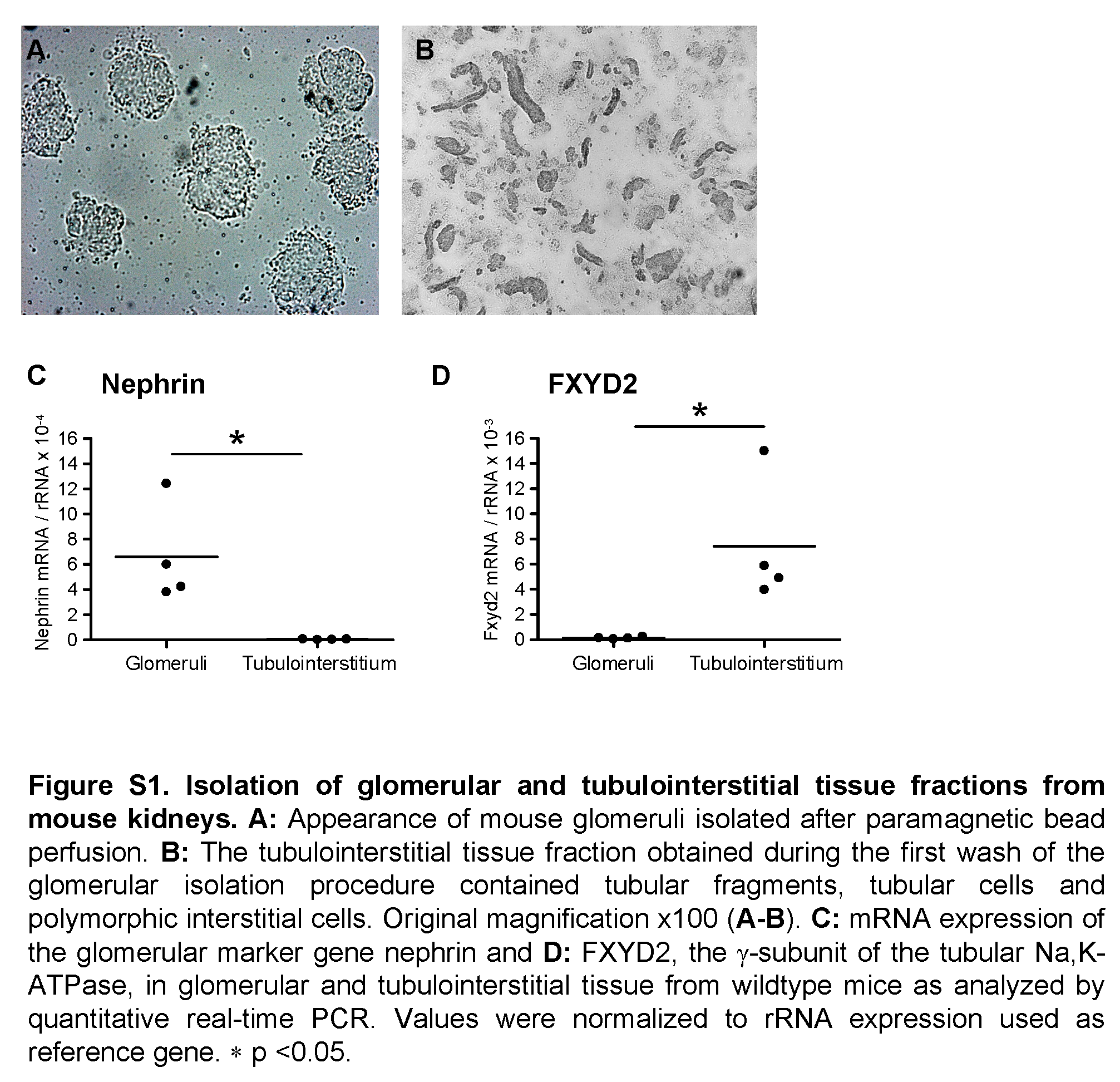

Supplement: Figure S1 — Isolation of glomerular and tubulointerstitial tissue fractions from mouse kidneys. A: Appearance of mouse glomeruli isolated after paramagnetic bead perfusion. B: The tubulointerstitial tissue fraction obtained during the first wash of the glomerular isolation procedure contained tubular fragments, tubular cells and polymorphic interstitial cells. Original magnification×100 (A–B). C: mRNA expression of the glomerular marker gene nephrin and D: FXYD2, the γ-subunit of the tubular Na,K-ATPase, in glomerular and tubulointerstitial tissue from wildtype mice as analyzed by quantitative real-time PCR. Values were normalized to rRNA expression used as reference gene. ∗ p<0.05. (TIF) [file pone.0068167.s001.tif]
