# Supplementary material for: Distinct Contributions of TNF Receptor 1 and 2 to TNF-Induced Glomerular Inflammation in Mice
Source: PLoS One. 2013 Jul 15;8(7):e68167. doi: 10.1371/journal.pone.0068167 (PMC3711912; doi:10.1371/journal.pone.0068167)
Supplement: Table S1 — Representative genes differentially expressed in TNF-stimulated Tnfr1,2−/−, Tnfr1−/− and Tnfr2−/− glomeruli compared to wildtype as identified by microarray profiling. (PDF) [file pone.0068167.s002.pdf]

Table S1. Representative genes differentially expressed in TNF-stimulated *Tnfr1,2*-/-, *Tnfr1*-/- and *Tnfr2*-/- glomeruli compared to wildtype (Wt) as identified by microarray profiling.

GeneBank ID	Gene symbol	Gene name	Fold-change versus Wt Tnfr1,2-/-	Tnfr1-/-	Tnfr2-/-
		Cell adhesion proteins			
NM_016674	Cldn1	claudin 1	n.s.	-2.4	n.s.
NM_007739	Col8a1	collagen, type VIII, alpha 1	n.s.	3.8	n.s.
NM_011812	Fbln5	fibulin 5	2.7	2.9	n.s.
NM_015819	Hs6st2	heparan sulfate 6-O-sulfotransferase 2	n.s.	-5.4	n.s.
NM_010493	Icam1	intercellular adhesion molecule 1	-15.4	-9.9	n.s.
NM_001033228	Itga1	integrin alpha 1	-2.3	-2.4	n.s.
NM_001001309	Itga8	integrin alpha 8	1.9	2.7	n.s.
NM_201529	Lmo7	LIM domain only 7	n.s.	2.7	n.s.
NM_013591	Madcam1	mucosal vascular addressin cell adhesion molecule 1	-9.1	-6.1	n.s.
NM_010939	Nrp2	neuropilin 2	-3.1	-2.4	n.s.
NM_018764	Pcdh7	protocadherin 7	n.s.	-2.7	n.s.
NM_011693	Vcam1	vascular cell adhesion molecule 1	-23.8	-22.1	n.s.
		Chemokines and chemokine receptors			
NM_011333	Ccl2	chemokine (C-C motif) ligand 2	-50.0	-42.9	n.s.
NM_013653	Ccl5	chemokine (C-C motif) ligand 5	-10.6	-5.2	n.s.
NM_013654	Ccl7	chemokine (C-C motif) ligand 7	-11.2	-14.7	n.s.
NM_011338	Ccl9	chemokine (C-C motif) ligand 9	-5.4	-3.9	n.s.
NM_017466	Ccrl2	chemokine (C-C motif) receptor-like 2	-2.9	n.s.	n.s.
NM_008176	Cxcl1	chemokine (C-X-C motif) ligand 1	-3.7	n.s.	n.s.
NM_009140	Cxcl2	chemokine (C-X-C motif) ligand 2	-24.9	-18.0	n.s.
NM_203320	Cxcl3	chemokine (C-X-C motif) ligand 3	-22.4	-19.4	n.s.
NM_009141	Cxcl5	chemokine (C-X-C motif) ligand 5	-7.8	-8.3	n.s.
NM_008599	Cxcl9	chemokine (C-X-C motif) ligand 9	-5.4	-14.0	n.s.
NM_021274	Cxcl10	chemokine (C-X-C motif) ligand 10	-21.0	-15.0	n.s.
NM_023158	Cxcl16	chemokine (C-X-C motif) ligand 16	-6.2	-3.6	n.s.

GeneBank ID	Gene symbol	Gene name	Fold-change versus Wt Tnfr1,2-/-	Tnfr1-/-	Tnfr2-/-
		Cytokines and cytokine receptors			
NM_011611	Cd40	CD40 antigen	n.s.	-2.1	n.s.
NM_007642	Cd28	CD28 antigen	1.8	n.s.	n.s.
NM_021893	Cd274	CD274 antigen	-2.1	n.s.	n.s.
NM_007778	Csf1	colony stimulating factor 1 (macrophage)	-3.2	n.s.	n.s.
NM_007780	Csf2rb	colony stimulating factor 2 receptor, beta, low-affinity (granulocyte-macrophage)	-1.8	n.s.	n.s.
NM_007781	Csf2rb2	colony stimulating factor 2 receptor, beta 2, low-affinity (granulocyte-macrophage)	-1.7	n.s.	n.s.
NM_015790	Icosl	icos ligand	-5.4	n.s.	n.s.
NM_010509	Ifnar2	interferon (alpha and beta) receptor 2	-3.1	n.s.	n.s.
NM_010531	Il18bp	interleukin 18 binding protein	-2.1	n.s.	n.s.
NM_008361	Il1b	interleukin 1 beta	-1.7	-2.3	n.s.
NM_031167	Il1rn	interleukin 1 receptor antagonist	-3.2	-3.0	n.s.
NM_013563	Il2rg	interleukin 2 receptor, gamma chain	-2.9	-2.0	n.s.
NM_031168	Il6	interleukin 6	n.s.	-2.3	n.s.
NM_031252	Il23a	interleukin 23, alpha subunit p19	-3.2	-3.5	n.s.
NM_008501	Lif	leukemia inhibitory factor	-3.2	n.s.	n.s.
NM_011609	Tnfrsf1a	tumor necrosis factor receptor superfamily, member 1a	-2.4	n.s.	n.s.
NM_011610	Tnfrsf1b	tumor necrosis factor receptor superfamily, member 1b	-4.4	n.s.	-4.9
NM_009404	Tnfsf9	tumor necrosis factor (ligand) superfamily, member 9	-1.9	n.s.	n.s.
		Innate immune effectors and receptors			
NM_023143	C1r	complement component 1, r sub-component	-1.8	n.s.	n.s.
NM_009778	C3	complement component 3	-5.2	-4.5	n.s.
NM_013484	C2	complement component 2 (within H-2S)	-3.1	-2.9	n.s.
NM_009921	Camp	cathelicidin antimicrobial peptide	-3.3	-3.2	n.s.
NM_008198	Cfb	complement factor B	-35.2	-34.0	n.s.
NM_010819	Clec4d	C-type lectin domain family 4, member d	-3.2	n.s.	n.s.
NM_019948	Clec4e	C-type lectin domain family 4, member e	-13.2	-10.2	n.s.
NM_020001	Clec4n	C-type lectin domain family 4, member n	-4.1	-2.7	n.s.
NM_021364	Clec5a	C-type lectin domain family 5, member a	-2.8	-2.7	n.s.
NM_172689	Ddx58	DEAD (Asp-Glu-Ala-Asp) box polypeptide 58	-2.1	n.s.	n.s.
NM_013521	Fpr1	formyl peptide receptor 1	-2.2	n.s.	n.s.
NM_008039	Fpr2	formyl peptide receptor 2	-3.4	-3.9	n.s.

GeneBank ID	Gene symbol	Gene name	Fold-change versus Wt Tnfr1,2-/-	Tnfr1-/-	Tnfr2-/-
NM_008489	Lbp	lipopolysaccharide binding protein	-2.5	-2.5	n.s.
NM_138648	Olr1	oxidized low density lipoprotein (lectin-like) receptor 1	-2.5	-2.6	n.s.
NM_011315	Saa3	serum amyloid A 3	-25.5	-24.7	n.s.
NM_009776	Serping1	serine (or cysteine) peptidase inhibitor, clade G, member 1	-4.1	-3.4	n.s.
NM_011905	Tlr2	toll-like receptor 2	-3.8	-3.6	n.s.
NM_021297	Tlr4	toll-like receptor 4	-2.0	n.s.	n.s.
		Antigen presentation proteins			
NM_019909	H2-K1	histocompatibility 2, K1, K region	-2.1	n.s.	n.s.
NM_013819	H2-M3	histocompatibility 2, M region locus 3	-1.7	n.s.	n.s.
NM_010724	Psmb8	proteasome (prosome, macropain) subunit, beta type 8 (large multifunctional peptidase 7)	-8.0	-4.3	n.s.
NM_013585	Psmb9	proteasome (prosome, macropain) subunit, beta type 9 (large multifunctional peptidase 2)	-3.9	n.s.	n.s.
NM_013640	Psmb10	proteasome (prosome, macropain) subunit, beta type 10	-7.9	-5.7	n.s.
NM_016883	Psmd10	proteasome (prosome, macropain) 26S subunit, non-ATPase, 10	n.s.	-2.4	n.s.
NM_011189	Psme1	proteasome (prosome, macropain) 28 subunit, alpha	-2.1	n.s.	n.s.
NM_011190	Psme2	proteasome (prosome, macropain) 28 subunit, beta	-2.7	-2.5	n.s.
NM_009318	Tapbp	TAP binding protein	-2.9	-2.4	n.s.
NM_145391	Tapbpl	TAP binding protein-like	-3.2	-2.5	n.s.
		NF-κB factors and regulators			
NM_019777	Ikbke	inhibitor of kappaB kinase epsilon	-4.2	-3.1	n.s.
NM_010907	Nfkbia	nuclear factor of kappa light polypeptide gene enhancer in B-cells inhibitor, alpha	-2.2	n.s.	n.s.
NM_008690	Nfkbie	nuclear factor of kappa light polypeptide gene enhancer in B-cells inhibitor, epsilon	-3.9	-4.5	n.s.
NM_138952	Ripk2	receptor (TNFRSF)-interacting serine-threonine kinase 2	-2.2	-2.1	n.s.
NM_145133	Tifa	TRAF-interacting protein with forkhead-associated domain	-2.1	-1.8	n.s.
NM_009397	Tnfaip3	tumor necrosis factor alpha-induced protein 3	-3.9	-2.5	n.s.
NM_021327	Tnip1	TNFAIP3 interacting protein 1	-3.4	-2.7	n.s.
NM_009421	Traf1	Tnf receptor-associated factor 1	-4.8	-4.9	n.s.

GeneBank ID	Gene symbol	Gene name	Fold-change versus Wt Tnfr1,2-/-	Tnfr1-/-	Tnfr2-/-
		<u>Apoptosis mediators</u>			
NM_007544	Bid	BH3 interacting domain death agonist	-3.1	-2.9	n.s.
NM_007465	Birc2	baculoviral IAP repeat-containing 2	-1.9	n.s.	n.s.
NM_007464	Birc3	baculoviral IAP repeat-containing 3	-2.4	n.s.	n.s.
NM_007609	Casp4	caspase 4, apoptosis-related cysteine peptidase	-3.7	n.s.	n.s.
NM_009808	Casp12	caspase 12	-3.8	-3.2	n.s.
NM_027878	Dram1	DNA-damage regulated autophagy modulator 1	-4.0	-3.0	n.s.
NM_007987	Fas	Fas (TNF receptor superfamily member 6)	-3.3	-3.7	n.s.
NM_010275	Gdnf	glial cell line derived neurotrophic factor	-2.8	-2.2	n.s.
NM_011085	Pik3r1	phosphatidylinositol 3-kinase, regulatory subunit, polypeptide 1 (p85 alpha)	n.s.	2.6	n.s.
NM_009251	Serpina3g	serine (or cysteine) peptidase inhibitor, clade A, member 3G	-9.4	-3.7	n.s.
NM_013671	Sod2	superoxide dismutase 2, mitochondrial	-4.2	-3.4	n.s.
NM_009472	Unc5c	unc-5 homolog C (C. elegans)	-1.9	n.s.	n.s.
NM_001037713	Xaf1	XIAP associated factor 1	-3.0	-2.9	n.s.
		<u>Cell cycle, proliferation and differentiation regulators</u>			
NM_007426	Angpt2	angiopoietin 2	n.s.	2.1	n.s.
NM_007560	Bmpr1b	bone morphogenetic protein receptor, type 1B	-3.4	-2.7	n.s.
NM_010216	Figf	C-fos induced growth factor	5.3	6.4	n.s.
NM_008086	Gas1	growth arrest specific 1	2.8	n.s.	n.s.
NM_010260	Gbp2	guanylate nucleotide binding protein 2	-14.4	-13.5	n.s.
NM_018734	Gbp3	guanylate nucleotide binding protein 3	-20.8	-17.4	n.s.
NM_145741	Gdf10	growth differentiation factor 10	n.s.	4.8	n.s.
NM_008343	Igfbp3	insulin-like growth factor binding protein 3	2.7	n.s.	n.s.
NM_010215	Il4i1	interleukin 4 induced 1	-8.6	-6.8	n.s.
NM_134163	Mbnl3	muscleblind-like 3 (Drosophila)	n.s.	2.1	n.s.
NM_027852	Rarres2	retinoic acid receptor responder (tazarotene induced) 2	-3.6	-3.4	n.s.
NM_009144	Sfrp2	secreted frizzled-related protein 2	-3.0	-2.8	n.s.
NM_011382	Six4	sine oculis-related homeobox 4 homolog (Drosophila)	-1.6	-1.8	n.s.
NM_023814	Tbx18	T-box18	n.s.	-1.8	n.s.

GeneBank ID	Gene symbol	Gene name	Fold-change versus Wt<br>Tnfr1,2-/-	Tnfr1-/-	Tnfr2-/-
		Cytoskeletal proteins and regulators			
NM_009707	Arhgap6	Rho GTPase activating protein 6	n.s.	1.8	n.s.
NM_010662	Krt13	keratin 13	6.5	5.7	n.s.
NM_008471	Krt19	keratin 19	3.4	n.s.	n.s.
NM_172612	Rnd1	Rho family GTPase 1	-3.8	-2.8	n.s.
NM_011521	Sdc4	syndecan 4	n.s.	-2.5	n.s.
NM_011619	Tnnt2	troponin T2, cardiac	-5.0	-5.6	n.s.
NM_017379	Tuba8	tubulin, alpha 8	1.6	n.s.	n.s.
NM_001167860	Wipf3	WAS/WASL interacting protein family, member 3	2.3	n.s.	n.s.
		Proteases			
NM_009615	Adam17	a disintegrin and metallopeptidase domain 17	-1.9	-1.7	n.s.
NM_001003911	Adamts7	a disintegrin-like and metallopeptidase (reprolysin type) with thrombospondin type 1 motif, 7	-6.5	n.s.	n.s.
NM_175314	Adamts9	a disintegrin-like and metallopeptidase (reprolysin type) with thrombospondin type 1 motif, 9	-1.8	-1.9	n.s.
NM_009982	Ctsc	cathepsin C	-5.7	-4.8	n.s.
NM_030711	Erap1	endoplasmic reticulum aminopeptidase 1	-1.8	-2.1	n.s.
NM_172827	Lnpep	leucyl/cystinyl aminopeptidase	1.5	n.s.	n.s.
NM_010809	Mmp3	matrix metallopeptidase 3	-4.8	-3.2	n.s.
NM_013599	Mmp9	matrix metallopeptidase 9	-3.6	-4.0	n.s.
NM_008605	Mmp12	matrix metallopeptidase 12	-2.5	n.s.	n.s.
NM_008607	Mmp13	matrix metallopeptidase 13	-2.4	-2.9	n.s.
NM_011595	Timp3	tissue inhibitor of metalloproteinase 3	1.9	n.s.	n.s.
		Metabolic enzymes			
NM_175236	Adhfe1	alcohol dehydrogenase, iron containing, 1	-5.5	-5.5	n.s.
NM_009667	Ampd3	AMP deaminase 3	-2.0	n.s.	n.s.
NM_007482	Arg1	arginase 1, liver	-2.4	n.s.	n.s.
NM_009705	Arg2	arginase type II	-6.4	-4.5	n.s.
NM_007592	Car8	carbonic anhydrase 8	n.s.	-2.7	n.s.
NM_009890	Ch25h	cholesterol 25-hydroxylase	n.s.	-2.8	n.s.
NM_007825	Cyp7b1	cytochrome P450, family 7, subfamily b, polypeptide 1	-6.4	-10.5	n.s.
NM_025638	Gdpd1	glycerophosphodiester phosphodiesterase domain containing 1	-1.8	n.s.	n.s.

GeneBank ID	Gene symbol	Gene name	Fold-change versus Wt<br>Tnfr1,2-/-	Tnfr1-/-	Tnfr2-/-
NM_010271	Gpd1	glycerol-3-phosphate dehydrogenase 1 (soluble)	1.8	n.s.	n.s.
NM_010358	Gstm1	glutathione S-transferase, mu 1	-2.0	n.s.	n.s.
NM_022415	Ptges	prostaglandin E synthase	-4.2	-4.3	n.s.
NM_011198	Ptgs2	prostaglandin-endoperoxide synthase 2	-3.8	-4.1	n.s.
		Transport proteins and channels			
NM_011076	Abcb1a	ATP-binding cassette, sub-family B (MDR/TAP), member 1A	2.0	2.3	n.s.
NM_011511	Abcc9	ATP-binding cassette, sub-family C (CFTR/MRP), member 9	3.1	5.4	n.s.
NM_011920	Abcg2	ATP-binding cassette, sub-family G (WHITE), member 2	2.5	2.2	n.s.
NM_007752	Cp	ceruloplasmin	-3.8	-3.0	n.s.
NM_028752	Hvcn1	hydrogen voltage-gated channel 1	-2.1	n.s.	n.s.
NM_019697	Kcnd2	potassium voltage-gated channel, Shal-related family, member 2	n.s.	-2.6	n.s.
NM_021342	Kcne4	potassium voltage-gated channel, Isk-related subfamily, gene 4	3.4	3.0	n.s.
NM_008428	Kcnj8	potassium inwardly-rectifying channel, subfamily J, member 8	n.s.	2.8	n.s.
NM_175429	Kctd12b	potassium channel tetramerisation domain containing 12b	3.9	3.0	n.s.
NM_010748	Lyst	lysosomal trafficking regulator	-1.8	n.s.	n.s.
NM_173781	Rab6b	RAB6B, member RAS oncogene family	-3.2	n.s.	-2.6
NM_026405	Rab32	RAB32, member RAS oncogene family	-2.4	-2.5	n.s.
NM_023386	Rtp4	receptor transporter protein 4	-3.5	-3.1	n.s.
NM_172659	Slc2a6	solute carrier family 2 (facilitated glucose transporter), member 6	-27.8	-19.2	n.s.
NM_007514	Slc7a2	solute carrier family 7 (cationic amino acid transporter, y+ system), member 2	-3.5	-3.4	n.s.
NM_011990	Slc7a11	solute carrier family 7 (cationic amino acid transporter, y+ system), member 11	-2.7	-2.7	n.s.
NM_009194	Slc12a2	solute carrier family 12, member 2	2.1	2.8	n.s.
NM_023044	Slc15a3	solute carrier family 15, member 3	-8.8	-4.4	n.s.
NM_001033167	Slc22a23	solute carrier family 22, member 23	n.s.	-2.0	n.s.
NM_026228	Slc39a8	solute carrier family 39 (metal ion transporter), member 8	-3.7	-4.1	n.s.
NM_016917	Slc40a1	solute carrier family 40 (iron-regulated transporter), member 1	n.s.	3.0	n.s.
NM_021398	Slc43a3	solute carrier family 43, member 3	-2.4	n.s.	n.s.
NM_175316	Slco2b1	solute carrier organic anion transporter family, member 2b1	-5.1	-14.3	n.s.

GeneBank ID	Gene symbol	Gene name	Fold-change versus Wt Tnfr1,2-/-	Tnfr1-/-	Tnfr2-/-
NM_028035	Snx10	sorting nexin 10	-2.5	-2.8	n.s.
NM_054098	Steap4	STEAP family member 4	-2.2	-2.0	n.s.
NM_018801	Syt7	synaptotagmin VII	-1.8	-1.8	n.s.
		Membrane proteins			
NM_025779	Ccdc109b	coiled-coil domain containing 109B	n.s.	n.s.	-4.3
		Signaling molecules			
NM_029270	Arhgap24	Rho GTPase activating protein 24	2.8	n.s.	n.s.
NM_009759	Bmx	BMX non-receptor tyrosine kinase	3.6	4.2	n.s.
NM_009895	Cish	cytokine inducible SH2-containing protein	-1.9	n.s.	n.s.
NM_178681	Dgkb	diacylglycerol kinase, beta	2.2	2.5	n.s.
NM_010051	Dkk1	dickkopf homolog 1 (Xenopus laevis)	-1.9	-2.1	n.s.
NM_010276	Gem	GTP binding protein (gene overexpressed in skeletal muscle)	-1.9	n.s.	n.s.
NM_021896	Gucy1a3	guanylate cyclase 1, soluble, alpha 3	2.6	3.0	n.s.
NM_028679	Irak3	interleukin-1 receptor-associated kinase 3	-2.5	n.s.	n.s.
NM_001164268	Kalrn	kalirin, RhoGEF kinase	-2.7	-2.5	n.s.
NM_011943	Map2k6	mitogen-activated protein kinase kinase 6	-6.9	-4.3	n.s.
NM_013875	Pde7b	phosphodiesterase 7B	3.0	n.s.	n.s.
NM_172263	Pde8b	phosphodiesterase 8B	2.1	2.7	n.s.
NM_009658	Plcl1	phospholipase C-like 1	n.s.	2.6	n.s.
NM_008856	Prkch	protein kinase C, eta	n.s.	2.1	n.s.
NM_019688	Rapgef4	Rap guanine nucleotide exchange factor (GEF) 4	2.3	2.2	n.s.
NM_001013386	Rasl10b	RAS-like, family 10, member B	1.5	n.s.	n.s.
NM_009061	Rgs2	regulator of G-protein signaling 2	n.s.	3.7	n.s.
NM_011267	Rgs16	regulator of G-protein signaling 16	-2.6	-2.7	n.s.
NM_019958	Rgs17	regulator of G-protein signaling 17	-8.6	-8.0	n.s.
NM_026446	Rgs19	regulator of G-protein signaling 19	n.s.	-1.7	n.s.
NM_019662	Rrad	Ras-related associated with diabetes	-2.4	n.s.	n.s.
NM_033524	Spred1	sprouty protein with EVH-1 domain 1, related sequence	-1.7	n.s.	n.s.
NM_019636	Tbc1d1	TBC1 domain family, member 1	n.s.	2.3	n.s.
NM_009523	Wnt4	wingless-related MMTV integration site 4	1.9	n.s.	n.s.

GeneBank ID	Gene symbol	Gene name	Fold-change versus Wt Tnfr1,2-/-	Tnfr1-/-	Tnfr2-/-
		Transcription factors and regulators			
NM_010056	Dlx5	distal-less homeobox 5	1.8	2.0	n.s.
NM_010095	Ebf2	early B-cell factor 2	3.5	n.s.	n.s.
NM_013905	Heyl	hairy/enhancer-of-split related with YRPW motif-like	4.7	6.0	n.s.
NM_175606	Hopx	HOP homeobox	5.7	5.9	n.s.
NM_008390	Irf1	interferon regulatory factor 1	-3.4	n.s.	n.s.
NM_010636	Klf12	Kruppel-like factor 12	n.s.	2.9	n.s.
NM_013594	Mbd1	methyl-CpG binding domain protein 1	-8.9	-6.2	n.s.
NM_025282	Mef2c	myocyte enhancer factor 2C	2.2	2.3	n.s.
NM_008687	Nfib	nuclear factor I/B	-2.4	-2.0	n.s.
NM_001083918	OTTMUSG00000010657	predicted gene, OTTMUSG00000010657	-3.2	n.s.	-3.5
NM_001081009	Parp8	poly (ADP-ribose) polymerase family, member 8	-2.2	-2.2	n.s.
NM_030253	Parp9	poly (ADP-ribose) polymerase family, member 9	-2.7	-2.2	n.s.
NM_001039530	Parp14	poly (ADP-ribose) polymerase family, member 14	-3.9	-2.5	n.s.
NM_027285	1700029I01Rik	RIKEN cDNA 1700029I01 gene	-3.4	n.s.	-3.1
NM_020589	Zfp467	zinc finger protein 467	2.2	n.s.	n.s.

n.s.: not significant
