# Supplementary material for: Distinct Contributions of TNF Receptor 1 and 2 to TNF-Induced Glomerular Inflammation in Mice
Source: PLoS One. 2013 Jul 15;8(7):e68167. doi: 10.1371/journal.pone.0068167 (PMC3711912; doi:10.1371/journal.pone.0068167)
Supplement: Table S2 — Differentially expressed genes in TNF-stimulated Tnfr1,2−/− glomeruli compared to wildtype as identified by microarray profiling. (PDF) [file pone.0068167.s003.pdf]

Table S2. Differentially expressed genes in TNF-stimulated *Tnfr1,2*-/- glomeruli compared to wildtype (Wt) as identified by microarray profiling[1].

Affymetrix probe set ID	GeneBank ID	Gene symbol	Gene name	Fold-change versus Wt		
				Tnfr1,2-/-	Tnfr1-/-	Tnfr2-/-
1420380_at	NM_011333	Ccl2	chemokine (C-C motif) ligand 2	-50.0	-42.9	n.s.
1417314_at	NM_008198	Cfb	complement factor B	-35.2	-34.0	n.s.
1427381_at	NM_008392	Irg1	immunoresponsive gene 1	-31.7	-38.1	n.s.
1434015_at	NM_172659	Slc2a6	solute carrier family 2 (facilitated glucose transporter), member 6	-27.8	-19.2	n.s.
1450826_a_at	NM_011315	Saa3	serum amyloid A 3	-25.5	-24.7	n.s.
1449984_at	NM_009140	Cxcl2	chemokine (C-X-C motif) ligand 2	-24.9	-18.0	n.s.
1415989_at 1436003_at 1448162_at 1451314_a_at	NM_011693	Vcam1	vascular cell adhesion molecule 1	-23.8	-22.1	n.s.
1438676_at 1447927_at	NM_194336	Mpa2l	macrophage activation 2 like	-22.8	-10.8	n.s.
1438148_at	NM_203320	Cxcl3	chemokine (C-X-C motif) ligand 3	-22.4	-19.4	n.s.
1418930_at	NM_021274	Cxcl10	chemokine (C-X-C motif) ligand 10	-21.0	-15.0	n.s.
1418392_a_at	NM_018734	Gbp3	guanylate nucleotide binding protein 3	-20.8	-17.4	n.s.
1420591_at	NM_030720	Gpr84	G protein-coupled receptor 84	-16.6	-19.0	n.s.
1424067_at	NM_010493	Icam1	intercellular adhesion molecule 1	-15.4	-9.9	n.s.
1418240_at 1435906_x_at	NM_010260	Gbp2	guanylate nucleotide binding protein 2	-14.4	-13.5	n.s.
1420330_at 1420331_at	NM_019948	Clec4e	C-type lectin domain family 4, member e	-13.2	-10.2	n.s.
1448881_at	NM_017370	Hp	haptoglobin	-13.0	-15.0	n.s.
1421228_at	NM_013654	Ccl7	chemokine (C-C motif) ligand 7	-11.2	-14.7	n.s.
1418126_at	NM_013653	Ccl5	chemokine (C-C motif) ligand 5	-10.6	-5.2	n.s.
1418746_at	NM_019999 NM_001039509	Pnkd	paroxysmal nonkinesiogenic dyskinesia	-10.3	-5.8	n.s.
1424923_at	NM_009251	Serpina3g	serine (or cysteine) peptidase inhibitor, clade A, member 3G	-9.4	-3.7	n.s.
1425253_a_at	NM_013591	Madcam1	mucosal vascular addressin cell adhesion molecule 1	-9.1	-6.1	n.s.
1430837_a_at 1430838_x_at 1453678_at	NM_013594	Mbd1	methyl-CpG binding domain protein 1	-8.9	-6.2	n.s.
1420697_at	NM_023044	Slc15a3	solute carrier family 15, member 3	-8.8	-4.4	n.s.
1419192_at	NM_010215	Il4i1	interleukin 4 induced 1	-8.6	-6.8	n.s.
1450693_at	NM_019958	Rgs17	regulator of G-protein signaling 17	-8.6	-8.0	n.s.
1422962_a_at	NM_010724	Psmb8	proteasome (prosome, macropain) subunit, beta type 8	-8.0	-4.3	n.s.

Affymetrix probe set ID	GeneBank ID	Gene symbol	Gene name	Fold-change versus Wt Tnfr1,2-/-	Tnfr1-/-	Tnfr2-/-
1448632_at	NM_013640	Psmb10	proteasome (prosome, macropain) subunit, beta type 10	-7.9	-5.7	n.s.
1419728_at	NM_009141	Cxcl5	chemokine (C-X-C motif) ligand 5	-7.8	-8.3	n.s.
1426850_a_at	NM_011943	Map2k6	mitogen-activated protein kinase kinase 6	-6.9	-4.3	n.s.
1423091_a_at 1425942_a_at	NM_023122	Gpm6b	glycoprotein m6b	-6.7	-4.5	n.s.
1452339_at	NM_001003911	Adamts7	a disintegrin-like and metallopeptidase (reprolysin type) with thrombospondin type 1 motif, 7	-6.5	n.s.	n.s.
1418847_at	NM_009705	Arg2	arginase type II	-6.4	-4.5	n.s.
1421074_at 1421075_s_at	NM_007825	Cyp7b1	cytochrome P450, family 7, subfamily b, polypeptide 1	-6.4	-10.5	n.s.
1418718_at 1449195_s_at	NM_023158	Cxcl16	chemokine (C-X-C motif) ligand 16	-6.2	-3.6	n.s.
1417292_at	NM_008330	Ifi47	interferon gamma inducible protein 47	-6.1	-3.8	n.s.
1437939_s_at	NM_009982	Ctsc	cathepsin C	-5.7	-4.8	n.s.
1437569_at	NM_023438	Tmem132e	transmembrane protein 132E	-5.6	-4.4	n.s.
1424392_at 1424393_s_at	NM_175236	Adhfe1	alcohol dehydrogenase, iron containing, 1	-5.5	-5.5	n.s.
1450165_at	NM_011408	Slfn2	schlafen 2	-5.5	-3.8	n.s.
1418652_at 1456907_at	NM_008599	Cxcl9	chemokine (C-X-C motif) ligand 9	-5.4	-14.0	n.s.
1417936_at 1448898_at	NM_011338	Ccl9	chemokine (C-C motif) ligand 9	-5.4	-3.9	n.s.
1419212_at	NM_015790	Icosl	icos ligand	-5.4	n.s.	n.s.
1423954_at	NM_009778	C3	complement component 3	-5.2	-4.5	n.s.
1433933_s_at 1454777_at	NM_175316	Slco2b1	solute carrier organic anion transporter family, member 2b1	-5.1	-14.3	n.s.
1451006_at	NM_011723	Xdh	xanthine dehydrogenase	-5.0	n.s.	n.s.
1418726_a_at 1424967_x_at	NM_011619 NM_001130174 NM_001130175 NM_001130176 NM_001130177 NM_001130178 NM_001130179 NM_001130180 NM_001130181	Tnnt2	troponin T2, cardiac	-5.0	-5.6	n.s.
1449498_at 1458297_s_at	NM_010766	Marco	macrophage receptor with collagenous structure	-5.0	-5.6	n.s.
1425294_at	NM_029084	Slamf8	SLAM family member 8	-4.9	n.s.	n.s.
1423602_at	NM_009421	Traf1	Tnf receptor-associated factor 1	-4.8	-4.9	n.s.
1418945_at	NM_010809	Mmp3	matrix metallopeptidase 3	-4.8	-3.2	n.s.
1419658_at	NM_010398	H2-T23	histocompatibility 2, T region locus 23	-4.6	-3.7	n.s.
1435512_at 1436099_at	NM_177716	AI836003	expressed sequence AI836003	-4.5	-2.8	n.s.

Affymetrix probe set ID	GeneBank ID	Gene symbol	Gene name	Fold-change versus Wt Tnfr1,2-/-	Tnfr1-/-	Tnfr2-/-
1418099_at	NM_011610	Tnfrsf1b	tumor necrosis factor receptor superfamily, member 1b	-4.4	n.s.	-4.9
1417813_at	NM_019777	Ikbke	inhibitor of kappaB kinase epsilon	-4.2	-3.1	n.s.
1417193_at 1417194_at 1448610_a_at	NM_013671	Sod2	superoxide dismutase 2, mitochondrial	-4.2	-3.4	n.s.
1425681_a_at	NM_023043	Prnd	prion protein dublet	-4.2	-5.1	n.s.
1439747_at 1449449_at 1449450_at	NM_022415	Ptges	prostaglandin E synthase	-4.2	-4.3	n.s.
1436778_at 1436779_at	NM_007807	Cybb	cytochrome b-245, beta polypeptide	-4.2	-3.5	n.s.
1419627_s_at 1425951_a_at	NM_020001	Clec4n	C-type lectin domain family 4, member n	-4.1	-2.7	n.s.
1416625_at	NM_009776	Serping1	serine (or cysteine) peptidase inhibitor, clade G, member 1	-4.1	-3.4	n.s.
1424524_at	NM_027878	Dram1	DNA-damage regulated autophagy modulator 1	-4.0	-3.0	n.s.
1427313_at	NM_008967	Ptgir	prostaglandin I receptor (IP)	-4.0	-3.1	n.s.
1451564_at	NM_001039530	Parp14	poly (ADP-ribose) polymerase family, member 14	-3.9	-2.5	n.s.
1431843_a_at 1458299_s_at	NM_008690	Nfkbie	nuclear factor of kappa light polypeptide gene enhancer in B-cells inhibitor, epsilon	-3.9	-4.5	n.s.
1450696_at	NM_013585	Psmb9	proteasome (prosome, macropain) subunit, beta type 9 (large multifunctional peptidase 2)	-3.9	n.s.	n.s.
1433699_at	NM_009397	Tnfaip3	tumor necrosis factor alpha-induced protein 3	-3.9	-2.5	n.s.
1417495_x_at 1417496_at 1417497_at 1448734_at	NM_007752	Cp	ceruloplasmin	-3.8	-3.0	n.s.
1417263_at	NM_011198	Ptgs2	prostaglandin-endoperoxide synthase 2	-3.8	-4.1	n.s.
1419132_at	NM_011905	Tlr2	toll-like receptor 2	-3.8	-3.6	n.s.
1421408_at	NM_030691	Igsf6	immunoglobulin superfamily, member 6	-3.8	-3.5	n.s.
1455197_at	NM_172612	Rnd1	Rho family GTPase 1	-3.8	-2.8	n.s.
1418981_at	NM_009808	Casp12	caspase 12	-3.8	-3.2	n.s.
1416832_at	NM_026228	Slc39a8	solute carrier family 39 (metal ion transporter), member 8	-3.7	-4.1	n.s.
1424254_at	NM_026820 NM_001112715	Ifitm1	interferon induced transmembrane protein 1	-3.7	-5.3	n.s.
1453196_a_at	NM_011854	Oasl2	2'-5' oligoadenylate synthetase-like 2	-3.7	n.s.	n.s.
1449591_at	NM_007609	Casp4	caspase 4, apoptosis-related cysteine peptidase	-3.7	n.s.	n.s.

Affymetrix probe set ID	GeneBank ID	Gene symbol	Gene name	Fold-change versus Wt Tnfr1,2-/-	Tnfr1-/-	Tnfr2-/-
1419209_at<br>1441855_x_at<br>1457644_s_at	NM_008176	Cxcl1	chemokine (C-X-C motif) ligand 1	-3.7	n.s.	n.s.
1448136_at	NM_015744	Enpp2	ectonucleotide pyrophosphatase/ phosphodiesterase 2	-3.7	n.s.	n.s.
1428538_s_at<br>1437902_s_at	NM_027852	Rarres2	retinoic acid receptor responder (tazarotene induced) 2	-3.6	-3.4	n.s.
1434372_at	NM_001177351	AW112010	expressed sequence AW112010	-3.6	-3.5	n.s.
1416298_at	NM_013599	Mmp9	matrix metallopeptidase 9	-3.6	-4.0	n.s.
1418580_at	NM_023386	Rtp4	receptor transporter protein 4	-3.5	-3.1	n.s.
1422648_at<br>1426008_a_at<br>1436555_at	NM_007514<br>NM_001044740	Slc7a2	solute carrier family 7 (cationic amino acid transporter, y+ system), member 2	-3.5	-3.4	n.s.
1437312_at	NM_007560	Bmpr1b	bone morphogenetic protein receptor, type 1B	-3.4	-2.7	n.s.
1427689_a_at	NM_021327	Tnip1	TNFAIP3 interacting protein 1	-3.4	-2.7	n.s.
1448436_a_at	NM_008390	Irf1	interferon regulatory factor 1	-3.4	n.s.	n.s.
1422953_at	NM_008039	Fpr2	formyl peptide receptor 2	-3.4	-3.9	n.s.
1436574_at	NM_027285	1700029I01Rik	RIKEN cDNA 1700029I01 gene	-3.4	n.s.	-3.1
1419691_at	NM_009921	Camp	cathelicidin antimicrobial peptide	-3.3	-3.2	n.s.
1460251_at	NM_007987	Fas	Fas (TNF receptor superfamily member 6)	-3.3	-3.7	n.s.
1421207_at	NM_008501<br>NM_001039537	Lif	leukemia inhibitory factor	-3.2	n.s.	n.s.
1434510_at	NM_011864	Papss2	3'-phosphoadenosine 5'-phosphosulfate synthase 2	-3.2	-3.4	n.s.
1424784_at	NM_001083918	Gm13139	predicted gene 13139	-3.2	n.s.	-3.5
1451798_at	NM_031167	Il1rn	interleukin 1 receptor antagonist	-3.2	-3.0	n.s.
1419529_at	NM_031252	Il23a	interleukin 23, alpha subunit p19	-3.2	-3.5	n.s.
1434914_at<br>1437107_at<br>1460617_s_at	NM_173781	Rab6b	RAB6B, member RAS oncogene family	-3.2	n.s.	-2.6
1451544_at	NM_145391	Tapbpl	TAP binding protein-like	-3.2	-2.5	n.s.
1420804_s_at	NM_010819	Clec4d	C-type lectin domain family 4, member d	-3.2	n.s.	n.s.
1460220_a_at	NM_007778	Csf1	colony stimulating factor 1 (macrophage)	-3.2	n.s.	n.s.
1429543_at<br>1453188_at	AK018095<br>AK078961	6230424C14Rik	RIKEN cDNA 6230424C14 gene	-3.1	n.s.	n.s.
1416051_at<br>1457664_x_at	NM_013484	C2	complement component 2 (within H-2S)	-3.1	-2.9	n.s.
1417045_at	NM_007544	Bid	BH3 interacting domain death agonist	-3.1	-2.9	n.s.
1426528_at<br>1435349_at	NM_010939	Nrp2	neuropilin 2	-3.1	-2.4	n.s.
1427691_a_at	NM_010509	Ifnar2	interferon (alpha and beta) receptor 2	-3.1	n.s.	n.s.
1443698_at	NM_001037713	Xaf1	XIAP associated factor 1	-3.0	-2.9	n.s.

Affymetrix probe set ID	GeneBank ID	Gene symbol	Gene name	Fold-change versus Wt		
				Tnfr1,2-/-	Tnfr1-/-	Tnfr2-/-
1438498_at	NM_001029929	Zmynd15	zinc finger, MYND-type containing 15	-3.0	n.s.	n.s.
1448201_at	NM_009144	Sfrp2	secreted frizzled-related protein 2	-3.0	-2.8	n.s.
1421812_at	NM_009318<br>NM_001025313	Tapbp	TAP binding protein	-2.9	-2.4	n.s.
1419202_at	NM_009977	Cst7	cystatin F (leukocystatin)	-2.9	-2.9	n.s.
1416295_a_at<br>1416296_at	NM_013563	Il2rg	interleukin 2 receptor, gamma chain	-2.9	-2.0	n.s.
1427736_a_at	NM_017466	Ccrl2	chemokine (C-C motif) receptor-like 2	-2.9	n.s.	n.s.
1426784_at	NM_172570	Trim47	tripartite motif-containing 47	-2.9	n.s.	n.s.
1420499_at<br>1429692_s_at	NM_008102	Gch1	GTP cyclohydrolase 1	-2.9	-2.8	n.s.
1421366_at	NM_021364<br>NM_001038604	Clec5a	C-type lectin domain family 5, member a	-2.8	-2.7	n.s.
1419080_at	NM_010275	Gdnf	glial cell line derived neurotrophic factor	-2.8	-2.2	n.s.
1435596_at<br>1440454_at	NM_175437	Pion	pigeon homolog (Drosophila)	-2.8	-3.3	n.s.
1416897_at	NM_030253	Parp9	poly (ADP-ribose) polymerase family, member 9	-2.7	-2.2	n.s.
1420413_at	NM_011990	Slc7a11	solute carrier family 7 (cationic amino acid transporter, y+ system), member 11	-2.7	-2.7	n.s.
1417189_at	NM_011190<br>NM_001029855	Psme2	proteasome (prosome, macropain) 28 subunit, beta	-2.7	-2.5	n.s.
1436066_at<br>1457690_at	NM_177357<br>NM_001164268	Kalrn	kalirin, RhoGEF kinase	-2.7	-2.5	n.s.
1417688_at<br>1443827_x_at	NM_030565	Fam20c	family with sequence similarity 20, member C	-2.7	-2.7	n.s.
1450517_at<br>1460309_at	NM_009317	Tal2	T-cell acute lymphocytic leukemia 2	-2.6	n.s.	n.s.
1426037_a_at	NM_011267	Rgs16	regulator of G-protein signaling 16	-2.6	-2.7	n.s.
1428767_at	NM_026960	Gsdmd	gasdermin D	-2.6	-2.3	n.s.
1417470_at	NM_030255	Apobec3	apolipoprotein B editing complex 3	-2.6	n.s.	n.s.
1431055_a_at	NM_028035	Snx10	sorting nexin 10	-2.5	-2.8	n.s.
1426971_at	NM_023738	Ube1l	ubiquitin-activating enzyme E1-like	-2.5	n.s.	n.s.
1448550_at	NM_008489	Lbp	lipopolysaccharide binding protein	-2.5	-2.5	n.s.
1426223_at	NM_028341	Ttc39c	tetratricopeptide repeat domain 39C	-2.5	-2.4	n.s.
1419534_at	NM_138648	Olr1	oxidized low density lipoprotein (lectin-like) receptor 1	-2.5	-2.6	n.s.
1449153_at	NM_008605	Mmp12	matrix metallopeptidase 12	-2.5	n.s.	n.s.
1435040_at	NM_028679	Irak3	interleukin-1 receptor-associated kinase 3	-2.5	n.s.	n.s.
1460407_at	NM_019866	Spib	Spi-B transcription factor (Spi-1/PU.1 related)	-2.5	n.s.	n.s.
1422788_at	NM_021398	Slc43a3	solute carrier family 43, member 3	-2.4	n.s.	n.s.

Affymetrix probe set ID	GeneBank ID	Gene symbol	Gene name	Fold-change versus Wt Tnfr1,2-/-	Tnfr1-/-	Tnfr2-/-
1435208_at	NM_001013371	Dtx3l	deltex 3-like (Drosophila)	-2.4	-2.5	n.s.
1448964_at	NM_009789	S100g	S100 calcium binding protein G	-2.4	n.s.	n.s.
1419549_at	NM_007482	Arg1	arginase 1, liver	-2.4	n.s.	n.s.
1421392_a_at	NM_007464	Birc3	baculoviral IAP repeat-containing 3	-2.4	n.s.	n.s.
1417291_at	NM_011609	Tnfrsf1a	tumor necrosis factor receptor superfamily, member 1a	-2.4	n.s.	n.s.
1424354_at	NM_197986	Tmem140	transmembrane protein 140	-2.4	n.s.	n.s.
1427680_a_at 1454834_at	NM_008687	Nfib	nuclear factor I/B	-2.4	-2.0	n.s.
1453181_x_at	NM_011636	Plscr1	phospholipid scramblase 1	-2.4	-2.1	n.s.
1417822_at	NM_033075	D17H6S56 E-5	DNA segment, Chr 17, human D6S56E 5	-2.4	-2.5	n.s.
1416527_at	NM_026405	Rab32	RAB32, member RAS oncogene family	-2.4	-2.5	n.s.
1417256_at	NM_008607	Mmp13	matrix metallopeptidase 13	-2.4	-2.9	n.s.
1422562_at	NM_019662	Rrad	Ras-related associated with diabetes	-2.4	n.s.	n.s.
1432032_a_at	NM_009711	Artn	artemin	-2.4	n.s.	n.s.
1455251_at	NM_001033228	Itga1	integrin alpha 1	-2.3	-2.4	n.s.
1448775_at	NM_008328 NM_001045481	Ifi203	interferon activated gene 203	-2.3	-2.7	n.s.
1417172_at	NM_019949	Ube2l6	ubiquitin-conjugating enzyme E2L 6	-2.3	-2.3	n.s.
1423754_at	NM_025378	Ifitm3	interferon induced transmembrane protein 3	-2.3	n.s.	n.s.
1452050_at	NM_177343	Camk1d	calcium/calmodulin-dependent protein kinase ID	-2.3	n.s.	n.s.
1420088_at 1438157_s_at 1448306_at 1449731_s_at	NM_010907	Nfkbia	nuclear factor of kappa light polypeptide gene enhancer in B-cells inhibitor, alpha	-2.2	n.s.	n.s.
1460197_a_at	NM_054098	Steap4	STEAP family member 4	-2.2	-2.0	n.s.
1449305_at	NM_007972	F10	coagulation factor X	-2.2	n.s.	n.s.
1425669_at 1429284_at	NM_178061	Mobkl2b	MOB1, Mps one binder kinase activator-like 2B (yeast)	-2.2	-2.2	n.s.
1423878_at	NM_027863	Gypc	glycophorin C	-2.2	n.s.	n.s.
1421236_at 1450173_at	NM_138952	Ripk2	receptor (TNFRSF)-interacting serine-threonine kinase 2	-2.2	-2.1	n.s.
1433812_at	NM_001163170	Lix1l	Lix1-like	-2.2	n.s.	n.s.
1450808_at	NM_013521	Fpr1	formyl peptide receptor 1	-2.2	n.s.	n.s.
1448377_at	NM_011414	Slpi	secretory leukocyte peptidase inhibitor	-2.2	n.s.	n.s.
1419468_at	NM_025809	Clec14a	C-type lectin domain family 14, member a	-2.2	n.s.	n.s.
1451474_a_at	NM_001081009	Parp8	poly (ADP-ribose) polymerase family, member 8	-2.2	-2.2	n.s.
1419714_at	NM_021893	Cd274	CD274 antigen	-2.1	n.s.	n.s.

Affymetrix probe set ID	GeneBank ID	Gene symbol	Gene name	Fold-change versus Wt Tnfr1,2-/-	Tnfr1-/-	Tnfr2-/-
1436562_at 1456890_at	NM_172689	Ddx58	DEAD (Asp-Glu-Ala-Asp) box polypeptide 58	-2.1	n.s.	n.s.
1417056_at	NM_011189	Psme1	proteasome (prosome, macropain) 28 subunit, alpha	-2.1	n.s.	n.s.
1450534_x_at	NM_019909	H2-K1	histocompatibility 2, K1, K region	-2.1	n.s.	n.s.
1435488_at	NM_026788	Mthfd2l	methylenetetrahydrofolate dehydrogenase 2-like	-2.1	-2.0	n.s.
1450424_a_at	NM_010531	Il18bp	interleukin 18 binding protein	-2.1	n.s.	n.s.
1426501_a_at	NM_145133	Tifa	TRAF-interacting protein with forkhead-associated domain	-2.1	-1.8	n.s.
1424032_at	NM_028752 NM_001042489	Hvcn1	hydrogen voltage-gated channel 1	-2.1	n.s.	n.s.
1418163_at	NM_021297	Tlr4	toll-like receptor 4	-2.0	n.s.	n.s.
1448330_at	NM_010358	Gstm1	glutathione S-transferase, mu 1	-2.0	n.s.	n.s.
1422573_at	NM_009667	Ampd3	AMP deaminase 3	-2.0	n.s.	n.s.
1436545_at 1455711_at	NM_172442	Dtx4	deltex 4 homolog (Drosophila)	-2.0	-1.8	n.s.
1417928_at	NM_019417	Pdlim4	PDZ and LIM domain 4	-1.9	n.s.	n.s.
1417066_at	NM_023341	Cabc1	chaperone, ABC1 activity of bc1 complex like (S. pombe)	-1.9	n.s.	n.s.
1426063_a_at	NM_010276	Gem	GTP binding protein (gene overexpressed in skeletal muscle)	-1.9	n.s.	n.s.
1421858_at	NM_009615	Adam17	a disintegrin and metallopeptidase domain 17	-1.9	-1.7	n.s.
1422924_at	NM_009404	Tnfsf9	tumor necrosis factor (ligand) superfamily, member 9	-1.9	n.s.	n.s.
1425869_a_at	NM_011183	Psen2	presenilin 2	-1.9	n.s.	n.s.
1427127_x_at 1452318_a_at	NM_010478 NM_010479	Hspa1b	heat shock protein 1B	-1.9	n.s.	n.s.
1419592_at	NM_009472	Unc5c	unc-5 homolog C (C. elegans)	-1.9	n.s.	n.s.
1420360_at 1458232_at	NM_010051	Dkk1	dickkopf homolog 1 (Xenopus laevis)	-1.9	-2.1	n.s.
1423909_at 1441811_x_at	NM_025326	Tmem176a	transmembrane protein 176A	-1.9	n.s.	n.s.
1418854_at	NM_007465	Birc2	baculoviral IAP repeat-containing 2	-1.9	n.s.	n.s.
1441096_at	NM_177709	Tusc5	tumor suppressor candidate 5	-1.9	n.s.	n.s.
1436739_at	NM_177322	Agtr1a	angiotensin II receptor, type 1a	-1.9	n.s.	n.s.
1448724_at	NM_009895	Cish	cytokine inducible SH2-containing protein	-1.9	n.s.	n.s.
1437785_at	NM_175314	Adamts9	a disintegrin-like and metallopeptidase (reprolysin type) with thrombospondin type 1 motif, 9	-1.8	-1.9	n.s.
1424076_at	NM_025638	Gdpd1	glycerophosphodiester phosphodiesterase domain containing 1	-1.8	n.s.	n.s.
1455660_at	NM_007780	Csf2rb	colony stimulating factor 2 receptor, beta, low-affinity (granulocyte-macrophage)	-1.8	n.s.	n.s.

Affymetrix probe set ID	GeneBank ID	Gene symbol	Gene name	Fold-change versus Wt Tnfr1,2-/-	Tnfr1-/-	Tnfr2-/-
1416942_at	NM_030711	Erap1	endoplasmic reticulum aminopeptidase 1	-1.8	-2.1	n.s.
1435431_at	NM_001101430	Psmg4	proteasome (prosome, macropain) assembly chaperone 4	-1.8	n.s.	n.s.
1417009_at	NM_023143	C1r	complement component 1, r subcomponent	-1.8	n.s.	n.s.
1434674_at	NM_010748	Lyst	lysosomal trafficking regulator	-1.8	n.s.	n.s.
1460014_at	NM_172623<br>NM_001033922	Treml4	triggering receptor expressed on myeloid cells-like 4	-1.8	-1.6	n.s.
1460081_at	NM_018801<br>NM_173067<br>NM_173068	Syt7	synaptotagmin VII	-1.8	-1.8	n.s.
1416998_at	NM_021511	Rrs1	RRS1 ribosome biogenesis regulator homolog (S. cerevisiae)	-1.7	-1.8	n.s.
1433935_at	AK049415	AU020206	expressed sequence AU020206	-1.7	n.s.	n.s.
1442608_at	NM_001033534	Layn	layilin	-1.7	n.s.	n.s.
1460116_s_at	NM_033524	Spred1	sprouty protein with EVH-1 domain 1, related sequence	-1.7	n.s.	n.s.
1428468_at	NM_001081343	3110043O21Rik	RIKEN cDNA 3110043O21 gene	-1.7	n.s.	n.s.
1449360_at	NM_007781	Csf2rb2	colony stimulating factor 2 receptor, beta 2, low-affinity (granulocyte-macrophage)	-1.7	n.s.	n.s.
1424711_at	NM_001033759<br>NM_031997	Tmem2	transmembrane protein 2	-1.7	n.s.	n.s.
1449399_a_at	NM_008361	Il1b	interleukin 1 beta	-1.7	-2.3	n.s.
1455358_at	NM_021477<br>NM_183188	A2bp1	Ataxin 2 binding protein 1	-1.7	-2.0	n.s.
1421358_at	NM_013819	H2-M3	histocompatibility 2, M region locus 3	-1.7	n.s.	n.s.
1425767_a_at	NM_011382	Six4	sine oculis-related homeobox 4 homolog (Drosophila)	-1.6	-1.8	n.s.
1446761_at	C77659	D8Ertd56e	DNA segment, Chr 8, ERATO Doi 56, expressed	-1.6	n.s.	n.s.
1438664_at	NM_011158	Prkar2b	protein kinase, cAMP dependent regulatory, type II beta	-1.6	n.s.	n.s.
1448940_at	NM_009277	Trim21	tripartite motif-containing 21	-1.6	n.s.	n.s.
1428250_at	NM_029771	Gper	G protein-coupled estrogen receptor 1	-1.6	n.s.	n.s.
1437929_at	NM_172826	Dact2	dapper homolog 2, antagonist of beta-catenin (xenopus)	-1.6	n.s.	n.s.
1456377_x_at	NM_172397	Limd2	LIM domain containing 2	-1.5	n.s.	n.s.
1422603_at	NM_201239<br>NM_021472	Rnase4	ribonuclease, RNase A family 4	-1.5	n.s.	n.s.
1439155_at	NM_010792	Mettl1	methyltransferase-like 1	-1.5	n.s.	n.s.
1436173_at	NM_015802	Dlc1	deleted in liver cancer 1	-1.5	n.s.	n.s.
1457728_at	NM_001166213	Fam129c	family with sequence similarity 129, member C	-1.5	n.s.	n.s.
1439326_at	NM_172827	Lnpep	leucyl/cystinyl aminopeptidase	1.5	n.s.	n.s.

Affymetrix probe set ID	GeneBank ID	Gene symbol	Gene name	Fold-change versus Wt Tnfr1,2-/-	Tnfr1-/-	Tnfr2-/-
1433566_at	NM_001013386	Rasl10b	RAS-like, family 10, member B	1.5	n.s.	n.s.
1446771_at	NM_017379	Tuba8	tubulin, alpha 8	1.6	n.s.	n.s.
1460011_at	NM_175475	Cyp26b1	cytochrome P450, family 26, subfamily b, polypeptide 1	1.6	n.s.	n.s.
1437025_at	NM_007642	CD28	CD28 antigen	1.8	n.s.	n.s.
1439396_x_at	NM_010271	Gpd1	glycerol-3-phosphate dehydrogenase 1 (soluble)	1.8	n.s.	n.s.
1449863_a_at	NM_010056<br>NM_198854	Dlx5	distal-less homeobox 5	1.8	2.0	n.s.
1417168_a_at	NM_016808<br>NM_198091<br>NM_198092	Usp2	ubiquitin specific peptidase 2	1.8	n.s.	n.s.
1452411_at	NM_172528	Lrrc1	leucine rich repeat containing 1	1.8	n.s.	n.s.
1434954_at	NM_019579	Mpp5	membrane protein, palmitoylated 5 (MAGUK p55 subfamily member 5)	1.9	n.s.	n.s.
1438404_at	NM_080563	Rnf144a	ring finger protein 144A	1.9	n.s.	n.s.
1454966_at	NM_001001309	Itga8	integrin alpha 8	1.9	2.7	n.s.
1450782_at	NM_009523	Wnt4	wingless-related MMTV integration site 4	1.9	n.s.	n.s.
1449335_at	NM_011595	Timp3	tissue inhibitor of metalloproteinase 3	1.9	n.s.	n.s.
1420979_at	NM_011035	Pak1	p21 (CDKN1A)-activated kinase 1	2.0	n.s.	n.s.
1447886_at	AK002860	0610040B 09Rik	RIKEN cDNA 0610040B09 gene	2.0	n.s.	n.s.
1437605_at	NM_130456	Nphs2	nephrosis 2 homolog, podocin (human)	2.0	n.s.	n.s.
1419758_at	NM_011076	Abcb1a	ATP-binding cassette, sub-family B (MDR/TAP), member 1A	2.0	2.3	n.s.
1418643_at	NM_025359	Tspan13	tetraspanin 13	2.0	n.s.	n.s.
1418182_at	NM_008975	Ptp4a3	protein tyrosine phosphatase 4a3	2.0	n.s.	n.s.
1417622_at	NM_009194	Slc12a2	solute carrier family 12, member 2	2.1	2.8	n.s.
1442051_at	NM_019469<br>NM_178216	Hist2h3c1	histone cluster 2, H3c1	2.1	n.s.	n.s.
1435741_at<br>1437989_at	NM_172263	Pde8b	phosphodiesterase 8B	2.1	2.7	n.s.
1429144_at	NM_028802<br>NM_001042671	Prei4	preimplantation protein 4	2.1	n.s.	n.s.
1441727_s_at	NM_020589	Zfp467	zinc finger protein 467	2.2	n.s.	n.s.
1424852_at	NM_025282	Mef2c	myocyte enhancer factor 2C	2.2	2.3	n.s.
1437347_at	NM_007904	Ednrb	endothelin receptor type B	2.2	n.s.	n.s.
1455361_at	NM_178681	Dgkb	diacylglycerol kinase, beta	2.2	2.5	n.s.
1456391_at	NM_001134741	Tdrd5	tudor domain containing 5	2.2	n.s.	n.s.
1433959_at	NM_177086	Zmat4	zinc finger, matrin type 4	2.3	n.s.	n.s.
1438325_at	NM_007963	Evi1	ecotropic viral integration site 1	2.3	2.4	n.s.

Affymetrix probe set ID	GeneBank ID	Gene symbol	Gene name	Fold-change versus Wt		
				Tnfr1,2-/-	Tnfr1-/-	Tnfr2-/-
1425518_at	NM_019688	Rapgef4	Rap guanine nucleotide exchange factor (GEF) 4	2.3	2.2	n.s.
1455301_at	NM_001167860 NM_001167861	Wipf3	WAS/WASL interacting protein family, member 3	2.3	n.s.	n.s.
1422869_at	NM_008587	Mertk	c-mer proto-oncogene tyrosine kinase	2.4	n.s.	n.s.
1416129_at	NM_133753	Errfi1	ERBB receptor feedback inhibitor 1	2.4	2.3	n.s.
1417439_at	NM_054042	Cd248	CD248 antigen, endosialin	2.5	n.s.	n.s.
1441228_at	NM_001109914	Apold1	apolipoprotein L domain containing 1	2.5	n.s.	n.s.
1422906_at	NM_011920	Abcg2	ATP-binding cassette, sub-family G (WHITE), member 2	2.5	2.2	n.s.
1420534_at 1434141_at	NM_021896	Gucy1a3	guanylate cyclase 1, soluble, alpha 3	2.6	3.0	n.s.
1416892_s_at 1448509_at	NM_025626	Fam107b	family with sequence similarity 107, member B	2.7	3.2	n.s.
1455050_at	NM_178791	Vstm4	V-set and transmembrane domain containing 4	2.7	2.6	n.s.
1423062_at	NM_008343	Igfbp3	insulin-like growth factor binding protein 3	2.7	n.s.	n.s.
1416164_at	NM_011812	Fbln5	fibulin 5	2.7	2.9	n.s.
1424842_a_at	NM_029270 NM_146161	Arhgap24	Rho GTPase activating protein 24	2.8	n.s.	n.s.
1416855_at	NM_008086	Gas1	growth arrest specific 1	2.8	n.s.	n.s.
1435894_at	AK047704	C030014L02	hypothetical protein C030014L02	2.8	n.s.	n.s.
1435321_at	NM_001001980	Limch1	LIM and calponin homology domains 1	2.9	3.1	n.s.
1420838_at 1435196_at	NM_008745 NM_001025074	Ntrk2	neurotrophic tyrosine kinase, receptor, type 2	2.9	4.0	n.s.
1435184_at	NM_008728 NM_001039181	Npr3	natriuretic peptide receptor 3	2.9	2.7	n.s.
1445539_at	NM_013875	Pde7b	phosphodiesterase 7B	3.0	n.s.	n.s.
1420408_a_at 1435751_at 1435752_s_at	NM_011511 NM_021041 NM_021042 NM_001044720	Abcc9	ATP-binding cassette, sub-family C (CFTR/MRP), member 9	3.1	5.4	n.s.
1425180_at 1425181_at	NM_144906	Sgip1	SH3-domain GRB2-like (endophilin) interacting protein 1	3.2	n.s.	n.s.
1422567_at 1454942_at	NM_022018	Niban	niban protein	3.3	2.8	n.s.
1418156_at	NM_021342	Kcne4	potassium voltage-gated channel, Isk-related subfamily, gene 4	3.4	3.0	n.s.
1417156_at	NM_008471	Krt19	keratin 19	3.4	n.s.	n.s.
1418494_at	NM_010095	Ebf2	early B-cell factor 2	3.5	n.s.	n.s.
1426622_a_at	NM_027455	Qpct	glutaminyl-peptide cyclotransferase (glutaminyl cyclase)	3.5	3.1	n.s.
1433825_at 1455917_at	NM_008746 NM_182809	Ntrk3	neurotrophic tyrosine kinase, receptor, type 3	3.5	n.s.	n.s.
1422084_at	NM_009759	Bmx	BMX non-receptor tyrosine kinase	3.6	4.2	n.s.

Affymetrix probe set ID	GeneBank ID	Gene symbol	Gene name	Fold-change versus Wt Tnfr1,2-/-	Tnfr1-/-	Tnfr2-/-
1440355_at	NM_175429	Kctd12b	potassium channel tetramerisation domain containing 12b	3.9	3.0	n.s.
1451478_at	NM_001039554<br>NM_020009	Angptl7	angiopoietin-like 7	4.0	n.s.	n.s.
1429579_at	NM_001033322	Gucy1a2	guanylate cyclase 1, soluble, alpha 2	4.0	4.1	n.s.
1425846_a_at	NM_021371	Caln1	calneuron 1	4.1	4.5	n.s.
1419302_at	NM_013905	Heyl	hairy/enhancer-of-split related with YRPW motif-like	4.7	6.0	n.s.
1419405_at	NM_026523	Nmb	neuromedin B	5.1	5.0	n.s.
1438953_at<br>1438954_x_at<br>1449528_at	NM_010216	Figf	C-fos induced growth factor	5.3	6.4	n.s.
1428662_a_at<br>1451776_s_at	NM_175606	Hopx	HOP homeobox	5.7	5.9	n.s.
1433691_at	NM_016854	Ppp1r3c	protein phosphatase 1, regulatory (inhibitor) subunit 3C	6.0	5.3	n.s.
1422454_at	NM_010662	Krt13	keratin 13	6.5	5.7	n.s.
1436268_at	NM_001013741	Ddn	dendrin	6.9	n.s.	n.s.
1428664_at	NM_011702	Vip	vasoactive intestinal polypeptide	11.2	9.5	n.s.

[1]Genes are listed according to fold-change of *Tnfr1,2-/-* versus wildtype (Wt) glomeruli. For each gene all identifying probe sets are listed. Multiple GeneBank IDs are given for probe sets detecting multiple transcripts of one gene.
n.s.: not significant.
