# Supplementary material for: Distinct Contributions of TNF Receptor 1 and 2 to TNF-Induced Glomerular Inflammation in Mice
Source: PLoS One. 2013 Jul 15;8(7):e68167. doi: 10.1371/journal.pone.0068167 (PMC3711912; doi:10.1371/journal.pone.0068167)
Supplement: Table S3 — Differentially expressed genes in TNF-stimulated Tnfr1−/− glomeruli compared to wildtype as identified by microarray profiling. (PDF) [file pone.0068167.s004.pdf]

Table S3. Differentially expressed genes in TNF-stimulated *Tnfr1*-/- glomeruli compared to wildtype (Wt) as identified by microarray profiling[1].

Affymetrix probe set ID	GeneBank ID	Gene symbol	Gene name	Fold-change versus Wt		
				Tnfr1-/-	Tnfr2-/-	Tnfr1,2-/-
1420380_at	NM_011333	Ccl2	chemokine (C-C motif) ligand 2	-42.9	n.s.	-50.0
1427381_at	NM_008392	Irg1	immunoresponsive gene 1	-38.1	n.s.	-31.7
1417314_at	NM_008198	Cfb	complement factor B	-34.0	n.s.	-35.2
1450826_a_at	NM_011315	Saa3	serum amyloid A 3	-24.7	n.s.	-25.5
1415989_at 1436003_at 1448162_at 1451314_a_at	NM_011693	Vcam1	vascular cell adhesion molecule 1	-22.1	n.s.	-23.8
1438148_at	NM_203320	Cxcl3	chemokine (C-X-C motif) ligand 3	-19.4	n.s.	-22.4
1434015_at	NM_172659	Slc2a6	solute carrier family 2 (facilitated glucose transporter), member 6	-19.2	n.s.	-27.8
1420591_at	NM_030720	Gpr84	G protein-coupled receptor 84	-19.0	n.s.	-16.6
1449984_at	NM_009140	Cxcl2	chemokine (C-X-C motif) ligand 2	-18.0	n.s.	-24.9
1418392_a_at	NM_018734	Gbp3	guanylate nucleotide binding protein 3	-17.4	n.s.	-20.8
1418930_at	NM_021274	Cxcl10	chemokine (C-X-C motif) ligand 10	-15.0	n.s.	-21.0
1448881_at	NM_017370	Hp	haptoglobin	-15.0	n.s.	-13.0
1421228_at	NM_013654	Ccl7	chemokine (C-C motif) ligand 7	-14.7	n.s.	-11.2
1433933_s_at	NM_175316	Slco2b1	solute carrier organic anion transporter family, member 2b1	-14.3	n.s.	-5.1
1418652_at	NM_008599	Cxcl9	chemokine (C-X-C motif) ligand 9	-14.0	n.s.	-5.4
1418240_at 1435906_x_at	NM_010260	Gbp2	guanylate nucleotide binding protein 2	-13.5	n.s.	-14.4
1438676_at 1447927_at	NM_194336	Mpa2l	macrophage activation 2 like	-10.8	n.s.	-22.8
1421074_at 1421075_s_at	NM_007825	Cyp7b1	cytochrome P450, family 7, subfamily b, polypeptide 1	-10.5	n.s.	-6.4
1420330_at 1420331_at	NM_019948	Clec4e	C-type lectin domain family 4, member e	-10.2	n.s.	-13.2
1424067_at	NM_010493	Icam1	intercellular adhesion molecule 1	-9.9	n.s.	-15.4
1419728_at	NM_009141	Cxcl5	chemokine (C-X-C motif) ligand 5	-8.3	n.s.	-7.8
1450693_at	NM_019958	Rgs17	regulator of G-protein signaling 17	-8.0	n.s.	-8.6
1419192_at	NM_010215	Il4i1	interleukin 4 induced 1	-6.8	n.s.	-8.6
1430837_a_at 1430838_x_at 1453678_at	NM_013594	Mbd1	methyl-CpG binding domain protein 1	-6.2	n.s.	-8.9
1425253_a_at	NM_013591	Madcam1	mucosal vascular addressin cell adhesion molecule 1	-6.1	n.s.	-9.1
1418746_at	NM_019999 NM_001039509	Pnkd	Paroxysmal nonkinesiogenic dyskinesia	-5.8	n.s.	-10.3

Affymetrix probe set ID	GeneBank ID	Gene symbol	Gene name	Fold-change versus Wt Tnfr1-/-	Tnfr2-/-	Tnfr1,2-/-
1448632_at	NM_013640	Psmb10	proteasome (prosome, macropain) subunit, beta type 10	-5.7	n.s.	-7.9
1418726_a_at<br>1424967_x_at	NM_011619<br>NM_001130174<br>NM_001130175<br>NM_001130176<br>NM_001130177<br>NM_001130178<br>NM_001130179<br>NM_001130180<br>NM_001130181	Tnnt2	troponin T2, cardiac	-5.6	n.s.	-5.0
1449498_at<br>1458297_s_at	NM_010766	Marco	macrophage receptor with collagenous structure	-5.6	n.s.	-5.0
1424393_s_at<br>1424392_at	NM_175236	Adhfe1	alcohol dehydrogenase, iron containing1	-5.5	n.s.	-5.5
1450047_at	NM_015819	Hs6st2	heparan sulfate 6-O-sulfotransferase 2	-5.4	n.s.	n.s.
1424254_at	NM_026820<br>NM_001112715	Ifitm1	interferon induced transmembrane protein 1	-5.3	n.s.	-3.7
1418126_at	NM_013653	Ccl5	chemokine (C-C motif) ligand 5	-5.2	n.s.	-10.6
1425681_a_at	NM_023043	Prnd	prion protein dublet	-5.1	n.s.	-4.2
1423602_at	NM_009421	Traf1	Tnf receptor-associated factor 1	-4.9	n.s.	-4.8
1416382_at<br>1437939_s_at	NM_009982	Ctsc	cathepsin C	-4.8	n.s.	-5.7
1418847_at<br>1438841_s_at	NM_009705	Arg2	arginase type II	-4.5	n.s.	-6.4
1423091_a_at<br>1425942_a_at	NM_023122	Gpm6b	glycoprotein m6b	-4.5	n.s.	-6.7
1423954_at	NM_009778	C3	complement component 3	-4.5	n.s.	-5.2
1431843_a_at<br>1458299_s_at	NM_008690	Nfkbie	nuclear factor of kappa light polypeptide gene enhancer in B-cells inhibitor, epsilon	-4.5	n.s.	-3.9
1437569_at	NM_023438	Tmem132e	transmembrane protein 132E	-4.4	n.s.	-5.6
1420697_at	NM_023044	Slc15a3	solute carrier family 15, member 3	-4.4	n.s.	-8.8
1426850_a_at	NM_011943	Map2k6	mitogen-activated protein kinase kinase 6	-4.3	n.s.	-6.9
1422962_a_at	NM_010724	Psmb8	proteasome (prosome, macropain) subunit, beta type 8 (large multifunctional peptidase 7)	-4.3	n.s.	-8.0
1439747_at<br>1449450_at	NM_022415	Ptges	prostaglandin E synthase	-4.3	n.s.	-4.2
1416832_at	NM_026228	Slc39a8	solute carrier family 39 (metal ion transporter), member 8	-4.1	n.s.	-3.7
1417262_at<br>1417263_at	NM_011198	Ptgs2	prostaglandin-endoperoxide synthase 2	-4.1	n.s.	-3.8
1416298_at	NM_013599	Mmp9	matrix metallopeptidase 9	-4.0	n.s.	-3.6
1422953_at	NM_008039	Fpr2	formyl peptide receptor 2	-3.9	n.s.	-3.4
1417936_at	NM_011338	Ccl9	chemokine (C-C motif) ligand 9	-3.9	n.s.	-5.4
1450165_at	NM_011408	Slfn2	schlafen 2	-3.8	n.s.	-5.5

Affymetrix probe set ID	GeneBank ID	Gene symbol	Gene name	Fold-change versus Wt Tnfr1-/-	Tnfr2-/-	Tnfr1,2-/-
1417292_at	NM_008330	Ifi47	interferon gamma inducible protein 47	-3.8	n.s.	-6.1
1436659_at	NM_019978	Dclk1	doublecortin-like kinase 1	-3.8	n.s.	n.s.
1460251_at	NM_007987	Fas	Fas (TNF receptor superfamily member 6)	-3.7	n.s.	-3.3
1419658_at	NM_010398	H2-T23	histocompatibility 2, T region locus 23	-3.7	n.s.	-4.6
1424923_at	NM_009251	Serpina3g	serine (or cysteine) peptidase inhibitor, clade A, member 3G	-3.7	n.s.	-9.4
1418718_at<br>1449195_s_at	NM_023158	Cxcl16	chemokine (C-X-C motif) ligand 16	-3.6	n.s.	-6.2
1419132_at	NM_011905	Tlr2	toll-like receptor 2	-3.6	n.s.	-3.8
1436778_at	NM_007807	Cybb	cytochrome b-245, beta polypeptide	-3.5	n.s.	-4.2
1434372_at	NM_001177351	AW112010	expressed sequence AW112010	-3.5	n.s.	-3.6
1419529_at	NM_031252	Il23a	interleukin 23, alpha subunit p19	-3.5	n.s.	-3.2
1421408_at	NM_030691	Igsf6	immunoglobulin superfamily, member 6	-3.5	n.s.	-3.8
1434510_at	NM_011864	Papss2	3'-phosphoadenosine 5'-phosphosulfate synthase 2	-3.4	n.s.	-3.2
1417193_at<br>1417194_at<br>1448610_a_at<br>1454976_at	NM_013671	Sod2	superoxide dismutase 2, mitochondrial	-3.4	n.s.	-4.2
1416625_at	NM_009776	Serping1	serine (or cysteine) peptidase inhibitor, clade G, member 1	-3.4	n.s.	-4.1
1428538_s_at<br>1437902_s_at	NM_027852	Rarres2	retinoic acid receptor responder (tazarotene induced) 2	-3.4	n.s.	-3.6
1422648_at<br>1436555_at	NM_007514<br>NM_001044740	Slc7a2	solute carrier family 7 (cationic amino acid transporter, y+ system), member 2	-3.4	n.s.	-3.5
1435596_at	NM_175437	Pion	pigeon homolog (Drosophila)	-3.3	n.s.	-2.8
1419691_at	NM_009921	Camp	cathelicidin antimicrobial peptide	-3.2	n.s.	-3.3
1418945_at	NM_010809	Mmp3	matrix metallopeptidase 3	-3.2	n.s.	-4.8
1418981_at	NM_009808	Casp12	caspase 12	-3.2	n.s.	-3.8
1417813_at	NM_019777	Ikbke	inhibitor of kappaB kinase epsilon	-3.1	n.s.	-4.2
1418580_at	NM_023386	Rtp4	receptor transporter protein 4	-3.1	n.s.	-3.5
1427313_at	NM_008967	Ptgir	prostaglandin I receptor (IP)	-3.1	n.s.	-4.0
1424524_at	NM_027878	Dram1	DNA-damage regulated autophagy modulator 1	-3.0	n.s.	-4.0
1448734_at	NM_007752	Cp	ceruloplasmin	-3.0	n.s.	-3.8
1451798_at	NM_031167	Il1rn	interleukin 1 receptor antagonist	-3.0	n.s.	-3.2
1443698_at	NM_001037713	Xaf1	XIAP associated factor 1	-2.9	n.s.	-3.0
1417045_at	NM_007544	Bid	BH3 interacting domain death agonist	-2.9	n.s.	-3.1
1417256_at	NM_008607	Mmp13	matrix metallopeptidase 13	-2.9	n.s.	-2.4
1416051_at<br>1457664_x_at	NM_013484	C2	complement component 2 (within H-2S)	-2.9	n.s.	-3.1

Affymetrix probe set ID	GeneBank ID	Gene symbol	Gene name	Fold-change versus Wt Tnfr1-/-	Tnfr2-/-	Tnfr1,2-/-
1419202_at	NM_009977	Cst7	cystatin F (leukocystatin)	-2.9	n.s.	-2.9
1424824_at	NM_198014	Slain1	SLAIN motif family, member 1	-2.9	n.s.	n.s.
1455197_at	NM_172612	Rnd1	Rho family GTPase 1	-2.8	n.s.	-3.8
1435512_at	NM_177716	AI836003	expressed sequence AI836003	-2.8	n.s.	n.s.
1449227_at	NM_009890	Ch25h	cholesterol 25-hydroxylase	-2.8	n.s.	n.s.
1448201_at	NM_009144	Sfrp2	secreted frizzled-related protein 2	-2.8	n.s.	-3.0
1431055_a_at	NM_028035	Snx10	sorting nexin 10	-2.8	n.s.	-2.5
1420499_at 1429692_s_at	NM_008102	Gch1	GTP cyclohydrolase 1	-2.8	n.s.	-2.9
1437312_at 1443720_s_at	NM_007560	Bmpr1b	bone morphogenetic protein receptor, type 1B	-2.7	n.s.	-3.4
1427482_a_at	NM_007592	Car8	carbonic anhydrase 8	-2.7	n.s.	n.s.
1451567_a_at	NM_008328 NM_001045481	Ifi203	interferon activated gene 203	-2.7	n.s.	-2.3
1421366_at	NM_021364 NM_001038604	Clec5a	C-type lectin domain family 5, member a	-2.7	n.s.	-2.8
1427689_a_at	NM_021327	Tnip1	TNFAIP3 interacting protein 1	-2.7	n.s.	-3.4
1425951_a_at	NM_020001	Clec4n	C-type lectin domain family 4, member n	-2.7	n.s.	-4.1
1437442_at 1449249_at	NM_018764	Pcdh7	protocadherin 7	-2.7	n.s.	n.s.
1426037_a_at	NM_011267	Rgs16	regulator of G-protein signaling 16	-2.7	n.s.	-2.6
1443827_x_at	NM_030565	Fam20c	family with sequence similarity 20, member C	-2.7	n.s.	-2.7
1420413_at	NM_011990	Slc7a11	solute carrier family 7 (cationic amino acid transporter, y+ system), member 11	-2.7	n.s.	-2.7
1442116_at	NM_201367	Gpr176	G protein-coupled receptor 176	-2.6	n.s.	n.s.
1419534_at	NM_138648	Olr1	oxidized low density lipoprotein (lectin-like) receptor 1	-2.6	n.s.	-2.5
1422835_at	NM_019697	Kcnd2	potassium voltage-gated channel, Shal-related family, member 2	-2.6	n.s.	n.s.
1429796_at 1436066_at	NM_177357 NM_001164268	Kalrn	kalirin, RhoGEF kinase	-2.5	n.s.	-2.7
1451544_at	NM_145391	Tapbpl	TAP binding protein-like	-2.5	n.s.	-3.2
1451564_at	NM_001039530	Parp14	poly (ADP-ribose) polymerase family, member 14	-2.5	n.s.	-3.9
1417822_at	NM_033075	D17H6S56E-5	DNA segment, Chr 17, human D6S56E 5	-2.5	n.s.	-2.4
1448793_a_at	NM_011521	Sdc4	syndecan 4	-2.5	n.s.	n.s.
1417189_at	NM_011190 NM_001029855	Psme2	proteasome (prosome, macropain) 28 subunit, beta	-2.5	n.s.	-2.7
1435208_at	NM_001013371	Dtx3l	deltex 3-like (Drosophila)	-2.5	n.s.	-2.4
1416527_at	NM_026405	Rab32	RAB32, member RAS oncogene family	-2.5	n.s.	-2.4
1448550_at	NM_008489	Lbp	lipopolysaccharide binding protein	-2.5	n.s.	-2.5

Affymetrix probe set ID	GeneBank ID	Gene symbol	Gene name	Fold-change versus Wt Tnfr1-/-	Tnfr2-/-	Tnfr1,2-/-
1433699_at	NM_009397	Tnfaip3	tumor necrosis factor alpha-induced protein 3	-2.5	n.s.	-3.9
1435349_at	NM_010939	Nrp2	neuropilin 2	-2.4	n.s.	-3.1
1421812_at	NM_009318 NM_001025313	Tapbp	TAP binding protein	-2.4	n.s.	-2.9
1436999_at	NM_001003948	Pid1	phosphotyrosine interaction domain containing 1	-2.4	n.s.	n.s.
1436559_a_at	NM_016883 NM_001164177	Psmd10	proteasome (prosome, macropain) 26S subunit, non-ATPase, 10	-2.4	n.s.	n.s.
1437932_a_at	NM_016674	Cldn1	claudin 1	-2.4	n.s.	n.s.
1426223_at	NM_028341	Ttc39c	tetratricopeptide repeat domain 39C	-2.4	n.s.	-2.5
1455251_at	NM_001033228	Itga1	integrin alpha 1	-2.4	n.s.	-2.3
1417172_at	NM_019949	Ube2l6	ubiquitin-conjugating enzyme E2L 6	-2.3	n.s.	-2.3
1428767_at	NM_026960	Gsdmd	gasdermin D	-2.3	n.s.	-2.6
1449399_a_at	NM_008361	Il1b	interleukin 1 beta	-2.3	n.s.	-1.7
1450297_at	NM_031168	Il6	interleukin 6	-2.3	n.s.	n.s.
1451474_a_at	NM_001081009	Parp8	poly (ADP-ribose) polymerase family, member 8	-2.2	n.s.	-2.2
1416645_a_at	NM_007423	Afp	alpha fetoprotein	-2.2	n.s.	n.s.
1425669_at 1429284_at	NM_178061	Mobkl2b	MOB1, Mps One Binder kinase activator-like 2B (yeast)	-2.2	n.s.	-2.2
1416897_at	NM_030253	Parp9	poly (ADP-ribose) polymerase family, member 9	-2.2	n.s.	-2.7
1424613_at	NM_022420	Gprc5b	G protein-coupled receptor, family C, group 5, member B	-2.2	n.s.	n.s.
1419080_at	NM_010275	Gdnf	glial cell line derived neurotrophic factor	-2.2	n.s.	-2.8
1460415_a_at	NM_011611 NM_170701 NM_170702 NM_170703 NM_170704	Cd40	CD40 antigen	-2.1	n.s.	n.s.
1420360_at	NM_010051	Dkk1	dickkopf homolog 1 (Xenopus laevis)	-2.1	n.s.	-1.9
1416942_at	NM_030711	Erap1	endoplasmic reticulum aminopeptidase 1	-2.1	n.s.	-1.8
1421236_at 1450173_at	NM_138952	Ripk2	receptor (TNFRSF)-interacting serine-threonine kinase 2	-2.1	n.s.	-2.2
1429527_a_at 1453181_x_at	NM_011636	Plscr1	phospholipid scramblase 1	-2.1	n.s.	-2.4
1440840_at	AK052609	D630004K 10Rik	RIKEN cDNA D630004K10 gene	-2.1	n.s.	n.s.
1453004_at	NM_001033167	Slc22a23	solute carrier family 22, member 23	-2.0	n.s.	n.s.
1416295_a_at	NM_013563	Il2rg	interleukin 2 receptor, gamma chain	-2.0	n.s.	-2.9
1454834_at	NM_008687	Nfib	nuclear factor I/B	-2.0	n.s.	-2.4
1435488_at	NM_026788	Mthfd2l	methylenetetrahydrofolate dehydro-genase (NADP+ dependent) 2-like	-2.0	n.s.	-2.1

Affymetrix probe set ID	GeneBank ID	Gene symbol	Gene name	Fold-change versus Wt Tnfr1-/-	Tnfr2-/-	Tnfr1,2-/-
1460197_a_at	NM_054098	Steap4	STEAP family member 4	-2.0	n.s.	-2.2
1455358_at	NM_021477 NM_183188	A2bp1	ataxin 2 binding protein 1	-2.0	n.s.	-1.7
1454974_at	NM_008744	Ntn1	netrin 1	-1.9	n.s.	-1.6
1437785_at	NM_175314	Adamts9	a disintegrin-like and metallopeptidase (reprolysin type) with thrombospondin type 1 motif, 9	-1.9	n.s.	-1.8
1441493_at	NM_053204 NM_178085	Erc1	ELKS/RAB6-interacting/CAST family member 1	-1.9	n.s.	n.s.
1455711_at	NM_172442	Dtx4	deltex 4 homolog (Drosophila)	-1.8	n.s.	-2.0
1460081_at	NM_018801 NM_173067 NM_173068	Syt7	synaptotagmin VII	-1.8	n.s.	-1.8
1425767_a_at	NM_011382	Six4	sine oculis-related homeobox 4 homolog (Drosophila)	-1.8	n.s.	-1.6
1418004_a_at	NM_023056	Tmem176b	transmembrane protein 176B	-1.8	n.s.	n.s.
1426501_a_at	NM_145133	Tifa	TRAF-interacting protein with forkhead-associated domain	-1.8	n.s.	-2.1
1440230_at	NM_001024619	Tsku	tsukushin	-1.8	n.s.	n.s.
1416998_at	NM_021511	Rrs1	RRS1 ribosome biogenesis regulator homolog (S. cerevisiae)	-1.8	n.s.	-1.7
1429974_at	NM_023814	Tbx18	T-box18	-1.8	n.s.	n.s.
1417786_a_at	NM_026446	Rgs19	regulator of G-protein signaling 19	-1.7	n.s.	n.s.
1437743_at	NM_009637 NM_178803 NM_001005605	Aebp2	AE binding protein 2	-1.7	n.s.	n.s.
1421858_at	NM_009615	Adam17	a disintegrin and metallopeptidase domain 17	-1.7	n.s.	-1.9
1455398_at	NM_133897	Lrrc8c	leucine rich repeat containing 8 family, member C	-1.7	n.s.	n.s.
1460014_at	NM_172623 NM_001033922	Treml4	triggering receptor expressed on myeloid cells-like 4	-1.6	n.s.	-1.8
1428891_at	NM_145562	Parm1	prostate androgen-regulated mucin-like protein 1	-1.6	n.s.	n.s.
1432026_a_at	NM_025992	Herc6	hect domain and RLD 6	-1.6	n.s.	n.s.
1417704_a_at	NM_009707 NM_178754	Arhgap6	Rho GTPase activating protein 6	1.8	n.s.	n.s.
1432667_at	AK008898	2210411G 17Rik	RIKEN cDNA 2210411G17 gene	1.8	n.s.	n.s.
1449863_a_at	NM_198854 NM_010056	Dlx5	distal-less homeobox 5	2.0	n.s.	1.8
1434248_at	NM_008856	Prkch	protein kinase C, eta	2.1	n.s.	n.s.
1448831_at	NM_007426	Angpt2	angiopoietin 2	2.1	n.s.	n.s.
1434678_at	NM_134163	Mbnl3	muscleblind-like 3 (Drosophila)	2.1	n.s.	n.s.
1418183_a_at	NM_011180	Pscd1	pleckstrin homology, Sec7 and coiled-coil domains 1	2.2	n.s.	n.s.

Affymetrix probe set ID	GeneBank ID	Gene symbol	Gene name	Fold-change versus Wt Tnfr1-/-	Tnfr2-/-	Tnfr1,2-/-
1425518_at	NM_019688	Rapgef4	Rap guanine nucleotide exchange factor (GEF) 4	2.2	n.s.	2.3
1454795_at	NM_177025	Cobll1	Cobl-like 1	2.2	n.s.	n.s.
1422906_at	NM_011920	Abcg2	ATP-binding cassette, sub-family G (WHITE), member 2	2.2	n.s.	2.5
1424852_at	NM_025282	Mef2c	myocyte enhancer factor 2C	2.3	n.s.	2.2
1419447_s_at	NM_019636	Tbc1d1	TBC1 domain family, member 1	2.3	n.s.	n.s.
1416129_at	NM_133753	Errfi1	ERBB receptor feedback inhibitor 1	2.3	n.s.	2.4
1419758_at	NM_011076	Abcb1a	ATP-binding cassette, sub-family B (MDR/TAP), member 1A	2.3	n.s.	2.0
1438325_at	NM_007963	Evi1	ecotropic viral integration site 1	2.4	n.s.	2.3
1440888_at	NM_001081147	Oxtr	oxytocin receptor	2.5	n.s.	n.s.
1455361_at	NM_178681	Dgkb	diacylglycerol kinase, beta	2.5	n.s.	2.2
1456096_at	NM_176952	6430573F 11Rik	RIKEN cDNA 6430573F11 gene	2.5	n.s.	n.s.
1435697_a_at 1451206_s_at	NM_139200	Pscdbp	pleckstrin homology, Sec7 and coiled-coil domains, binding protein	2.5	n.s.	n.s.
1449396_at	NM_009675	Aoc3	amine oxidase, copper containing 3	2.5	n.s.	2.9
1445723_at	NM_009658	Plcl1	phospholipase C-like 1	2.6	n.s.	n.s.
1425514_at	NM_011085 NM_001024955	Pik3r1	phosphatidylinositol 3-kinase, regulatory subunit, polypeptide 1 (p85 alpha)	2.6	n.s.	n.s.
1416105_at	NM_008710	Nnt	nicotinamide nucleotide transhydrogenase	2.6	n.s.	n.s.
1455050_at	NM_178791	E130203B 14Rik	RIKEN cDNA E130203B14 gene	2.6	n.s.	2.7
1435184_at	NM_008728 NM_001039181	Npr3	natriuretic peptide receptor 3	2.7	n.s.	2.9
1454966_at	NM_001001309	Itga8	integrin alpha 8	2.7	n.s.	1.9
1455056_at	NM_201529	Lmo7	LIM domain only 7	2.7	n.s.	n.s.
1435741_at	NM_172263	Pde8b	phosphodiesterase 8B	2.7	n.s.	2.1
1418142_at	NM_008428	Kcnj8	potassium inwardly-rectifying channel, subfamily J, member 8	2.8	n.s.	n.s.
1417622_at 1448780_at	NM_009194	Slc12a2	solute carrier family 12, member 2	2.8	n.s.	2.1
1454942_at	NM_022018	Niban	niban protein	2.8	n.s.	3.3
1455521_at	NM_010636	Klf12	Kruppel-like factor 12	2.9	n.s.	n.s.
1442115_at	NM_001039485	Fam38b	family with sequence similarity 38, member B	2.9	n.s.	n.s.
1416164_at	NM_011812	Fbln5	fibulin 5	2.9	n.s.	2.7
1417061_at	NM_016917	Slc40a1	solute carrier family 40 (iron-regulated transporter), member 1	3.0	n.s.	n.s.
1418156_at	NM_021342	Kcne4	potassium voltage-gated channel, Isk-related subfamily, gene 4	3.0	n.s.	3.4

Affymetrix probe set ID	GeneBank ID	Gene symbol	Gene name	Fold-change versus Wt Tnfr1-/-	Tnfr2-/-	Tnfr1,2-/-
1440355_at	NM_175429	Kctd12b	potassium channel tetramerisation domain containing 12b	3.0	n.s.	3.9
1420534_at<br>1434141_at	NM_021896	Gucy1a3	guanylate cyclase 1, soluble, alpha 3	3.0	n.s.	2.6
1435106_at<br>1435321_at	NM_001001980	Limch1	LIM and calponin homology domains 1	3.1	n.s.	2.9
1426622_a_at	NM_027455	Qpct	glutaminyl-peptide cyclotransferase (glutaminyl cyclase)	3.1	n.s.	3.5
1422824_s_at	NM_007945	Eps8	epidermal growth factor receptor pathway substrate 8	3.1	n.s.	n.s.
1416892_s_at<br>1448509_at	NM_025626	Fam107b	family with sequence similarity 107, member B	3.2	n.s.	2.7
1438651_a_at	NM_011784	Agtrl1	angiotensin receptor-like 1	3.3	n.s.	n.s.
1445223_at	NM_028460<br>NM_001032413<br>NM_001032414	Pear1	platelet endothelial aggregation receptor 1	3.4	n.s.	n.s.
1441065_at	NM_009167	Shc3	Src homology 2 domain-containing transforming protein C3	3.5	n.s.	n.s.
1419248_at	NM_009061	Rgs2	regulator of G-protein signaling 2	3.7	n.s.	n.s.
1455627_at	NM_007739	Col8a1	collagen, type VIII, alpha 1	3.8	n.s.	n.s.
1420838_at<br>1435196_at	NM_008745<br>NM_001025074	Ntrk2	neurotrophic tyrosine kinase, receptor, type 2	4.0	n.s.	2.9
1429579_at	NM_001033322	Gucy1a2	guanylate cyclase 1, soluble, alpha 2	4.1	n.s.	4.0
1422084_at	NM_009759	Bmx	BMX non-receptor tyrosine kinase	4.2	n.s.	3.6
1425846_a_at	NM_021371	Caln1	calneuron 1	4.5	n.s.	4.1
1424007_at	NM_145741	Gdf10	growth differentiation factor 10	4.8	n.s.	n.s.
1419405_at	NM_026523	Nmb	neuromedin B	5.0	n.s.	5.1
1433691_at	NM_016854	Ppp1r3c	protein phosphatase 1, regulatory (inhibitor) subunit 3C	5.3	n.s.	6.0
1435752_s_at	NM_011511<br>NM_021041<br>NM_021042<br>NM_001044720	Abcc9	ATP-binding cassette, sub-family C (CFTR/MRP), member 9	5.4	n.s.	3.1
1422454_at	NM_010662	Krt13	keratin 13	5.7	n.s.	6.5
1428662_a_at<br>1451776_s_at	NM_175606	Hopx	HOP homeobox	5.9	n.s.	5.7
1419302_at<br>1438886_at	NM_013905	Heyl	hairy/enhancer-of-split related with YRPW motif-like	6.0	n.s.	4.7
1438953_at<br>1438954_x_at<br>1449528_at	NM_010216	Figf	C-fos induced growth factor	6.4	n.s.	5.3
1428664_at	NM_011702	Vip	vasoactive intestinal polypeptide	9.5	n.s.	11.2

[1]Genes are listed according to fold-change of *Tnfr1*-/- versus wildtype (Wt) glomeruli. For each gene all identifying probe sets are listed. Multiple GeneBank IDs are given for probe sets detecting multiple transcripts of one gene. n.s.: not significant.
