# Supplementary material for: Distinct Contributions of TNF Receptor 1 and 2 to TNF-Induced Glomerular Inflammation in Mice
Source: PLoS One. 2013 Jul 15;8(7):e68167. doi: 10.1371/journal.pone.0068167 (PMC3711912; doi:10.1371/journal.pone.0068167)
Supplement: Table S4 — Differentially expressed genes in TNF-stimulated Tnfr2−/− glomeruli compared to wildtype as identified by microarray profiling. (PDF) [file pone.0068167.s005.pdf]

Table S4. Differentially expressed genes in TNF-stimulated *Tnfr2-/-* glomeruli compared to wildtype (Wt) as identified by microarray profiling[1].

Affymetrix probe set ID	GeneBank ID	Gene symbol	Gene name	Fold-change versus Wt Tnfr2-/-	Tnfr1-/-	Tnfr1,2-/-
1418099_at	NM_011610	Tnfrsf1b	tumor necrosis factor receptor superfamily, member 1b	-4.9	n.s.	-4.4
1418778_at	NM_025779	Ccdc109b	coiled-coil domain containing 109B	-4.3	n.s.	n.s.
1424784_at	NM_001083918	OTTMUSG00000010657	predicted gene, OTTMUSG00000010657	-3.5	n.s.	-3.2
1436574_at	NM_027285	1700029I01Rik	RIKEN cDNA 1700029I01 gene	-3.1	n.s.	-3.4
1434914_at	NM_173781	Rab6b	RAB6B, member RAS oncogene family	-2.6	n.s.	-3.2

[1]Genes are listed according to fold-change of *Tnfr2-/-* versus wildtype (Wt) glomeruli.
n.s.: not significant.
