# Supplementary material for: Distinct Contributions of TNF Receptor 1 and 2 to TNF-Induced Glomerular Inflammation in Mice
Source: PLoS One. 2013 Jul 15;8(7):e68167. doi: 10.1371/journal.pone.0068167 (PMC3711912; doi:10.1371/journal.pone.0068167)
Supplement: Table S5 — Enriched functional groups of differentially expressed genes in TNF-stimulated Tnfr1,2−/− glomeruli compared to wildtype as identified by DAVID. (PDF) [file pone.0068167.s006.pdf]

Table S5. Enriched functional groups of differentially expressed genes in TNF-stimulated *Tnfr1,2-/-* glomeruli compared to wildtype (Wt) as identified by DAVID[1]

GeneBank ID	Gene symbol	Gene name	Fold-change Tnfr1,2-/- versus Wt
Functional group 1: Chemokines and cytokines (enrichment score 8.86)			
NM_023158	Cxcl16	chemokine (C-X-C motif) ligand 16	-6.2
NM_203320	Cxcl3	chemokine (C-X-C motif) ligand 3	-22.4
NM_009140	Cxcl2	chemokine (C-X-C motif) ligand 2	-24.9
NM_009141	Cxcl5	chemokine (C-X-C motif) ligand 5	-7.8
NM_008599	Cxcl9	chemokine (C-X-C motif) ligand 9	-5.4
NM_021274	Cxcl10	chemokine (C-X-C motif) ligand 10	-21.0
NM_008176	Cxcl1	chemokine (C-X-C motif) ligand 1	-3.7
NM_011333	Ccl2	chemokine (C-C motif) ligand 2	-50.0
NM_031252	Il23a	interleukin 23, alpha subunit p19	-3.2
NM_013653	Ccl5	chemokine (C-C motif) ligand 5	-10.6
NM_011338	Ccl9	chemokine (C-C motif) ligand 9	-5.4
NM_013654	Ccl7	chemokine (C-C motif) ligand 7	-11.2
Functional group 2: Integral membrane molecules and receptors (enrichment score: 4.57)			
NM_011693	Vcam1	vascular cell adhesion molecule 1	-23.8
NM_009318	Tapbp	TAP binding protein	-2.9
NM_010493	Icam1	intercellular adhesion molecule 1	-15.4
NM_145391	Tapbpl	TAP binding protein-like	-3.2
NM_009472	Unc5c	unc-5 homolog C (C. elegans)	-1.9
NM_010398	H2-T23	histocompatibility 2, T region locus 23	-4.6
NM_019909	H2-K1	histocompatibility 2, K1, K region	-2.1
NM_008967	Ptgir	prostaglandin I receptor (IP)	-4.0
NM_010939	Nrp2	neuropilin 2	-3.1
NM_007904	Ednrb	endothelin receptor type B	2.2
NM_011920	Abcg2	ATP-binding cassette, sub-family G (WHITE), member 2	2.5
NM_011610	Tnfrsf1b	tumor necrosis factor receptor superfamily, member 1b	-4.4
NM_013563	Il2rg	interleukin 2 receptor, gamma chain	-2.9
NM_025638	Gdpd1	glycerophosphodiester phosphodiesterase domain containing 1	-1.8
NM_177322	Agtr1a	angiotensin II receptor, type 1a	-1.9
NM_011609	Tnfrsf1a	tumor necrosis factor receptor superfamily, member 1a	-2.4

GeneBank ID	Gene symbol	Gene name	Fold-change Tnfr1,2-/- versus Wt
NM_023122	Gpm6b	glycoprotein m6b	-6.7
NM_007781	Csf2rb2	colony stimulating factor 2 receptor, beta 2, low-affinity (granulocyte-macrophage)	-1.7
NM_007780	Csf2rb	colony stimulating factor 2 receptor, beta, low-affinity (granulocyte-macrophage)	-1.8
NM_010509	Ifnar2	interferon (alpha and beta) receptor 2	-3.1
NM_175316	Slco2b1	solute carrier organic anion transporter family, member 2b1	-5.1
NM_011990	Slc7a11	solute carrier family 7 (cationic amino acid transporter, y+ system), member 11	-2.7
NM_013521	Fpr1	formyl peptide receptor 1	-2.2
NM_010766	Marco	macrophage receptor with collagenous structure	-5.0
NM_020001	Clec4n	C-type lectin domain family 4, member n	-4.1
NM_021364	Clec5a	C-type lectin domain family 5, member a	-2.8
NM_019948	Clec4e	C-type lectin domain family 4, member e	-13.2
NM_010819	Clec4d	C-type lectin domain family 4, member d	-3.2
NM_138648	Olr1	oxidized low density lipoprotein (lectin-like) receptor 1	-2.5
NM_025809	Clec14a	C-type lectin domain family 14, member a	-2.2
NM_001033534	Layn	layilin	-1.7
NM_054042	Cd248	CD248 antigen, endosialin	2.5
NM_008489	Lbp	lipopolysaccharide binding protein	-2.5
NM_130456	Nphs2	nephrosis 2 homolog, podocin (human)	2.0
NM_029771	Gper	G protein-coupled estrogen receptor 1	-1.6
NM_029084	Slamf8	SLAM family member 8	-4.9
NM_013591	Madcam1	mucosal vascular addressin cell adhesion molecule 1	-9.1
NM_007514	Slc7a2	solute carrier family 7 (cationic amino acid transporter, y+ system), member 2	-3.5
NM_009404	Tnfsf9	tumor necrosis factor (ligand) superfamily, member 9	-1.9
NM_025326	Tmem176a	transmembrane protein 176A	-1.9
NM_025359	Tspan13	tetraspanin 13	2.0
NM_030720	Gpr84	G protein-coupled receptor 84	-16.6
NM_017466	Ccrl2	chemokine (C-C motif) receptor-like 2	-2.9
NM_023386	Rtp4	receptor transporter protein 4	-3.5
NM_023438	Tmem132e	transmembrane protein 132E	-5.6
NM_177709	Tusc5	tumor suppressor candidate 5	-1.9

GeneBank ID	Gene symbol	Gene name	Fold-change Tnfr1,2-/- versus Wt
Functional group 3: Innate immune effectors (enrichment score: 3.89)			
NM_009778	C3	complement component 3	-5.2
NM_023143	C1r	complement component 1, r subcomponent	-1.8
NM_009776	Serping1	serine (or cysteine) peptidase inhibitor, clade G, member 1	-4.1
NM_007972	F10	coagulation factor X	-2.2
NM_013484	C2	complement component 2 (within H-2S)	-3.1
NM_008198	Cfb	complement factor B	-35.2
NM_017370	Hp	haptoglobin	-13.0
Functional group 4: Matrix metallopeptidases (enrichment score: 3.37)			
NM_008605	Mmp12	matrix metallopeptidase 12	-2.5
NM_008607	Mmp13	matrix metallopeptidase 13	-2.4
NM_013599	Mmp9	matrix metallopeptidase 9	-3.6
NM_001003911	Adamts7	a disintegrin-like and metallopeptidase (reprolysin type) with thrombospondin type 1 motif, 7	-6.5
NM_009615	Adam17	a disintegrin and metallopeptidase domain 17	-1.9
NM_010809	Mmp3	matrix metallopeptidase 3	-4.8
NM_027455	Qpct	glutaminyl-peptide cyclotransferase (glutaminyl cyclase)	3.5
Functional group 5: Metabolic enzymes, zink ion binding (enrichment score: 2.62)			
NM_172827	Lnpep	leucyl/cystinyl aminopeptidase	1.5
NM_080563	Rnf144a	ring finger protein 144A	1.9
NM_175314	Adamts9	a disintegrin-like and metallopeptidase (reprolysin type) with thrombospondin type 1 motif, 9	-1.8
NM_001003911	Adamts7	a disintegrin-like and metallopeptidase (reprolysin type) with thrombospondin type 1 motif, 7	-6.5
Functional group 6: Cellular protein processing (enrichment score: 1.53)			
NM_001037713	Xaf1	XIAP associated factor 1	-3.0
NM_016808	Usp2	ubiquitin specific peptidase 2	1.8
NM_023738	Ube1l	ubiquitin-activating enzyme E1-like	-2.5
NM_010724	Psmb8	proteasome (prosome, macropain) subunit, beta type 8 (large multifunctional peptidase 7)	-8.0
NM_019949	Ube2l6	ubiquitin-conjugating enzyme E2L 6	-2.4

GeneBank ID	Gene symbol	Gene name	Fold-change Tnfr1,2-/- versus Wt
NM_013585	Psmb9	proteasome (prosome, macropain) subunit, beta type 9 (large multifunctional peptidase 2)	-3.9
NM_013640	Psmb10	proteasome (prosome, macropain) subunit, beta type 10	-7.9
NM_080563	Rnf144a	ring finger protein 144A	1.9
NM_001081009	Parp8	poly (ADP-ribose) polymerase family, member 8	-2.2
Functional group 7: Zinc ion binding proteins (enrichment score: 1.39)			
NM_001001980	Limch1	LIM and calponin homology domains 1	2.9
NM_019417	Pdlim4	PDZ and LIM domain 4	-1.9
NM_001013371	Dtx3l	deltex 3-like (Drosophila)	-2.4
NM_177086	Zmat4	zinc finger, matrin type 4	2.3
NM_172442	Dtx4	deltex 4 homolog (Drosophila)	-2.0
NM_001134741	Tdrd5	tudor domain containing 5	2.2
NM_172397	Limd2	LIM domain containing 2	-1.5

[1]The gene functional classification tool of DAVID was used to rank the overall importance of functional gene groups that reached an enrichment score of ≥1.3 (p-value <0.05) as described in Materials and Methods.
