# Supplementary material for: Distinct Contributions of TNF Receptor 1 and 2 to TNF-Induced Glomerular Inflammation in Mice
Source: PLoS One. 2013 Jul 15;8(7):e68167. doi: 10.1371/journal.pone.0068167 (PMC3711912; doi:10.1371/journal.pone.0068167)
Supplement: Table S6 — Enriched functional groups of differentially expressed genes in TNF-stimulated Tnfr1−/− glomeruli compared to wildtype as identified by DAVID. (PDF) [file pone.0068167.s007.pdf]

Table S6. Enriched functional groups of differentially expressed genes in TNF-stimulated *Tnfr1-/-* glomeruli compared to wildtype (Wt) as identified by DAVID[1].

GeneBank ID	Gene symbol	Gene name	Fold-change Tnfr1-/- versus Wt
Functional group 1: Chemokines and cytokines (enrichment score 7.61)			
NM_023158	Cxcl16	chemokine (C-X-C motif) ligand 16	-3.6
NM_203320	Cxcl3	chemokine (C-X-C motif) ligand 3	-19.4
NM_009140	Cxcl2	chemokine (C-X-C motif) ligand 2	-18.0
NM_009141	Cxcl5	chemokine (C-X-C motif) ligand 5	-8.3
NM_008599	Cxcl9	chemokine (C-X-C motif) ligand 9	-14.0
NM_021274	Cxcl10	chemokine (C-X-C motif) ligand 10	-15.0
NM_011333	Ccl2	chemokine (C-C motif) ligand 2	-42.9
NM_031252	Il23a	interleukin 23, alpha subunit p19	-3.5
NM_013653	Ccl5	chemokine (C-C motif) ligand 5	-5.2
NM_011338	Ccl9	chemokine (C-C motif) ligand 9	-3.9
NM_013654	Ccl7	chemokine (C-C motif) ligand 7	-14.7
Functional group 2: Integral membrane molecules and receptors (enrichment score: 3.65)			
NM_011920	Abcg2	ATP-binding cassette, sub-family G (WHITE), member 2	2.2
NM_009318	Tapbp	TAP binding protein	-2.4
NM_011990	Slc7a11	solute carrier family 7 (cationic amino acid transporter, y+ system), member 11	-2.7
NM_007514	Slc7a2	solute carrier family 7 (cationic amino acid transporter, y+ system), member 2	-3.4
NM_010766	Marco	macrophage receptor with collagenous structure	-5.6
NM_030720	Gpr84	G protein-coupled receptor 84	-19.0
NM_008967	Ptgir	prostaglandin I receptor (IP)	-3.1
NM_201367	Gpr176	G protein-coupled receptor 176	-2.6
NM_010939	Nrp2	neuropilin 2	-2.4
NM_011784	Agtrl1	angiotensin receptor-like 1	3.3
NM_001081147	Oxtr	oxytocin receptor	2.5
NM_022420	Gprc5b	G protein-coupled receptor, family C, group 5, member B	-2.2
NM_138648	Olr1	oxidized low density lipoprotein (lectin-like) receptor 1	-2.6
NM_010493	Icam1	intercellular adhesion molecule 1	-9.9
NM_010398	H2-T23	histocompatibility 2, T region locus 23	-3.7
NM_145391	Tapbpl	TAP binding protein-like	-2.5

GeneBank ID	Gene symbol	Gene name	Fold-change Tnfr1-/- versus Wt
NM_013591	Madcam1	mucosal vascular addressin cell adhesion molecule 1	-6.1
NM_001033167	Slc22a23	solute carrier family 22, member 23	-2.0
NM_011521	Sdc4	syndecan 4	-2.5
NM_008489	Lbp	lipopolysaccharide binding protein	-2.5
NM_011693	Vcam1	vascular cell adhesion molecule 1	-22.1
NM_019948	Clec4e	C-type lectin domain family 4, member e	-10.2
NM_023122	Gpm6b	glycoprotein m6b	-4.5
NM_133897	Lrrc8c	leucine rich repeat containing 8 family, member C	-1.7
NM_020001	Clec4n	C-type lectin domain family 4, member n	-2.7
NM_021364	Clec5a	C-type lectin domain family 5, member a	-2.7
NM_023056	Tmem176b	transmembrane protein 176B	-1.8
NM_175316	Slco2b1	solute carrier organic anion transporter family, member 2b1	-14.3
NM_015819	Hs6st2	heparan sulfate 6-O-sulfotransferase 2	-5.4
NM_145562	Parm1	prostate androgen-regulated mucin-like protein 1	-1.6
NM_023386	Rtp4	receptor transporter protein 4	-3.1
NM_023438	Tmem132e	transmembrane protein 132E	-4.4
NM_001039485	Fam38b	family with sequence similarity 38, member B	2.9
Functional group 3: Innate immune effectors (enrichment score: 2.77)			
NM_009778	C3	complement component 3	-4.5
NM_009776	Serping1	serine (or cysteine) peptidase inhibitor, clade G, member 1	-3.4
NM_013484	C2	complement component 2 (within H-2S)	-2.9
NM_008198	Cfb	complement factor B	-34.0
NM_017370	Hp	haptoglobin	-15.0
Functional group 4: Matrix metallopeptidases (enrichment score: 2.05)			
NM_013599	Mmp9	matrix metallopeptidase 9	-4.0
NM_008607	Mmp13	matrix metallopeptidase 13	-2.9
NM_009615	Adam17	a disintegrin and metallopeptidase domain 17	-1.7
NM_010809	Mmp3	matrix metallopeptidase 3	-3.2

GeneBank ID	Gene symbol	Gene name	Fold-change Tnfr1-/- versus Wt
Functional group 5: Zinc ion binding proteins (enrichment score: 1.66)			
NM_007592	Car8	carbonic anhydrase 8	-2.7
NM_172442	Dtx4	deltex 4 homolog (Drosophila)	-1.8
NM_001013371	Dtx3l	deltex 3-like (Drosophila)	-2.5
NM_178061	Mobkl2b	MOB1, Mps one binder kinase activator-like 2B (yeast)	-2.2
Functional group 6: Ion transport proteins (enrichment score: 1.38)			
NM_019697	Kcnd2	potassium voltage-gated channel, Shal-related family, member 2	-2.6
NM_007807	Cybb	cytochrome b-245, beta polypeptide	-3.5
NM_016917	Slc40a1	solute carrier family 40 (iron-regulated transporter), member 1	3.0
NM_009194	Slc12a2	solute carrier family 12, member 2	2.8
NM_021342	Kcne4	potassium voltage-gated channel, Isk-related subfamily, gene 4	3.0
NM_008428	Kcnj8	potassium inwardly-rectifying channel, subfamily J, member 8	2.8
Functional group 7: Negative regulators of G-protein signaling (enrichment score: 1.37)			
NM_009061	Rgs2	regulator of G-protein signaling 2	3.7
NM_011267	Rgs16	regulator of G-protein signaling 16	-2.7
NM_026446	Rgs19	regulator of G-protein signaling 19	-1.7
NM_019958	Rgs17	regulator of G-protein signaling 17	-8.0

[1]The gene functional classification tool of DAVID was used to rank the overall importance of functional gene groups that reached an enrichment score of ≥1.3 (p-value <0.05) as described in Materials and Methods.
